# Ligand-enabled oxidation of gold(i) complexes with *o*-quinones†

**DOI:** 10.1039/d2sc03724f

**Published:** 2022-08-05

**Authors:** György Szalóki, Julien Babinot, Vlad Martin-Diaconescu, Sonia Mallet-Ladeira, Yago García-Rodeja, Karinne Miqueu, Didier Bourissou

**Affiliations:** Laboratoire Hétérochimie Fondamentale et Appliquée (LHFA, UMR 5069), CNRS, Université Toulouse III – Paul Sabatier 118 Route de Narbonne Toulouse 31062 Cedex 09 France didier.bourissou@univ-tlse3.fr; ALBA Synchrotron – CELLS Carrer de la Llum 2-26 Cerdanyola del Vallès 08290 Barcelona Spain; Institut de Chimie de Toulouse (UAR 2599) 118 Route de Narbonne Toulouse 31062 Cedex 09 France; Institut des Sciences Analytiques et Physico-Chimie pour l’Environnement et les Matériaux (IPREM, UMR 5254), CNRS, Université de Pau et des Pays de l’Adour E2S UPPA, Hélioparc 2 Avenue du Président Angot Pau 64053 Cedex 09 France

## Abstract

Chelating P^P and hemilabile P^N ligands were found to trigger the oxidation of Au(i) complexes by *o*-benzoquinones. The ensuing Au(iii) catecholate complexes have been characterized by NMR spectroscopy, single crystal X-ray diffraction and X-ray absorption spectroscopy. They adopt tetracoordinate square-planar structures. Reactivity studies substantiate the reversibility of the transformation. In particular, the addition of competing ligands such as chloride and alkenes gives back Au(i) complexes with concomitant release of the *o*-quinone. DFT calculations provide insight about the structure and relative stability of the Au(i) *o*-quinone and Au(iii) catecholate forms, and shed light on the 2-electron transfer from gold to the *o*-quinone.

## Abstract 2

Chelating P^P and hemilabile P^N ligands were found to trigger the oxidation of Au(i) complexes by *o*-benzoquinones.

## Graphical abstract

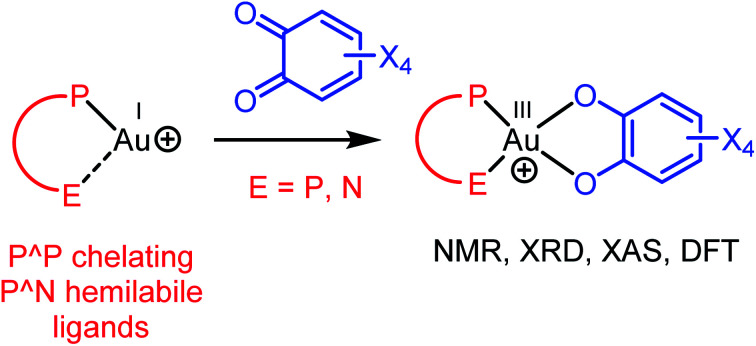

## Introduction

Long considered too inert and useless in chemistry, gold complexes have proven in fact very powerful and they are attracting much interest. Besides unique carbophilic properties which make gold complexes extremely efficient in π-acid catalysis, 2-electron redox transformations at gold have emerged over the past 10–15 years.^1^ In line with the very high redox potential of the Au(i)/Au(iii) couple, Au(i) complexes are inherently reluctant to 2-electron oxidation into well-defined Au(iii) complexes. Strong oxidants are thus required. Known examples include aqua regia, X_2_ and surrogates, F^+^ equivalents, diazonium and iodonium salts, I(iii) derivatives…^2^ Oxidative addition of S–S, Si–Si, Sn–Sn^3^ and strained C–C^4^ bonds to gold(i) have also been reported, as well as C(sp^2^) and C(sp)–I/Br bonds, most recently.^5^ Depending on the gold complex and the oxidant, the Au(i) → Au(iii) transformation occurs spontaneously or it is promoted by a photoredox catalyst, by UV-vis irradiation or by a bidentate ligand. The ensuing Au(iii) complexes are interesting on their own,^6^ with applications ranging from catalysis, materials science to medicinal chemistry. Au(i)/Au(iii) redox cycles also open new avenues in gold catalysis, and major achievements have been reported recently in cross-coupling and alkene difunctionalization reactions in particular.^1,7^

To develop this chemistry further, it is highly desirable to identify and study new routes to cycle between Au(i) and Au(iii). In this context, we questioned here the possibility to use *o*-benzoquinones to oxidize Au(i) complexes and obtain catecholate Au(iii) complexes. Precedents for Au(iii) catecholate complexes are rare. They mainly derive from *C*,*N*-cyclometallated ligands^8^ and have all been accessed by reacting Au(iii) dihalo complexes with catecholates (ligand exchange). Another well-established route to prepare catecholate complexes consists in the 2-electron oxidation of transition metals by *o*-benzoquinones.^9^ This alternative strategy is known for many metals, from group 6 to group 12 (Mo, Fe, Co, Rh, Ir, Ni, Pd, Pt, Cu, Zn…), but to the best of our knowledge, it is unprecedented with gold.^10,11^

**Chart 1:**
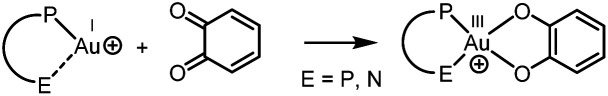
Ligand-enabled 2-electron oxidation of gold(i) complexes by *o*-quinones, as studied in this work.

Here we report that strongly oxidizing *o*-benzoquinones, *i.e. o*-chloranil and its bromo/fluoro congeners, do react with Au(i) complexes to give the corresponding Au(iii) catecholate complexes (Chart 1). P^P chelating or P^N hemilabile ligands enable the reaction to proceed. The structure of the obtained Au(iii) catecholate complexes has been thoroughly analyzed by NMR, X-ray diffraction and absorption (XRD and XAS) means. Reactivity studies substantiate the reversibility of the transformation. DFT calculations shed light on the 2-electron transfer from gold to the *o*-quinone.

## Results and discussion

**Scheme 1:**
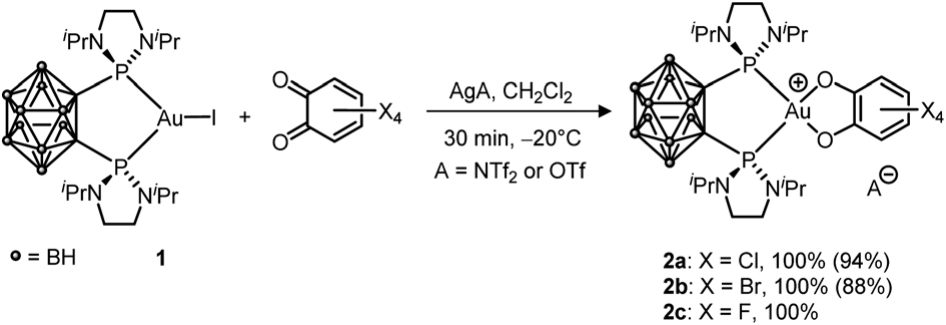
Oxidation of the (P^P)-chelated Au(i) complex 1 with tetrahalo *o*-benzoquinones (isolated yields in parentheses).

First, the oxidation of LAuCl complexes featuring simple phosphines and NHC ligands (PPh_3_ and IPr = 1,3-bis(2,6-diisopropylphenyl)imidazol-2-ylidene) ligands by *o*-benzoquinones was studied. All our attempts remained unsuccessful, whatever the *o*-benzoquinone (Cl_4_, F_4_ or 3,5-^*t*^Bu_2_-substituted) and the conditions (with/without a silver salt present). No reaction was observed within hours at room temperature and forcing the conditions resulted only in degradation, the corresponding L_2_Au^+^ complex being systematically detected within the obtained reaction mixtures. To promote the oxidation of Au(i) and stabilize the ensuing Au(iii) species, we then turned to *o*-carboranyl diphosphines. We wondered if the bending strategy we initially developed to trigger oxidative addition to gold^4*b*,5*a*^ could also work here. Gratifyingly, following halogen abstraction with AgNTf_2_ or AgOTf, the tetrachloro *o*-benzoquinone (*o*-chloranil) was found to rapidly and cleanly react with the *o*-carboranyl diphosphine complex 1 to give 2a (Scheme 1).^12^ The related tetrabromo and tetrafluoro *o*-benzoquinones behave similarly, but no reaction occurred with the less oxidizing parent and 3,5-^*t*^Bu_2_-substituted *o*-benzoquinones. The (P^P) Au (catecholate) complexes 2a–c were characterized by multi-nuclear NMR spectroscopy. They all display a^31^P NMR signal at about *δ* 88–89 ppm. A good reporter for the oxidation of gold from Au(i) to Au(iii) is the methyl ^1^H NMR signal for the ^*i*^Pr group at N. As for the oxidative addition products of aryl iodides,^5*a*^ it is found at *δ* ∼ 1.45 ppm for complexes 2a–c*vs.* 1.23 ppm for 1.

**Fig. 1:**
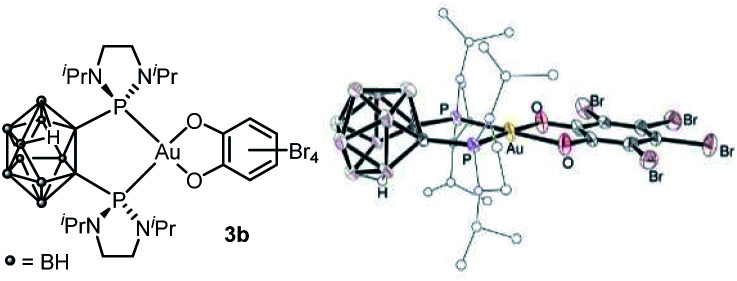
Molecular structure of the *nido*-P^P Au(iii) catecholate complex 3b (For sake of clarity, the substituents at phosphorus are simplified and the hydrogen atoms are omitted, except the bridging one within the nido-carborane cage).

Attempts to grow crystals suitable for X-ray diffraction analysis invariably led to some decomposition and only the corresponding neutral B-decapped complex 3b featuring a *nido*-carborane cage could be structurally authenticated (Fig. 1 and Table 1). The gold center is tetracoordinate, with chelating P^P and O^O ligands. It sits in a quasi-ideal square-planar environment (*τ*_4_ = 0.07).^13^ The C–O and C–C bond lengths (1.33–1.34 and 1.406–1.143 Å, respectively) fall in the typical range for catecholate complexes^14^ and unambiguously report for the 2-electron transfer from gold to the *o*-quinone (the metrical oxidation state (MOS), as introduced by Brown,^14*b*^ is actually – 1.892). A few crystals of 3b were redissolved in *d*_2_-DCM and analyzed by NMR. In line with B-decapping, a broad ^1^H NMR signal was observed at *δ* −2.7 ppm for the BHB bridge, while the ^11^B NMR spectrum displayed five signals between *δ* −8.6 and −32.8 ppm.

**Table 1:** Key geometric features for the (P^P)- and (P^N)-chelated Au(iii) catecholate complexes 3b (the asymmetric unit contains two molecules of very similar structures, only one set of data are reported) and 5a–c as obtained from X-ray diffraction analyses. Bond lengths in Å, bond angles in °

	3b	5a	5b	5c
*τ*_4_^13^	0.07	0.03	0.04	0.04
AuP	2.270 (2)	2.279 (1)	2.278 (1)	2.274 (1)
AuP/AuN	2.272 (2)	2.065 (2)	2.066 (3)	2.053 (4)
PAuP/PAuN	90.03 (5)	87.71 (5)	87.60 (7)	87.77 (12)
CO	1.334 (7)	1.363 (3)	1.341 (4)	1.367 (6)
	1.337 (7)	1.350 (3)	1.351 (4)	1.350 (6)
CC	1.405 (9)	1.394 (3)	1.398 (5)	1.408 (6)
MOS^14*b*^	−1.89 (6)	−2.03 (7)	−1.89 (10)	−1.99 (10)

The *o*-carborane framework survives the oxidation of gold by the *o*-quinones to give complexes 3a–c, but it slowly degrades afterwards from a *closo* to a *nido* form.^15^ With the aim to increase the stability of the Au(iii) catecholate complex and generalize the approach of ligand-enabled oxidation of Au(i) with *o*-quinones, we then moved to the hemilabile P^N ligand MeDalphos we showed to be very efficient to promote the oxidative addition of aryl/alkynyl/vinyl-iodides as well as aryl-bromides to gold.^5*b*–*d*^

**Scheme 2:**
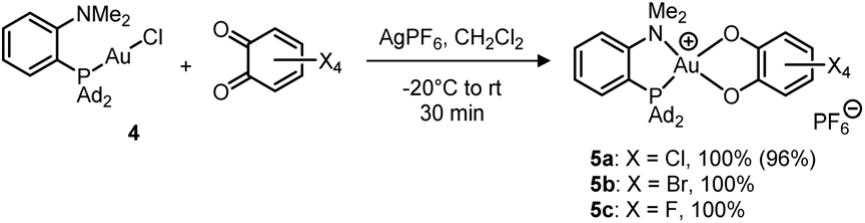
Oxidation of the (P^N)-ligated Au(i) complex 4 with tetrahalo *o*-benzoquinones (isolated yields in parentheses).

Treatment of (MeDalphos)AuCl with *o*-chloranil in the presence of a chloride abstractor (AgOTf, AgPF_6_, AgSbF_6_, NaBAr^F^_24_ with Ar^F^ = 3,5-(CF_3_)_2_C_6_H_3_) was found to immediately and quantitatively give the corresponding Au(iii) catecholate complex 5a (Scheme 2).^12^ The reaction proceeds similarly with the related tetrabromo and tetrafluoro *o*-benzoquinones to give the corresponding Au(iii) catecholate complexes 5b,c. Again, no reaction was observed with the less oxidizing parent and 3,5-^*t*^Bu_2_-substituted *o*-benzoquinones. Diagnostic of the Au(i) to Au(iii) oxidation and P^N chelation in 5a–c are the low field shifts of the ^31^P (*δ* ∼ 88 ppm), ^1^H (NMe_2_, *δ* ∼ 4.0 ppm) and ^13^C(NMe_2_, *δ* ∼ 60 ppm) NMR resonances, as well as the ^15^N chemical shift (*δ* 68.5 ppm for 5a, as determined by an HSQC ^15^N–^1^H experiment). Very similar data were observed for the related [(P^N)AuCl_2_]SbF_6_ complex (*δ*^31^P: 109.8 ppm; ^1^H: 3.92 ppm; ^13^C: 59.1 ppm; ^15^N: 82.3 ppm).^16^

**Fig. 2:**
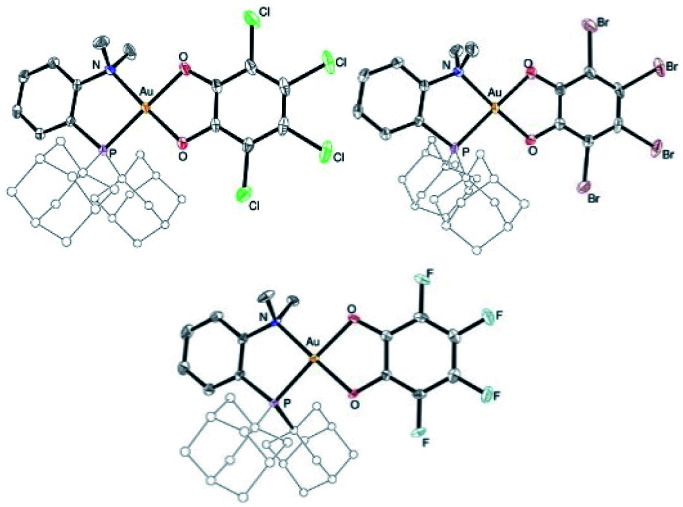
Molecular structures of the P^N Au(iii) catecholate complexes 5a–c (for sake of clarity, the substituents at phosphorus are simplified and the hydrogen atoms are omitted).

The higher stability of the P^N-ligated complexes 5a–c enabled to grow crystals and carry out X-ray diffraction analyses (Fig. 2 and Table 1). The molecular structures of 5a–c are very similar. The three complexes adopt discrete ion-pair structures with tetracoordinate square-planar Au centers (*τ*_4_ = 0.03).^13^ The short N–Au distances (2.05–2.07 (2) Å) and small PAuN bite angles (87.6–87.8°) testify to the strong coordination of N to gold and thus to the chelating behavior of the P^N ligand. Oxidation from Au(i) to Au(iii) and concomitant *o*-quinone to catecholate reduction is again clearly apparent from the C–O/C–C bond lengths (1.34–1.37/1.39–1.41 Å, respectively) and MOS values (from −1.887 to −2.029).^14^

**Fig. 3:**
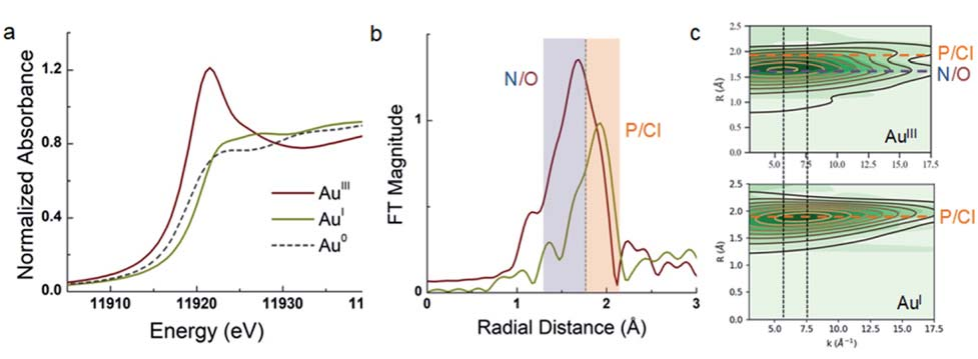
(a) Comparison of the Au L_3_-edge profiles for 5a (red), (MeDalphos)AuCl (yellow) and Au foil (grey), having +3, +1, and 0 oxidation states, respectively. (b) Fourier Transformed (FT) spectra of 5a (red) and (MeDalphos)AuCl (yellow) with *k*-range 3–17.5 Å^−1^, Hannings window dk = 1. (c) Cauchy wavelet transforms of data (green density) and fit (contour line) showing signal dependence on both r-space and *k*-space.

The assignment of 5a as a Au(iii) catecholate complex was further supported by X-ray absorption spectroscopy (Fig. 3). The XANES (X-ray Absorption Near-Edge Structure) profile at the Au L_3_-edge is dominated by 2p → 5d dipole allowed transitions, and is particularly sensitive to metal oxidation state. Gold in Au foil (Au(0)) and (MeDalphos)AuCl (Au(i), d^10^ complex) do not exhibit intense whiteline features due to the lack of vacant d acceptor orbitals. Nevertheless, the rising edge for the Au(i) species at 11 920.7 eV is higher in energy relative to Au^0^ at 11.919 eV reflecting the change in oxidation state. In contrast, 5a shows an intense whiteline peak with a maximum centered at 11 921.5 eV consistent with a Au(iii) d^8^ gold center.^17^ Furthermore, EXAFS (extended X-ray absorption fine structure) analysis of 5a clearly describes a tetracoordinate Au center with 1 P/Cl scattering atom at 2.27 Å,^3^ N/O atoms distributed at 1.96 Å and 2.05 Å. For comparison, (MeDalphos)AuCl has a much lower intensity in the Fourier transformed spectra and only exhibits two Au–P/Cl scattering interactions at ∼2.30 Å. Both EXAFS models are consistent with crystallographic data and theoretical models.

**Scheme 3:**
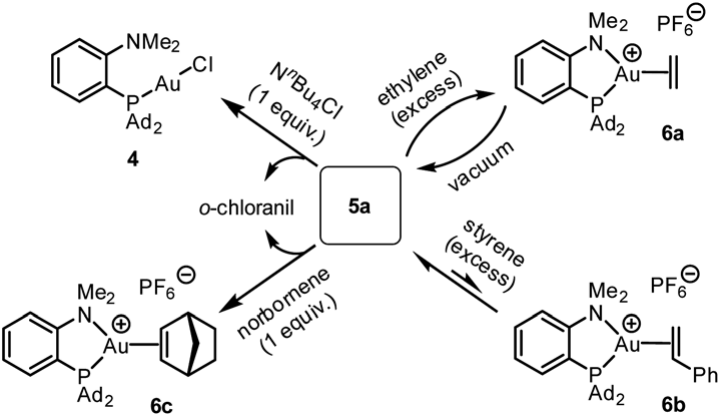
Reactivity of the (P^N)Au(iii) catecholate complex 5a towards ammonium chloride and alkenes.

**Fig. 4:**
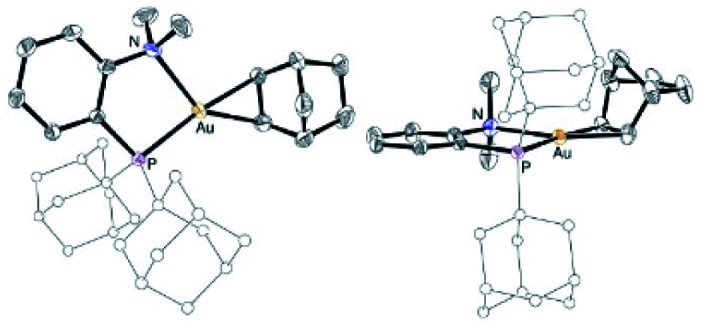
Molecular structure of the P^N Au(i) norbornene complex 6c, top view (left) and side view (right) (for sake of clarity, the substituents at phosphorus are simplified and the hydrogen atoms are omitted).

The reactivity of the (P^N)Au (tetrachloro-catecholate) complex 5a was then interrogated (Scheme 3). Upon treatment with 1 equivalent of tetra-*n*-butylammonium chloride, 5a was immediately and quantitatively converted back into the (MeDalphos)AuCl complex 4. With ethylene, a large excess was needed to drive the reaction to completion. Under vacuum, the resulting ethylene complex 6a^18^ gave back 5a, demonstrating the possibility to easily cycle between Au(i) π-alkene and Au(iii) catecholate complexes. The addition of 5 equivalents of styrene to 5a resulted in an equilibrium with partial formation of the corresponding Au(i) π-complex 6b (∼25 : 75 ratio based on ^31^P NMR spectroscopy).^18^ With norbornene, the reaction is shifted forward and the Au(iii) catecholate complex was quantitatively transformed into the new Au(i) π-complex 6c with only one equivalent of alkene. As unambiguously established by X-ray diffraction analysis (Fig. 4), the C=C double bond is side-on coordinated to gold *via* its *exo* face and the P^N ligand is strongly chelating, inducing significant Au to alkene back-donation.^18^ Of note, the reduction of gold from 5a to complexes 4 and 6c was accompanied by the oxidation of the catecholate moiety and *o*-chloranil was released, as apparent from ^13^C NMR spectroscopy.^12^ The oxidation of gold by *o*-quinones is thus reversible and it can be shifted backward by adding a competing ligand for gold(i), such as chloride or alkenes.

**Fig. 5:**
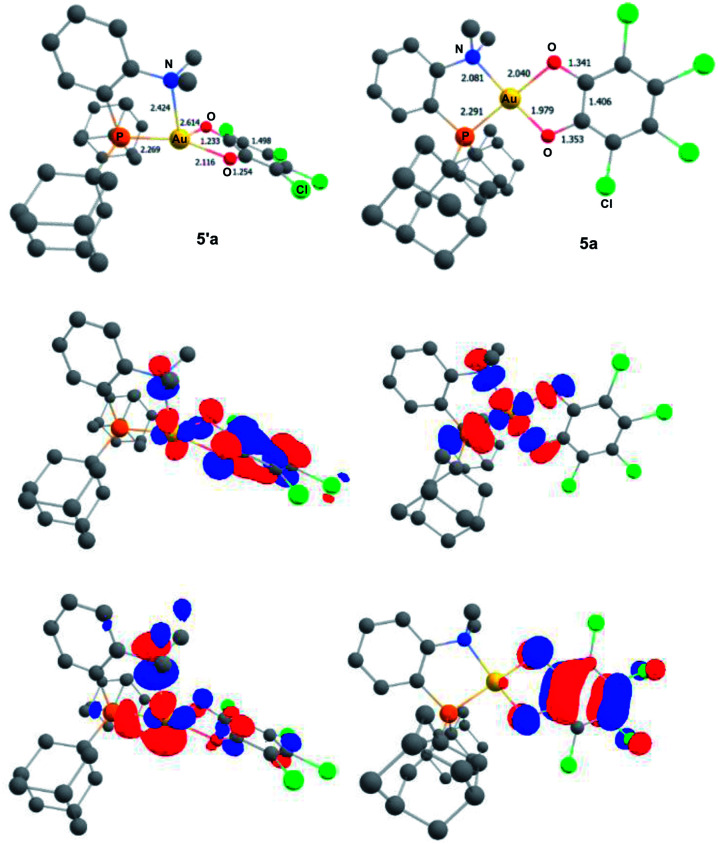
Optimized geometries of the Au(i) *o*-benzoquinone (5′a, left) and Au(iii) catecholate (5a, right) valence isomers of the (P^N)Au(O^O)Cl_4_^+^ complex, computed at the PCM(DCM)-B3PW91-D3(BJ)/SDD + f (Au), 6-31G** (C, H, N, O, P, Cl) level of theory. Distances in Å. Plot of the frontier orbitals with cutoff: 0.05. Hydrogen atoms have been omitted for clarity.

To gain more insight into the structure and stability of the Au(iii) catecholate complexes, DFT calculations were performed at the B3PW91-D3(BJ)/SDD + f (Au), 6-31G** (H, C, N, O, P, Cl) level of theory. The counter-anion was not included, but solvent (PCM–DCM) and dispersion effects (Grimme's D3BJ corrections) were taken into account. First, complexes featuring the actual P^N ligand MeDalphos were investigated, to compare with the experimental results. Both *o*-chloranil and the parent *o*-benzoquinone were considered. In both cases, two energy *minima* were located on the potential energy surface (Fig. 5 for *o*-chloranil, Fig. S44† for *o*-benzoquinone). The ground state structure corresponds to the square-planar Au(iii) form, as obtained experimentally upon gold oxidation with *o*-chloranil. The optimized geometry reproduces nicely the solid-state structure determined crystallographically. The other form (5′a) is a Au(i) complex with the *o*-benzoquinone unsymmetrically coordinated (Au–O distances of 2.116 and 2.614 Å) and seesaw-type geometry (bond angles: PAuO 159.73 and 114.56°, PAuN 82.67°).^19^ The ^31^P, ^1^H and ^13^C NMR chemical shifts computed for the Au(iii) form 5a match nicely those observed experimentally, but differ noticeably from those of the corresponding Au(i) form 5′a (Table S2†), corroborating our structural assignment.

**Chart 2:**
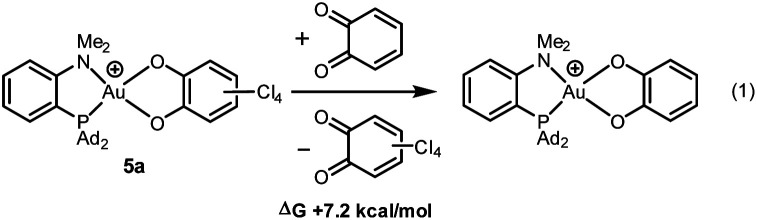
Catechol/*o*-quinone exchange (isodesmic reaction) considered to compare the reactivity of the parent and tetrachloro *o*-quinones towards Au(i) oxidation.

With *o*-chloranil, the Au(iii) catecholate complex 5a is 10.8 kcal mol^−1^ lower in energy than the corresponding Au(i) valence isomer 5′a. This energy gap is reduced to 1.1 kcal mol^−1^ for the parent *o*-benzoquinone, the Au(i) form being only slightly above the Au(iii) form in this case. To compare the propensity of the two *o*-quinones to react with the (P^N)Au^+^ fragment, the thermodynamic balance of the isodesmic reaction (1) was computed (Chart 2). It was found uphill in energy by 7.2 kcal mol^−1^. This is consistent with experimental observations. With less oxidizing *o*-quinones, no reaction occurs.

Besides the geometric features, the changes in the redox states of the Au center and O^O ligand between 5′a and 5a is clearly apparent from the respective frontier orbitals (Fig. 5). For the Au(i) valence isomer, the HOMO is centered on Au (with some parentage of the N lone pair) and the LUMO corresponds to the π* of the *o*-quinone moiety, while for the Au(iii) form, the HOMO is centered on the catecholate ligand and the LUMO is centered on gold (in-plane 5d orbital in anti-bonding interaction with O, P and N).

**Fig. 6:**
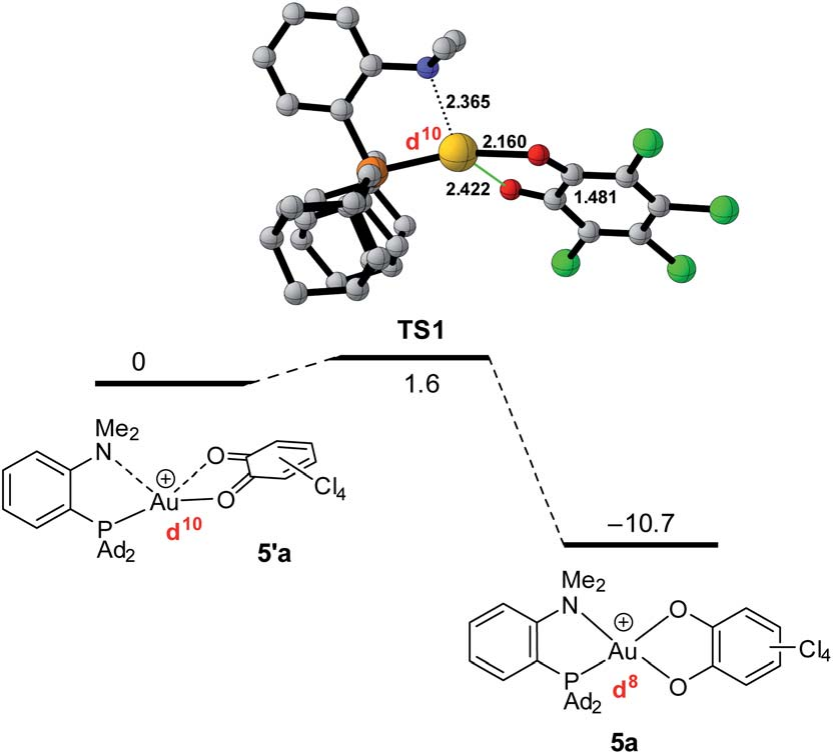
Conversion of the Au(i) *o*-quinone valence isomer 5′a into the Au(iii) catecholate complex 5a, computed at the PCM(DCM)-B3PW91-D3(BJ)/SDD + f (Au),6-31G**(C, H, N, O, P, Cl) level of theory.

**Fig. 7:**
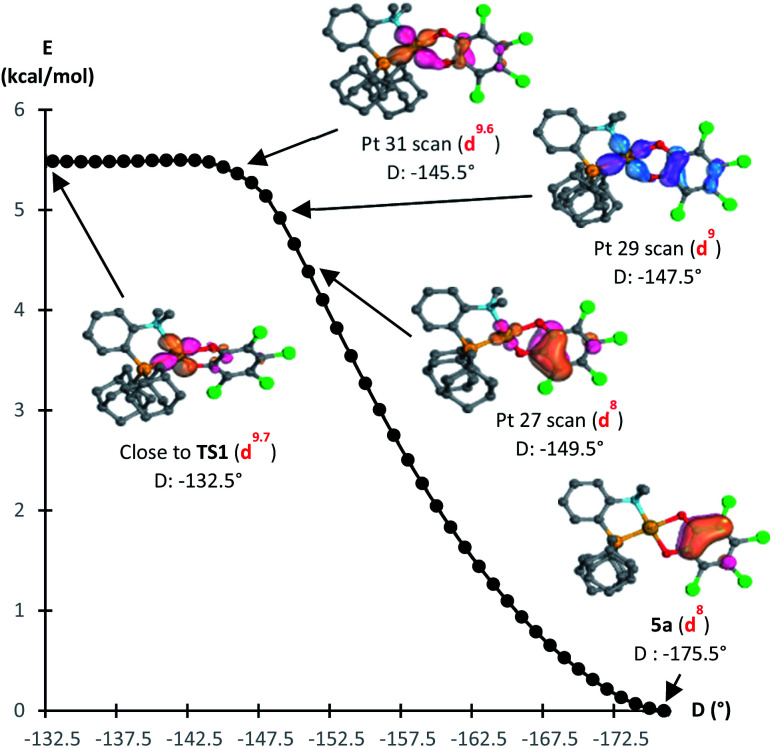
Reaction path from TS1 to 5a by integrating the intrinsic reaction coordinate (D = bond dihedral angle of O_*cis*-to-P_AuPC_Ph_ in °), computed at the B3PW91-D3(BJ)/SDD + f (Au), 6-31G** (C, H, N, O, P, Cl) level of theory. IBO orbitals of the Au to *o*-quinone electron transfer along the reaction coordinate from TS1 to 5a, showing the change from an in-plane 5d (Au) to a π (catecholate) orbital. Electron configuration at Au from NBO calculations.

The formation of the Au(iii) catecholate complex 5a from the corresponding Au(i) *o*-quinone 5′a was then investigated (Fig. 6). 2-Electron transfer from gold to the *o*-quinone was found to be about barrierless (1.6 kcal mol^−1^) in line with the facile and instantaneous formation of 5a. The corresponding transition state (TS1) connecting 5′a to 5a is actually very early. It resembles the Au(i) *o*-quinone form and retains d^10^ configuration. The Au to *o*-quinone electron transfer occurs later, when falling down to the Au(iii) catecholate form, as apparent from IBO analysis.^12^ From TS1 to 5a, close to the transition state, one of the in-plane 5d (Au) orbital evolves into a π(catecholate) orbital (Fig. 7), while the two π_CO_(o-quinone) orbitals turn into n_O_^π^ (catecholate) orbitals (Fig. S46†). In the mean time, the electron configuration of gold changes from d^10^ to d^8^ according to the occupancy of the 5d (Au) orbitals (NBO calculations).

**Fig. 8:**
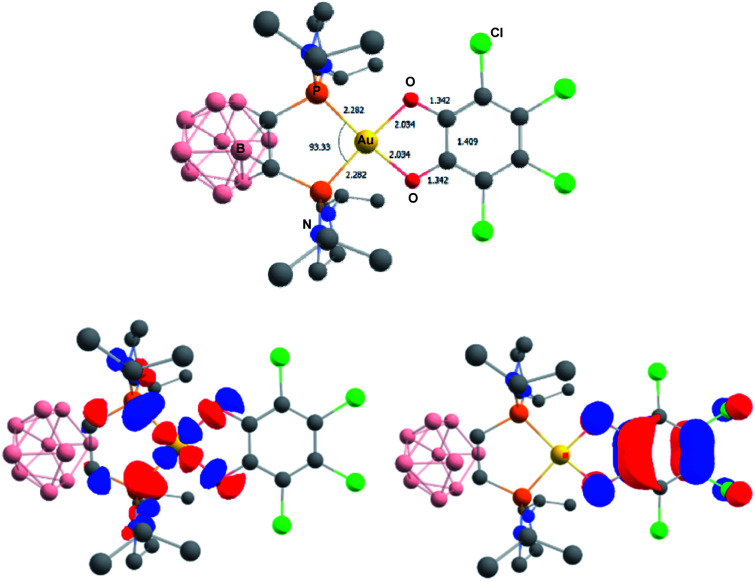
Optimized geometry of the Au(iii) catecholate complex 2a, computed at the PCM(DCM)-B3PW91-D3(BJ)/SDD + f (Au), 6-31G** (H, B, C, N, O, Cl, P) level of theory. Distances in Å, angles in °. Plot of the frontier orbitals with cutoff: 0.05. Hydrogen atoms have been omitted for clarity.

The related gold complexes featuring the chelating *o*-carboranyl diphosphine were also computed. At the same level of theory, taking into account dispersion and solvent effects, only the Au(iii) catecholate forms could be located as energy *minima* on the potential energy surface (Fig. 8). Au(i) *o*-quinone structures could be found as a transition state on PES for the parent *o*-benzoquinone and only by imposing geometric constraints (torsion of the *o*-quinone moiety with respect to the (P^P)Au coordination plane) for the chlorinated derivative. They sit 6.4 and 14.2 kcal mol^−1^ above the Au(iii) catecholate forms for the parent *o*-benzoquinone and *o*-chloranil, respectively.^20^ The optimized geometry and frontier orbitals of the experimentally prepared Au(iii) catecholate complex 2a are displayed in Fig. 8. The P^P ligand is chelating gold which sits in a quasi-perfect square-planar environment (PAuP bite angle = 93.3°, *τ*_4_ = 0.07).^13^ The C–O and C–C bond lengths (1.343 and 1.410 Å, respectively) are diagnostic for the reduction of *o*-chloranil into the corresponding catecholate. Moreover, the HOMO/LUMO very much resemble those of the related P^N-ligated complex 5a.

## Conclusions

Chelating P^P and hemilabile P^N ligands were found to promote the reaction of Au(i) complexes with *o*-benzoquinones to give Au(iii) catecholate complexes. While single crystals suitable for X-ray diffraction analysis could not be obtained with the *o*-carboranyl diphosphine ligand due to slow B-decapping, the P^N-ligand MeDalphos provided highly stable and crystalline complexes. NMR spectroscopy, X-ray diffraction and X-ray absorption spectroscopy unambiguously establish the 2-electron process, *i.e.* oxidation of gold and reduction of the *o*-quinone. The reaction requires strongly oxidizing *o*-quinones such as *o*-chloranil and the related tetrabromo/tetrafluoro *o*-benzoquinones. It is reversible, as substantiated by reactivity studies. The addition of competing ligands such as chloride and alkenes gives back Au(i) complexes with concomitant release of the *o*-quinone. The structure and stability of the Au(i) *o*-quinone and Au(iii) catecholate forms were thoroughly compared thanks to DFT calculations. 2-Electron transfer from gold to the *o*-quinone was found to be indeed favored by the strongly oxidizing *o*-chloranil and to occur at a late stage, after the transition state connecting the Au(i) *o*-quinone and Au(iii) catecholate forms.

Complexes 2a–c and 5a–c represent a new type of Au(iii) catecholate complexes, deriving from P-containing ligands, and the ligand-enabled oxidation of Au(i) precursors with *o*-quinones oxidation stands as a new synthetic route. Future work will aim to extend the approach to other chelating/hemilabile ligands and increase further the chemical diversity of Au(iii) catecholate complexes. Such complexes are expected to exhibit versatile chemical, redox, photophysical properties that will be thoroughly studied and tuned by ligand design. On mid term, Au(iii) catecholate complexes may open new avenues in materials science (luminescence, optoelectronic applications), medicinal chemistry (cytotoxicity) and catalysis. Au(iii) complexes,^7^ including complexes deriving from O,O-ligands (catechols, β-diketones, bis-naphthols, bis-phenols),^8*a*,21^ have been recently shown to be quite promising in these areas and they attract increasing interest.

## Data availability

The ESI† contains the experimental procedures, the analytical data for the obtained products, the NMR spectra, the XRD and XAS data, the computational details and *Z*-matrices for the optimized structures.

## Conflicts of interest

There are no conflicts to declare.

## Author contributions

G. S. and D. B. designed the experiments. G. S. and J. B. conducted the experiments. G. S. processed and interpreted the analytical data. V. M.-D. and S. M.-L. performed and interpreted the XAS and XRD analyses, respectively. Y. G.-R. and K. M. performed and analyzed the DFT calculations. The manuscript was written and reviewed by all authors. D. B. designed and directed the project.

## Supplementary Material

SC-013-D2SC03724F-s001

SC-013-D2SC03724F-s002
