# Supplementary material for: Ligand-enabled oxidation of gold(i) complexes with o-quinones
Source: Chem Sci. 2022 Aug 5;13(35):10499–505. doi: 10.1039/d2sc03724f (PMC9473537; doi:10.1039/d2sc03724f)
Supplement: SC-013-D2SC03724F-s001 [file SC-013-D2SC03724F-s001.pdf]

Electronic Supplementary Material (ESI) for Chemical Science.
This journal is © The Royal Society of Chemistry 2022

Supporting Information

1. Materials and Methods S2
2. Synthesis of the gold(III) catecholate complexes S3
3. Reactions of **5a** S22
4. Crystallographic data S31
5. X-ray absorption analyses S33
6. Computational details S36
7. Z-matrices and energies in au S51
8. References S61

1. Materials and Methods

All reactions and manipulations were carried out under an atmosphere of dry argon using standard Schlenk techniques or in a glovebox under an inert atmosphere. Dry, oxygen-free solvents were employed. Solution ^{1}H, ^{13}C, ^{31}P, ^{19}F and ^{15}N NMR spectra were recorded on Bruker Avance 300, 400 or 500 spectrometers at 298 K. Chemical shifts (δ) are expressed with a positive sign, in parts per million. ^{1}H and ^{13}C chemical shifts reported are referenced internally to residual protio (^{1}H) or deutero (^{13}C) solvent, while ^{31}P, ^{19}F and ^{15}N chemical shifts are relative to 85% H_3PO_4, $CFCl_3$ and liquid ammonia respectively. The following abbreviations and their combination are used: br, broad; s, singlet; d, doublet; t, triplet; m, multiplet; sept, septuplet. The ^{1}H and ^{13}C resonance signals were attributed by means of 2D HSQC, HMBC experiments. Mass spectra were recorded on a Waters UPLC Xevo G2 Q TOF apparatus. Elemental analyses were performed by the in-house service at the *Laboratoire de Chimie de Coordination* (205, Route de Narbonne, 31077, Toulouse, France) on a PerkinElmer 2400 Series II system. **MeDalPhosAuCl** (**4**), ***o*-chloranil** and ***o*-bromanil** were purchased from commercial suppliers and used without further purification. ***closo*-DPCb** was synthesized according to a previously described protocol.[1] ***closo*-DPCbAuI** (**1**) was synthesized based on a previously described protocol.[2] ***o*-fluroanil** was synthesized according to a previously described protocol.[3]

2. Synthesis of the gold(III) catecholate complexes

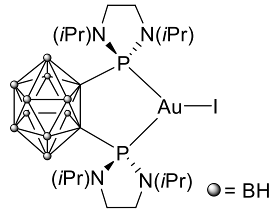

***closo*-DPCbAuI** (**1**). To a suspension of **AuI** (358 mg, 1.105 mmol, 1 equiv.) in CH_2Cl_2 (20 mL) a solution of ***closo*-DPCb** (540 mg, 1.105 mmol, 1 equiv.) in CH_2Cl_2 (20 mL) was added at -20°C. Then the temperature was raised to r.t. After 30 minutes at r.t., the complete formation of **1** was observed by ^{31}P NMR (140.1 ppm). The solvent was removed and the residue was crystallized from a saturated solution in CH_2Cl_2 by toluene layering at -20°C, to afford the title complex (815 mg, 91%) as a pale yellow solid. m.p.: 258 °C; **^{1}H{^{31}P} NMR** (300.1 MHz, CD_2Cl_2): δ 3.66 (sept, 4H, $^{3}J_{HH}$ = 6.6 Hz, C*H*$_{iPr}$), 3.38-3.18 (m, 8H, N(C*H*$_2$)$_2$N), 3.2-1.4 (br, ~10H, BH), 1.23 (t, 24H, $^{3}J_{HH}$ = 6.6 Hz, C*H*$_3$); **^{13}C{^{1}H}{^{31}P} NMR** (125.8 MHz, CD_2Cl_2): δ 95.9 (s, *C*-Cb), 50.2 (s, *C*H(CH$_3$)$_2$), 43.2 (s, N(*C*H$_2$)$_2$N), 21.8 (s, CH(*C*H$_3$)$_2$), 20.9 (s, CH(*C*H$_3$)$_2$); **^{31}P{^{1}H} NMR** (121.5 MHz, CD_2Cl_2); δ 140.9 (s); **^{11}B NMR** (96.3 MHz, CD_2Cl_2): δ -3.0 (s), -4.5 (s), -10.7 (s), -15.4 (s); HRMS (ESI+) calcd. for $[M+H]^+$ = $C_{18}H_{47}AuB_{10}IN_4P_2^+$: 813.2998, found 813.3004.

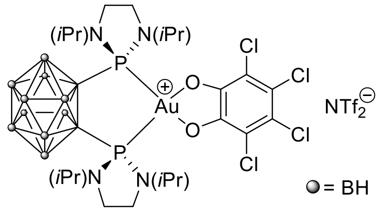

***closo*-DPCbAu(catCl$_4$)NTf$_2$** (**2a**). To a suspension of **AgNTf$_2$** (57 mg, 0.148 mmol, 1.2 equiv.) in CH_2Cl_2 (1 mL) a solution of **1** (100 mg, 0.123 mmol, 1 equiv.) in CH_2Cl_2 (5 mL) was added at -20°C. After 30 minutes at -20°C, the complete formation of ***closo*-DPCbAuNTf$_2$** was observed by ^{31}P{^{1}H} NMR (138.3 ppm). Then this suspension was cannula filtered onto solid ***o*-chloranil** (45 mg, 0.185 mmol, 1.5 equiv.) at -20 °C. After 30 minutes at -20°C, ^{31}P{^{1}H} NMR check (89.1 ppm) showed the formation of the desired product. The solvent was removed and the resulting solid was washed with pentane (4 x 5 mL) and dried under vacuum to afford **2a** (140 mg, 94%) as a grey-brown solid. m.p.: ~200 °C decomp.; **^{1}H NMR** (300.1 MHz, CD_2Cl_2): δ 3.85-3.69 (m, 8H, C*H*$_{iPr}$ & N(C*H*$_2$)$_2$N), 3.55-3.48 (m, 4H, N(C*H*$_2$)$_2$N), 3.2-1.9 (br, ~10H, BH), 1.45 (d, $^{3}J_{HH}$ = 6.6 Hz, 24H, CH(C*H*$_3$)$_2$); **^{13}C{^{1}H}{^{31}P} NMR** (125.8 MHz, CD_2Cl_2): δ 153.9 (s, C_{cat}), 142.3 (s, C_{cat}), 123.9 (s, C_{cat}), 122.6 (s, C_{cat}), 120.3 (q, $^{1}J_{FC}$ = 323.0 Hz, C_{NTf2}), 119.2 (s, C_{cat}), 118.6 (s, C_{cat}), 79.1 (*C*-Cb), 51.8 (*C*H$_{iPr}$), 43.2 (N(*C*H$_2$)$_2$N), 21.2 (CH(*C*H$_3$)$_2$), 20.7 (CH(*C*H$_3$)$_2$); **^{31}P{^{1}H} NMR** (121.5 MHz, CD_2Cl_2) δ 89.1 (s); **^{19}F{^{13}C} NMR** (282.4 MHz, CD_2Cl_2): δ -78.5 (s, C*F*$_3$-NTf$_2$); **^{11}B NMR** (96.3 MHz, CD_2Cl_2): δ -1.2 (s), -11.2 (s), -14.5 (s); HRMS (ESI+) calcd. for $[M]^+$ = $C_{24}H_{46}AuB_{10}Cl_4N_4O_2P_2^+$: 931.2499, found 931.2520; calcd. for $[M-B+H]^+$ = $C_{24}H_{47}AuB_9Cl_4N_4O_2P_2^+$: 921.2469, found 921.2504; HRMS (ESI-) calcd. for $[NTf_2]^-$ = $C_2F_6NO_4S_2$: 279.9173, found 279.9170.

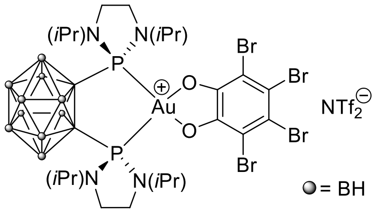

***closo*-DPCbAu(catBr$_4$)NTf$_2$** (**2b**). To a suspension of **AgNTf$_2$** (52.5 mg, 0.135 mmol, 1.1 equiv.) in CH_2Cl_2 (1 mL) a solution of **1** (100 mg, 0.123 mmol, 1 equiv.) in CH_2Cl_2 (5 mL) was added at -20°C. After 30 minutes at -20°C, the complete formation of ***closo*-DPCbAuNTf$_2$** was observed by ^{31}P{^{1}H} NMR (138.3 ppm). Then this suspension was cannula filtered onto solid ***o*-bromanil** (52 mg, 0.123 mmol, 1 equiv.) at -20 °C. After 30 minutes at -20°C, ^{31}P{^{1}H} NMR check (88.6 ppm) showed the formation of the desired product. The solvent was removed and the resulting solid was washed with pentane (4 x 5 mL) and dried under vacuum to afford the **2b** (150 mg, 88%) as a grey solid. m.p.: 216-218 °C; **^{1}H NMR** (300.1 MHz, CD_2Cl_2): δ 3.86-3.69 (m, 8H, CH(C*H*$_3$)$_2$ & N(C*H*$_2$)$_2$N), 3.58-3.46 (m, 4H, N(C*H*$_2$)$_2$N), 3.3-1.7 (br, ~10H,

BH), 1.45 (d, $^3J_{HH}$ = 6.5 Hz, 24H, CH(CH_3)$_2$); **^{13}C{^{1}H} NMR** (125.8 MHz, CD_2Cl_2): δ 154.3 (s, C_{cat}), 149.6 (s, C_{cat}), 119.9 (q, $^1J_{FC}$ = 321.0 Hz, C_{NTf2}), 117.0 (s, C_{cat}), 115.7 (s, C_{cat}), 113.0 (s, C_{cat}), 105.8 (s, C_{cat}), 79.1 (s, C_{DPCb}), 50.8 (s, CH_{iPr}), 43.2 (s, N(CH_2)$_2$N), 21.3 (s, CH_{3iPr}), 20.8 (s, CH_{3iPr}); **^{31}P{^{1}H} NMR** (121.5 MHz, CD_2Cl_2) δ 89.2 (s); **^{19}F{^{13}C} NMR** (282.4 MHz, CD_2Cl_2): δ -78.6 (s, CF_3-NTf_2); **^{11}B NMR** (96.3 MHz, CD_2Cl_2): δ -1.8 (s), -11.7 (s), -16.1 (s); HRMS (ESI+) calcd. for $[M]^+$ = $C_{24}H_{46}AuB_{10}Br_4N_4O_2P_2^+$: 1109.0468, found 1109.0476; HRMS (ESI-) calcd. for $[NTf_2]^-$: 279.9173, found 279.9179; **Elemental Analysis**: calcd. for $C_{26}H_{46}AuB_{10}Br_4F_6N_5O_6P_2S_2$: C 22.48, H 3.34, N 5.04 found: C 22.70, H 3.27, N 5.05.

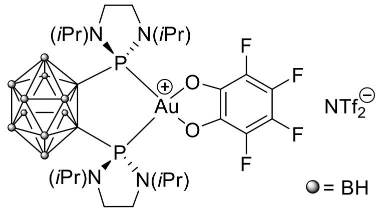

***closo*-DPCbAu(catF$_4$)NTf$_2$** (**2c**). To a suspension of **AgNTf$_2$** (4.3 mg, 0.01 mmol, 1 equiv.) in CD_2Cl_2 (0.1 mL) a solution of **1** (8.1 mg, 0.01 mmol, 1 equiv.) in CD_2Cl_2 (0.4 mL) was added at -20°C. After 30 minutes at -20°C, the suspension was syringe filtered. To this solution, a CD_2Cl_2 solution of ***o*-fluoranil** (10^{-1}M, 100 μL, 1 equiv.) was added at -20 °C. After 30 minutes at -20°C, the solution was analyzed by multinuclear NMR. The title product was not isolated. It was formed in 100% NMR yield. **^{1}H NMR** (300.1 MHz, CD_2Cl_2): δ 3.88-3.67 (m, 8H), 3.59-3.44 (m, 4H), 3.2-1.9 (br, ~10H, BH); 1.45 (dd, $^3J_{HH}$ = 6.6 Hz, $^4J_{HP}$ = 1.5 Hz, 24H, CH(CH_3)$_2$); **^{31}P{^{1}H} NMR** (121.5 MHz, CD_2Cl_2) δ 87.9 (s); **^{19}F{^{1}H} NMR** (282.4 MHz, CD_2Cl_2): δ -79.0 (s, 12H, CF_3-NTf_2); -168.1 (dd, $^3J_{FF}$ = 17.0 Hz, $^4J_{FF}$ = 11.9 Hz, 4H, *F*cat), -174.9 (dd, $^3J_{FF}$ = 17.0 Hz, $^4J_{FF}$ = 11.9 Hz, 4H, *F*cat).

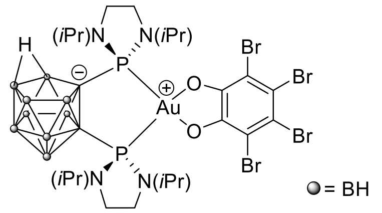

***nido*-DPCbAu(catBr$_4$)** (**3b**). From the crystallization experiments, where the **3b** was identified unambigously by XRD analysis, some of the crystalline material was resolubilized in CD_2Cl_2 and characterized by multinuclear NMR. m.p.: ~92 °C, decomp.; **^{1}H NMR** (300.1 MHz, CD_2Cl_2): δ 3.80-3.31 (m, 12H, CH(CH_3)$_2$ & N(CH_2)$_2$N), 1.46 (d, $^3J_{HH}$ = 6.6 Hz, 6H, CH(CH_3)$_2$), 1.36-1.25 (m, 18H, CH(CH_3)$_2$), -2.4-3 (bs, BHB); **^{31}P{^{1}H} NMR** (121.5 MHz, CD_2Cl_2) δ 106.3 (s); HRMS (ESI+) calcd. for $[M+H]^+$ = $C_{24}H_{47}AuB_9Br_4N_4O_2P_2^+$: 1099.0448, found 1099.0446; HRMS (ESI-) calcd. for $[M-H]^-$ = $C_{24}H_{45}AuB_9Br_4N_4O_2P_2^-$: 1097.0292, found 1097.0291. **^{11}B NMR** (96.3 MHz, CD_2Cl_2): δ -8.6 (s), -15.2 (s), -20.3 (s), 26.8 (s), 32.8 (s).

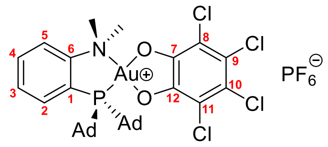

PNAu(catCl$_4$)$^+$PF$_6^-$ (**5a**). To a solution of **AgPF$_6$** (39 mg, 0.153 mmol, 1 equiv.) in CH_2Cl_2 (1 mL) a solution of **4** (100 mg, 0.153 mmol, 1 equiv.) in CH_2Cl_2 (5 mL) was added at -20 °C. Then the temperature was raised to r.t., while a pale yellow precipitate formed gradually. After 30 minutes at r.t. an ^{31}P{^{1}H} NMR check (76.3 ppm) revealed the consumption of the **4**. Then, this suspension was cannula filtered onto solid ***o*-chloranil** (40 mg, 0.161 mmol, 1.05 equiv.) at -20 °C. After 30 minutes at r.t. ^{31}P{^{1}H} NMR check (87.4 ppm) showed the formation of the desired product. The solvent was removed until only about 0.5-1 mL CH_2Cl_2 was left. After addition of pentane (5 mL) a grey precipitate appeared, which was filtered, further washed with pentane (3 x 5 mL) and dried under vacuum to afford the **5a** (148 mg, 96 %) as a grey solid. Single crystals were obtained by slow evaporation of the solvent from the NMR sample. m.p.: ~140 °C decomp.; **^{1}H NMR** (500.1 MHz, acetone-d$_6$): δ 8.47 (ddd, $^3J_{HH}$ = 8.6 Hz, $^4J_{PH}$ = 4.1 Hz, $^4J_{HH}$ = 0.9 Hz, 1H, H_5), 8.34 (td, $^3J_{HH}$ = 8.0, $^3J_{PH}$ = 8.0 Hz, $^4J_{HH}$ = 1.4 Hz, 1H, H_2), 8.13 (dddd, $^3J_{HH}$ = 8.6 Hz, $^3J_{HH}$ = 7.2 Hz, $^5J_{PH}$ = 1.8 Hz, $^4J_{HH}$ = 1.4 Hz, 1H, H_4), 7.94 (dddd, $^3J_{HH}$ = 8.0 Hz, $^3J_{HH}$ =

7.2 Hz, $^4J_{PH}$ = 2.5 Hz, $^4J_{HH}$ = 0.9 Hz, 1H, H_3), 4.11 (s, 6H, NMe_2), 2.79-2.52 (m, 12H, H_{Ad}), 2.16-2.10 (m, 6H, H_{Ad}), 1.94-1.72 (m, 12H, H_{Ad}); **$^{13}C\{^1H\}$ NMR** (125.8 MHz, acetone-d6): δ (d, $^2J_{CP}$ = 6.5 Hz, C_6), 157.8 (d, J_{CP} = 3.6 Hz, C_{cat}), 153.3 (d, J_{CP} = 3.5 Hz, C_{cat}), 138.2 (d, $^4J_{CP}$ = 2.4 Hz, C_4), 136.3 (d, $^2J_{CP}$ = 6.5 Hz, C_2), 133.3 (d, $^3J_{CP}$ = 8.1 Hz, C_3), 125.3 (d, $^3J_{CP}$ = 7.7 Hz, C_5), 122.2 (s, C_{cat}), 120.8 (s, C_{cat}), 118.1 (d, J_{CP} = 4.3 Hz, C_{cat}), 118.0 (d, $^1J_{CP}$ = 49.4 Hz, C_1), 117.7 (d, J_{CP} = 1.0 Hz, C_{cat}), 59.4 (s, $CH_{3\text{-}NMe2}$), 48.5 (d, $^1J_{CP}$ = 13.9 Hz, $C_{q\text{-}Ad}$), 40.0 (s, $CH_{2\text{-}Ad}$), 36.0 (d, $^5J_{CP}$ = 1.9 Hz, $CH_{2\text{-}Ad}$), 29.4 (d, $^4J_{CP}$ = 9.6 Hz, $C_{H\text{-}Ad}$); **$^{31}P\{^1H\}$ NMR** (121.5 MHz, acetone-d6) δ 88.4 (PAd_2), -144.3 (sept, $^1J_{FP}$ = 712 Hz, P_{PF6}); **$^{19}F\{^{13}C\}$ NMR** (282.4 MHz, acetone-d6): δ -72.7 (d, $^1J_{PF}$ = 707 Hz, F_{PF6}); **^{15}N NMR** (50.7 MHz, acetone-d6): δ 68.5 (N-NMe_2); HRMS (ESI-) calcd. for $[HOcatO(Cl)^{35}]^-$ = $C_6HCl_4O_2$: 246.8701, found 246.8698, calcd. for $[HOcatO(Cl)^{37}]^-$: 248.8672, found 248.8668; calcd. for $[PF_6]^-$: 144.9642, found 144.9640.

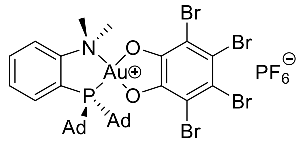

PNAu(catBr4)$^+$PF6$^-$ (5b). To a solution of **$AgPF_6$** (10^{-1}M, 100 µL, 0.01 mmol, 1 equiv.) in CD_2Cl_2 a solution of **4** (6.5 mg, 0.01 mmol, 1 equiv.) in CD_2Cl_2 (0.4 mL) was added at -20 °C. Then the temperature was raised to r.t., while a pale yellow precipitate formed gradually. After 30 minutes at r.t. the suspension was syringe filtered. To this solution, a CD_2Cl_2 solution of ***o*-bromanil** (10^{-1}M, 100 µL, 1 equiv.) was added at -20 °C. After 30 minutes at r.t. the solution was analyzed by multinuclear NMR. The title product was not isolated. It was formed in 100% NMR yield. Single crystals were obtained by slow evaporation of the solvent from the NMR sample. **^1H NMR** (300.1 MHz, CD_2Cl_2): δ 8.18-7.99 (m, 2H, H_{Ar}), 7.94-7.79 (m, 2H, H_{Ar}), 3.93 (s, 6H, NMe_2), 2.48-2.29 (m, 12H, H_{Ad}), 2.21-2.11 (m, 6H, H_{Ad}), 1.85-1.74 (m, 12H, H_{Ad}); **$^{31}P\{^1H\}$ NMR** (121.5 MHz, CD_2Cl_2) δ 87.6 (PAd_2), -144.3 (sept, $^1J_{FP}$ = 712 Hz, P_{PF6}); **$^{19}F\{^{13}C\}$ NMR** (282.4 MHz, CD_2Cl_2): δ -72.8 (d, $^1J_{PF}$ = 711 Hz, F_{PF6}).

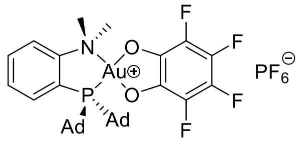

PNAu(catF4)$^+$PF6$^-$ (5c). To a solution of **$AgPF_6$** (10^{-1}M, 100 µL, 0.01 mmol, 1 equiv.) in CD_2Cl_2 a solution of **MeDalPhosAuCl** (6.5 mg, 0.01 mmol, 1 equiv.) in CD_2Cl_2 (0.4 mL) was added at -20 °C. Then the temperature was raised to r.t., while a pale yellow precipitate formed gradually. After 30 minutes at r.t. the suspension was syringe filtered. To this solution, a CD_2Cl_2 solution of ***o*-fluoranil** (10^{-1}M, 100 µL, 1 equiv.) was added at -20 °C. After 30 minutes at r.t., the solution was analyzed by multinuclear NMR: ~95% yield, ~95% purity. Single crystals were obtained by slow evaporation of the solvent from the NMR sample. **^1H NMR** (300.1 MHz, CD_2Cl_2): δ 8.13-7.99 (m, 2H, H_{Ar}), 7.93-7.78 (m, 2H, H_{Ar}), 3.92 (s, 6H, NMe_2), 2.46-2.23 (m, 12H, H_{Ad}), 2.21-2.10 (m, 6H, H_{Ad}), 1.84-1.75 (m, 12H, H_{Ad}); **$^{31}P\{^1H\}$ NMR** (121.5 MHz, CD_2Cl_2) δ 87.4 (PAd_2), -144.3 (sept, $^2J_{FP}$ = 712 Hz, P_{PF6}); **$^{19}F\{^1H\}$ NMR** (282.4 MHz, CD_2Cl_2): δ -72.1 (s, 3F, F_{PF6}), -74.6 (s, 3F, F_{PF6}), -168.7 (dt, 1F, $^3J_{FF}$ = 21.5 Hz, $^{4,5}J_{FF}$ = 6.3 Hz), -169.0 (d, 1F, $^3J_{FF}$ = 21.9 Hz, $^{4,5}J_{FF}$ = 6.5 Hz), -174.3 (td, 1F, $^3J_{FF}$ = 21.9 Hz, $^4J_{FF}$ = 6.2 Hz), -175.6 (td, 1F, $^3J_{FF}$ = 21.8 Hz, $^4J_{FF}$ = 6.3 Hz).

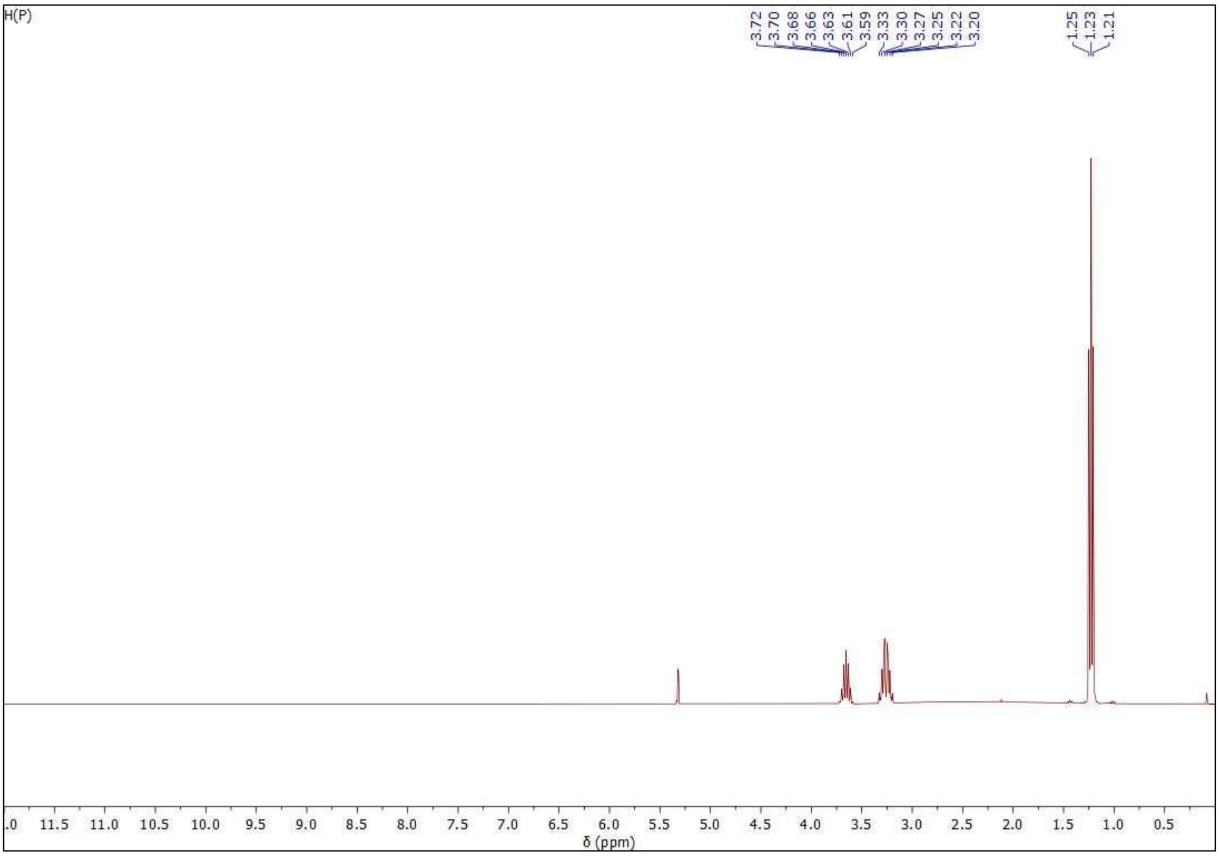

Figure S1. $^{1}H\{^{31}P\}$ NMR spectrum of **1** in CD_2Cl_2.

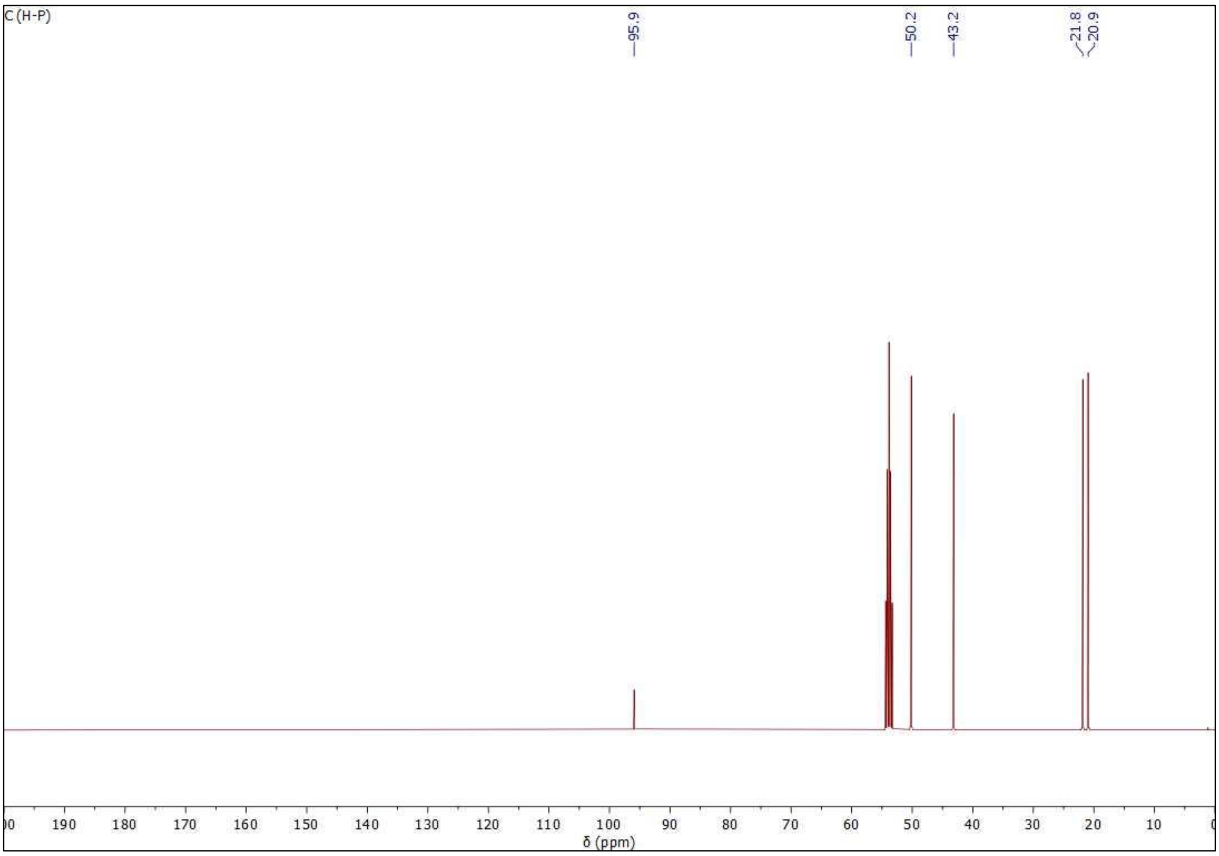

Figure S2. $^{13}C\{^{1}H,^{31}P\}$ NMR spectrum of **1** in CD_2Cl_2.

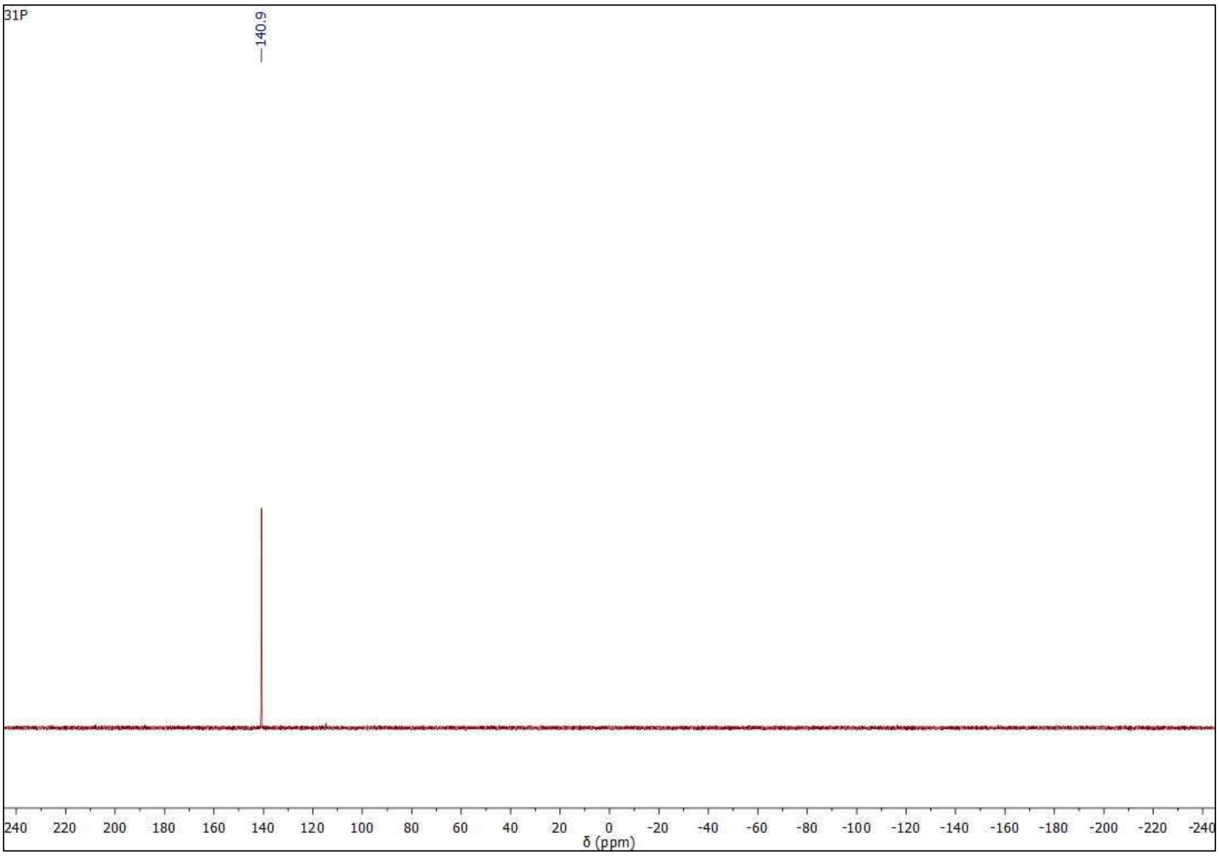

Figure S3. $^{31}P\{^1H\}$ NMR spectrum of **1** in CD_2Cl_2.

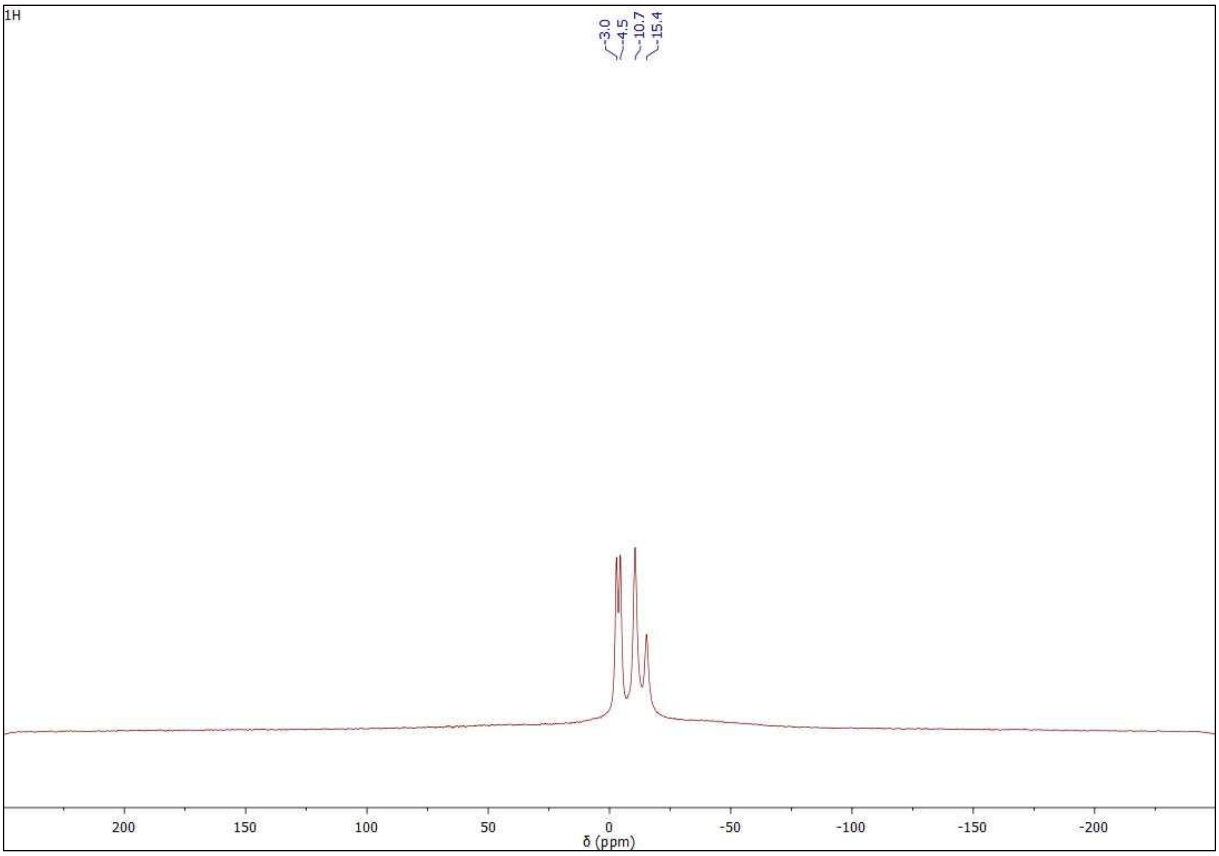

Figure S4. ^{11}B NMR spectrum of **1** in CD_2Cl_2.

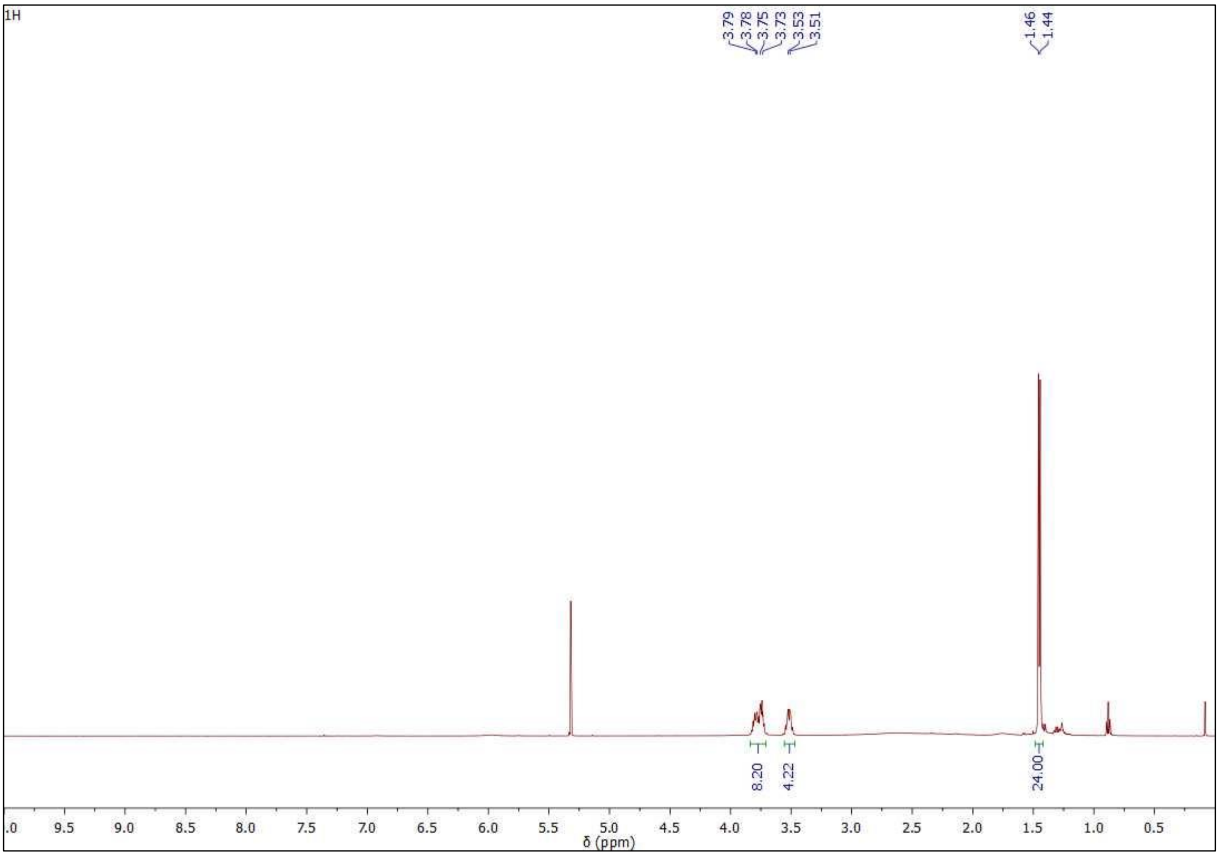

Figure S5. ^{1}H NMR spectrum of **2a** in CD_2Cl_2.

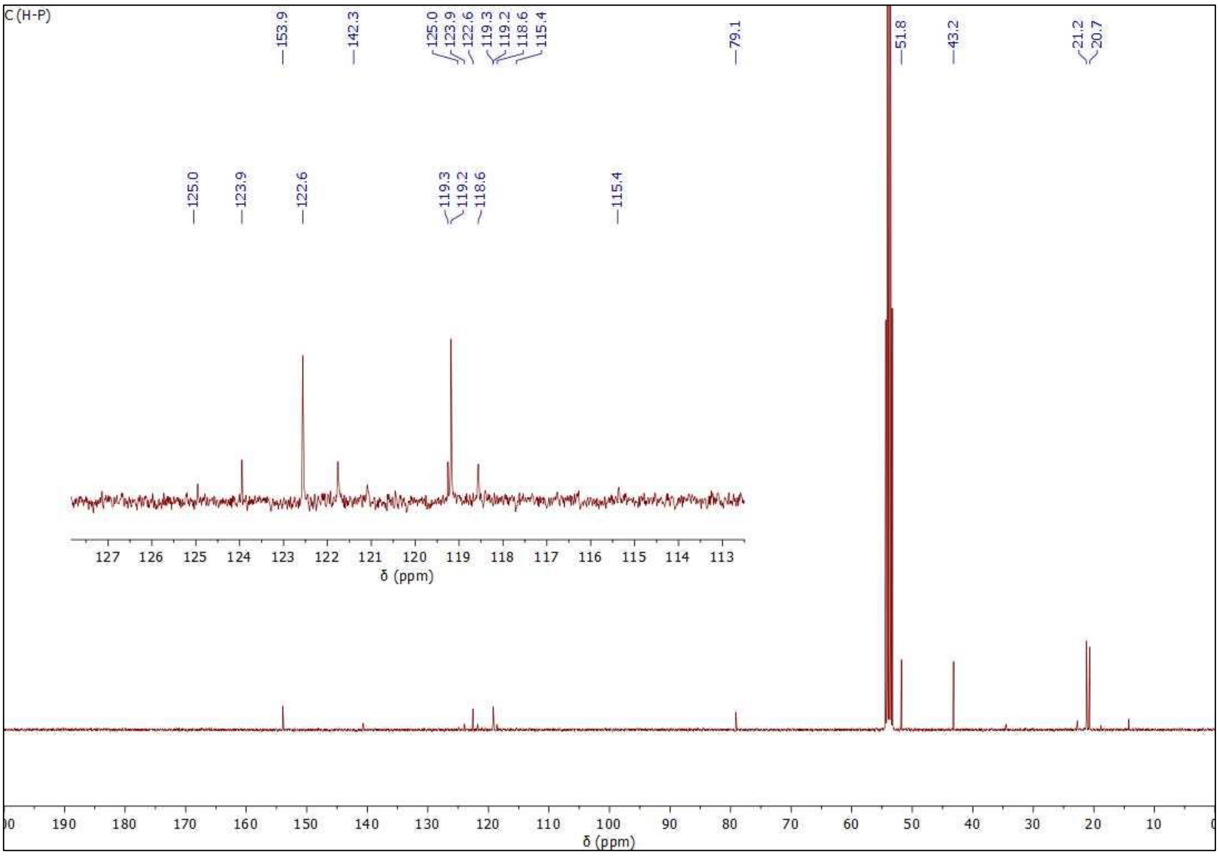

Figure S6. $^{13}C\{^{1}H,^{31}P\}$ NMR spectrum of **2a** in CD_2Cl_2.

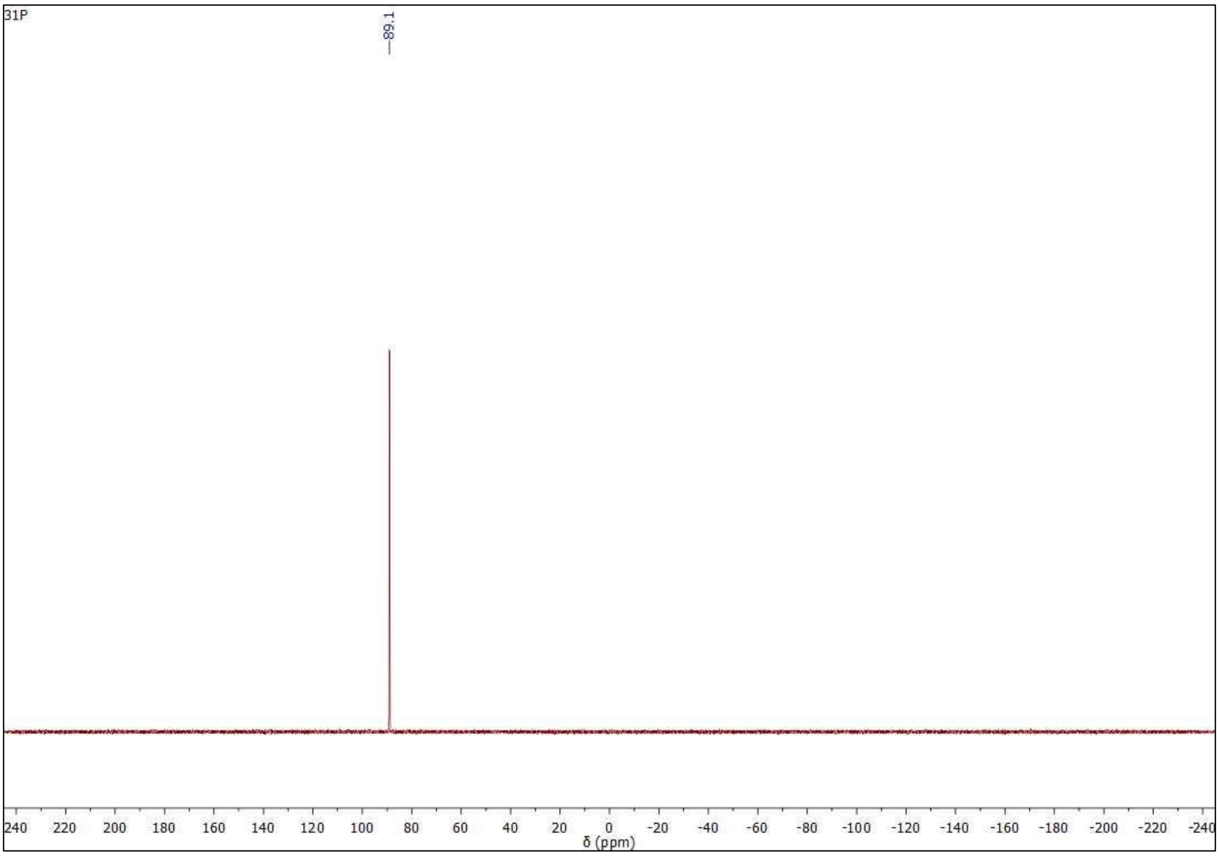

Figure S7. $^{31}P\{^1H\}$ NMR spectrum of **2a** in CD_2Cl_2.

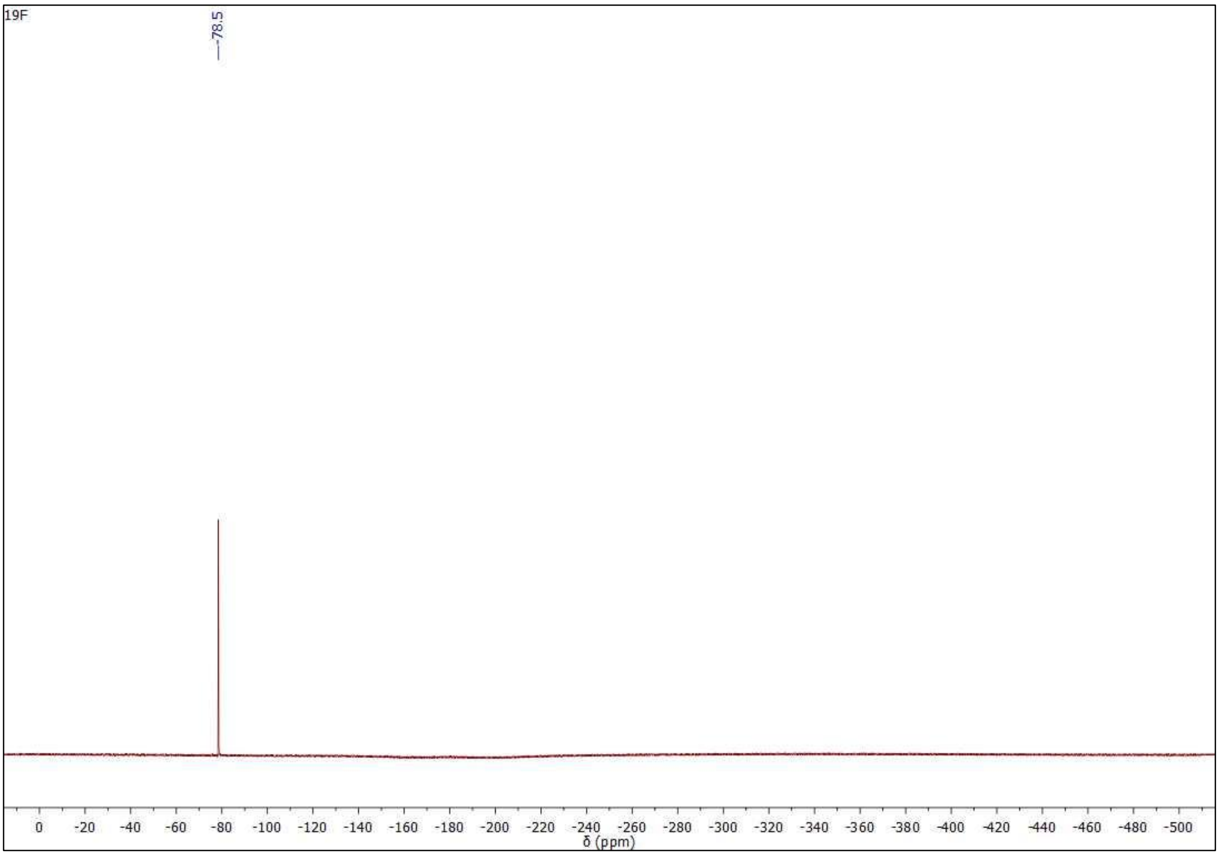

Figure S8. $^{19}F\{^1H\}$ NMR spectrum of **2a** in CD_2Cl_2.

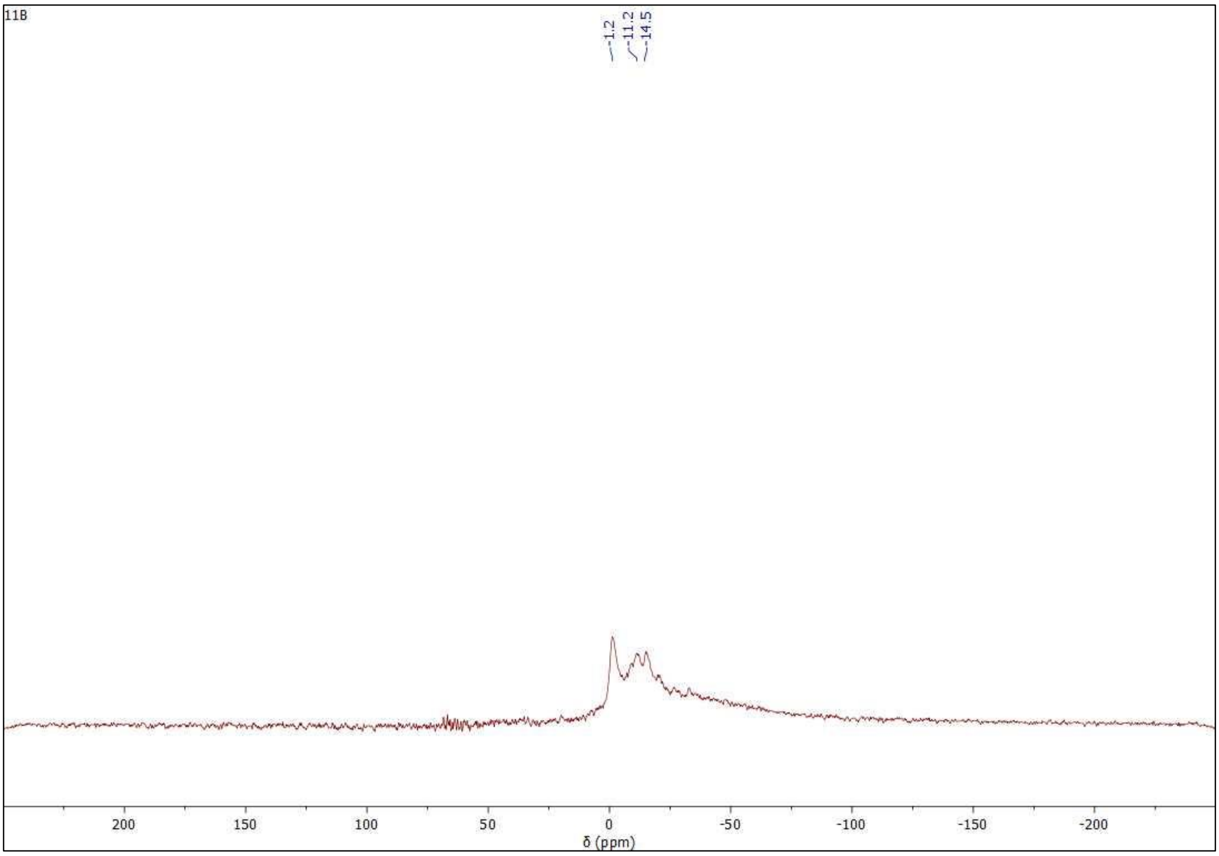

Figure S9. ^{11}B NMR spectrum of **2a** in CD_2Cl_2.

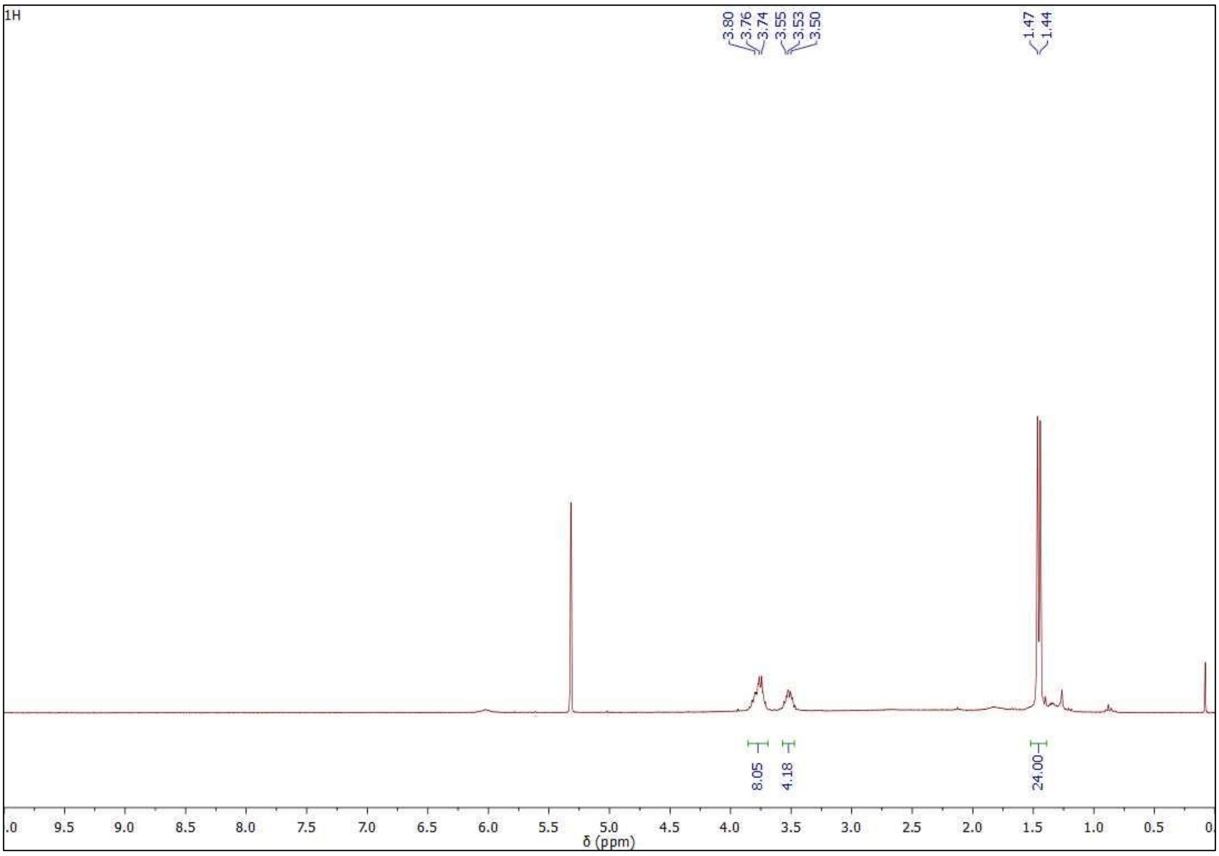

Figure S10. ^{1}H NMR spectrum of **2b** in CD_2Cl_2.

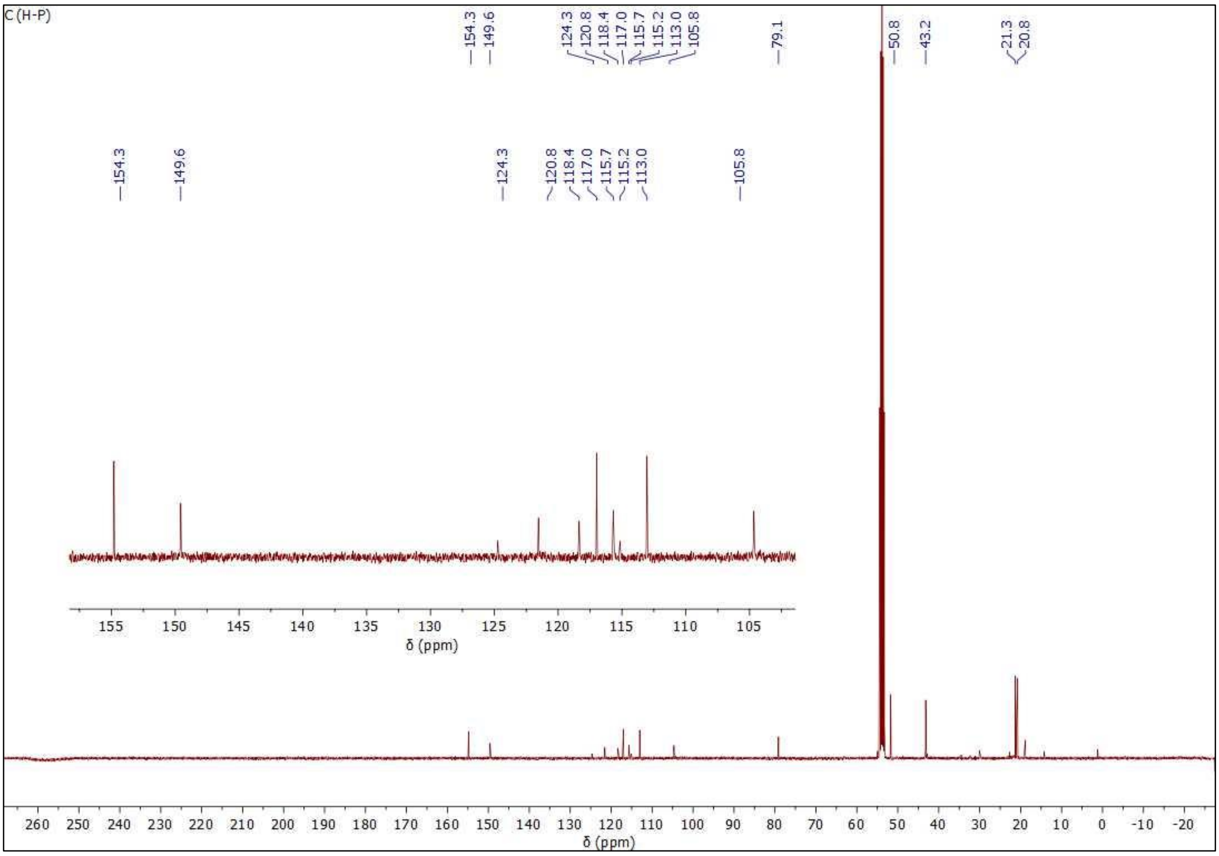

Figure S11. $^{13}C\{^{1}H\}$ NMR spectrum of **2b** in CD_2Cl_2.

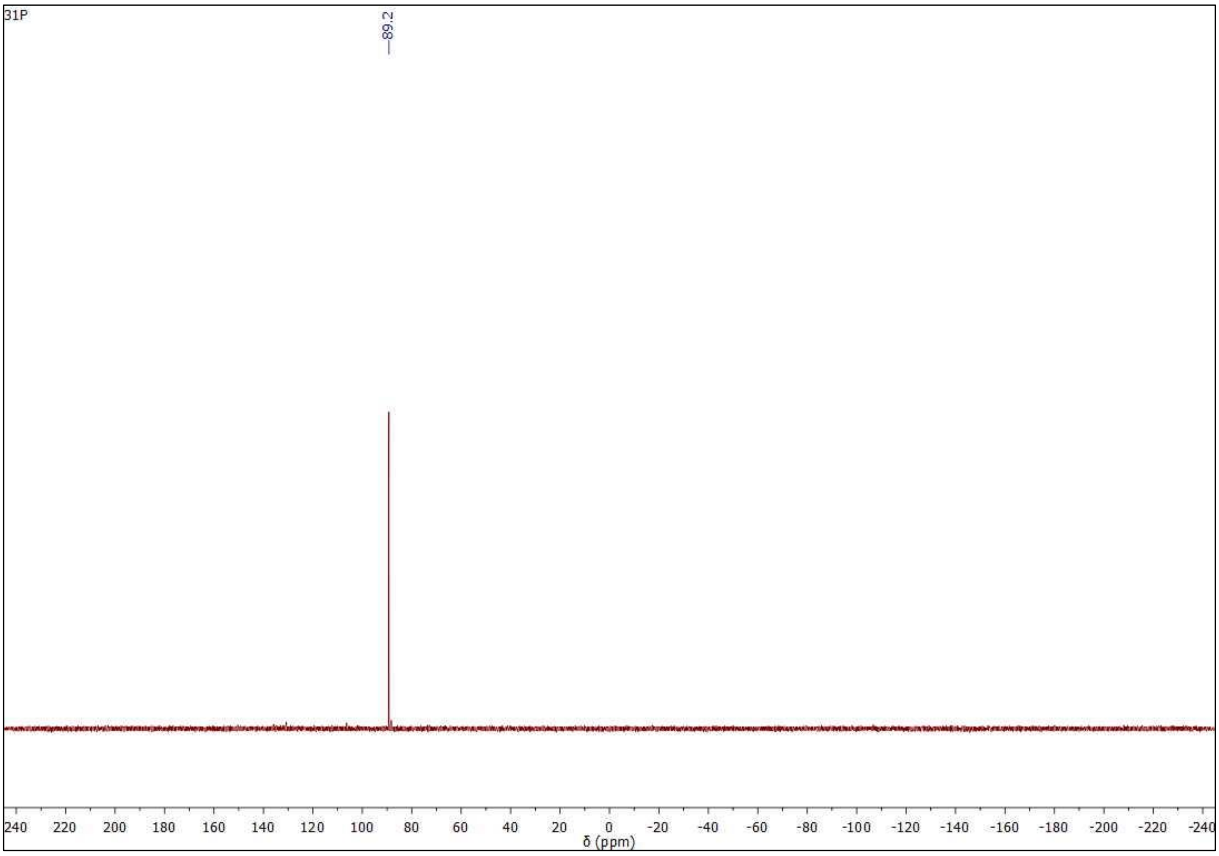

Figure S12. $^{31}P\{^{1}H\}$ NMR spectrum of **2b** in CD_2Cl_2.

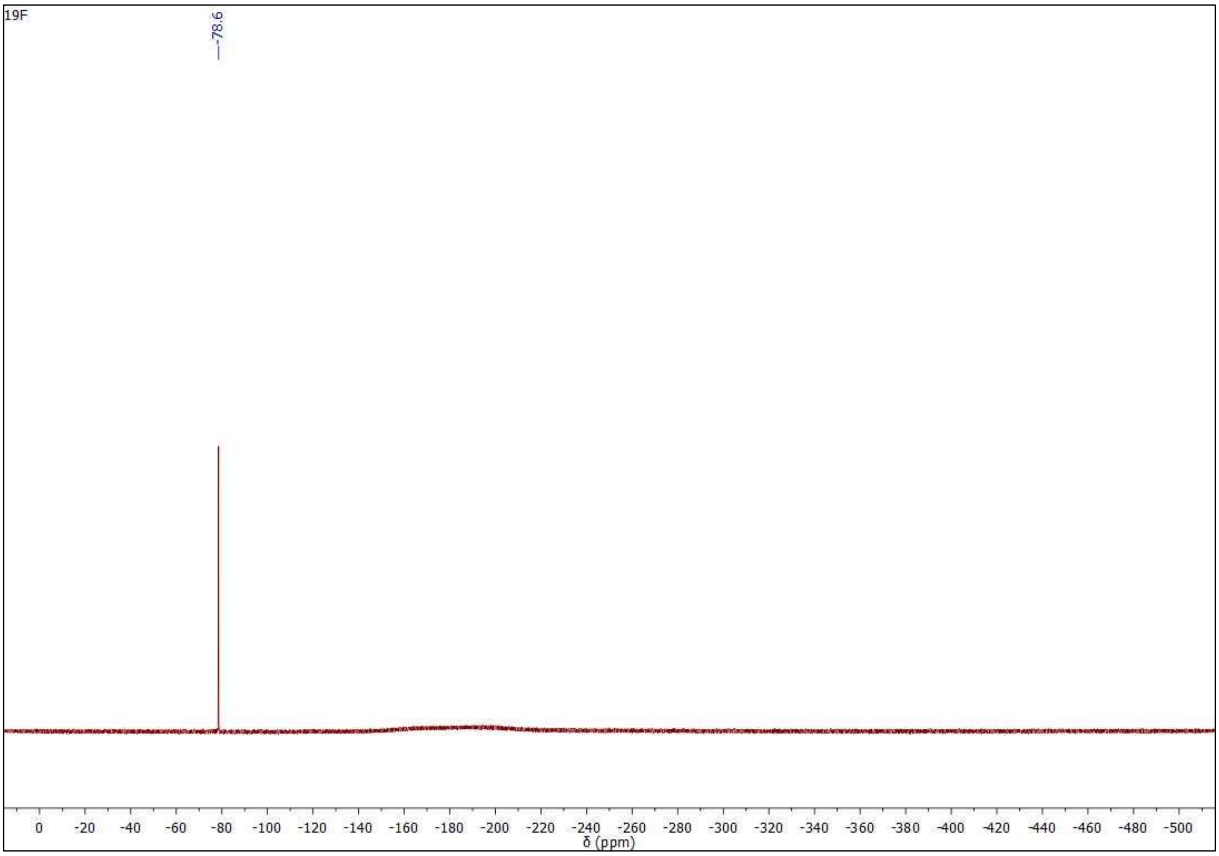

Figure S13. $^{19}F\{^{13}C\}$ NMR spectrum of **2b** in CD_2Cl_2.

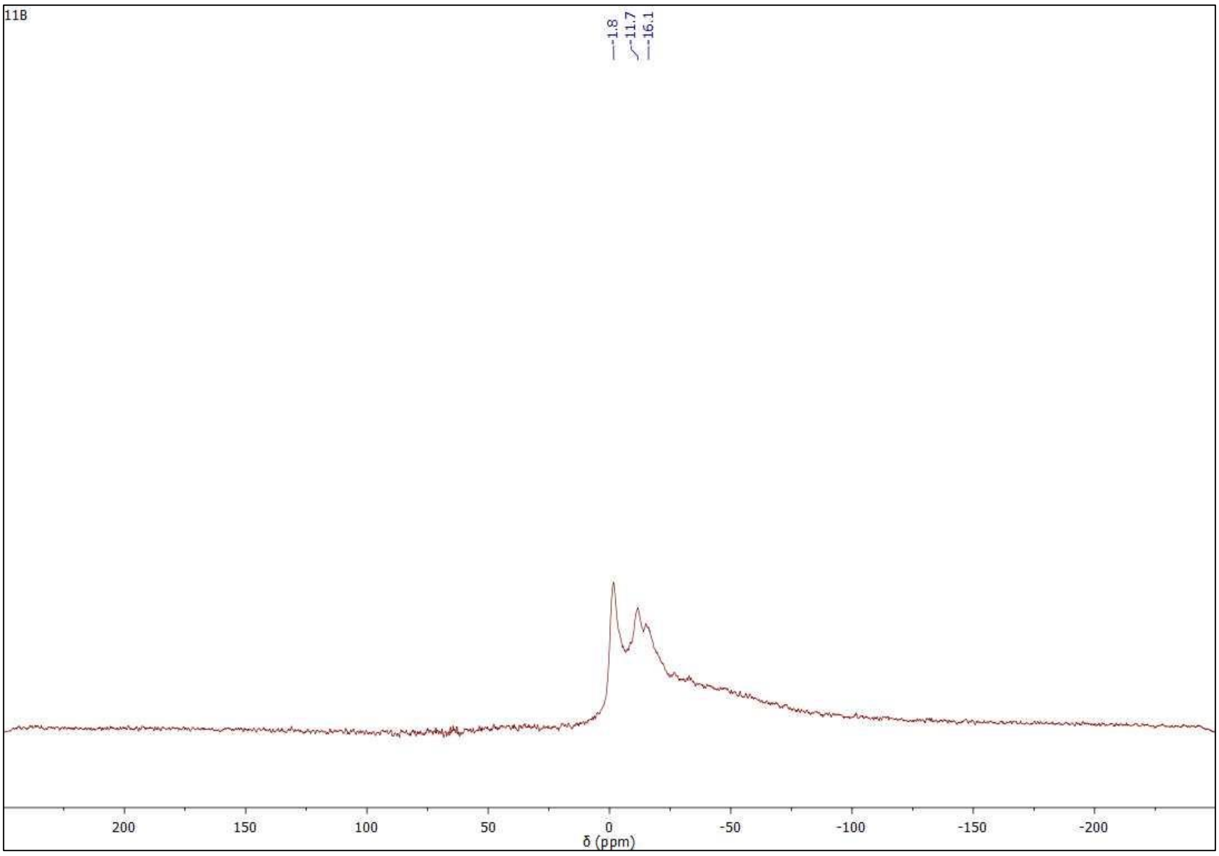

Figure S14. ^{11}B NMR spectrum of **2b** in CD_2Cl_2.

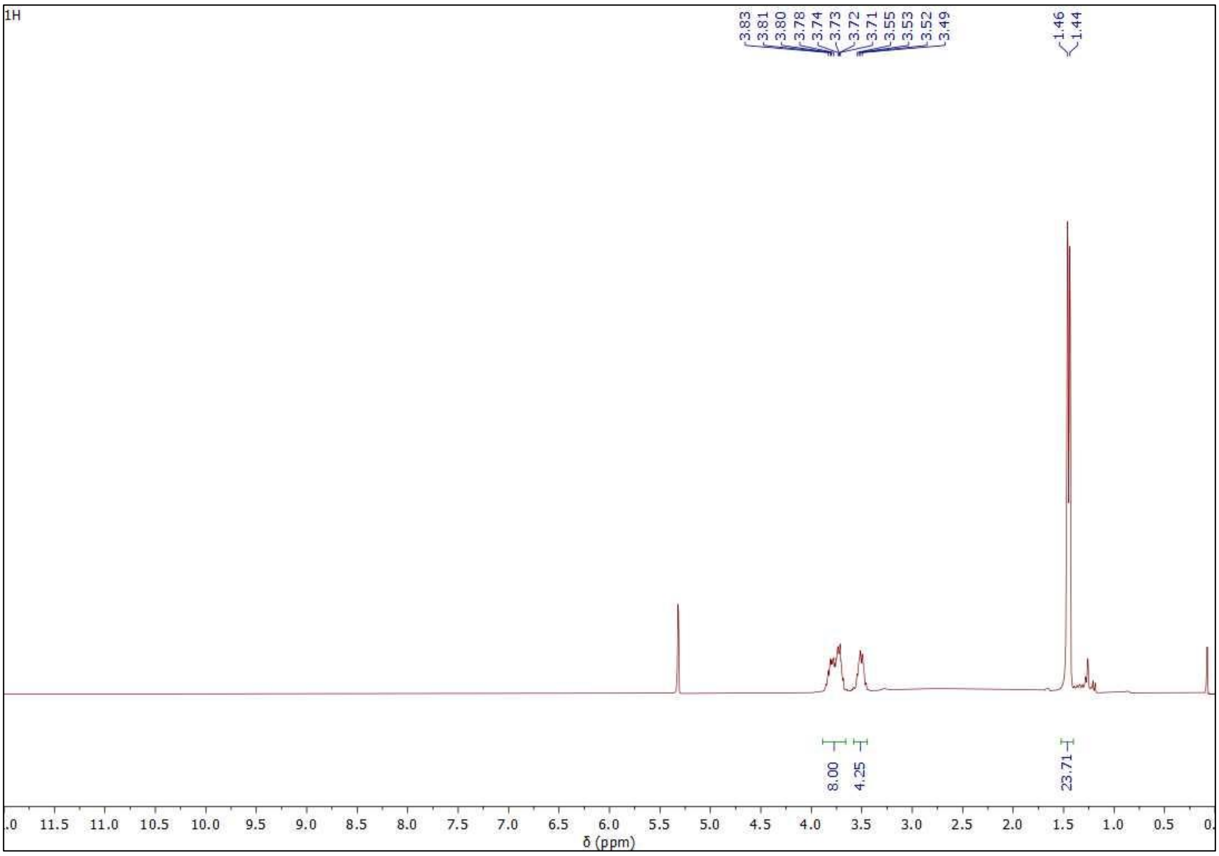

Figure S15. ^{1}H NMR spectrum of **2c** in CD_2Cl_2.

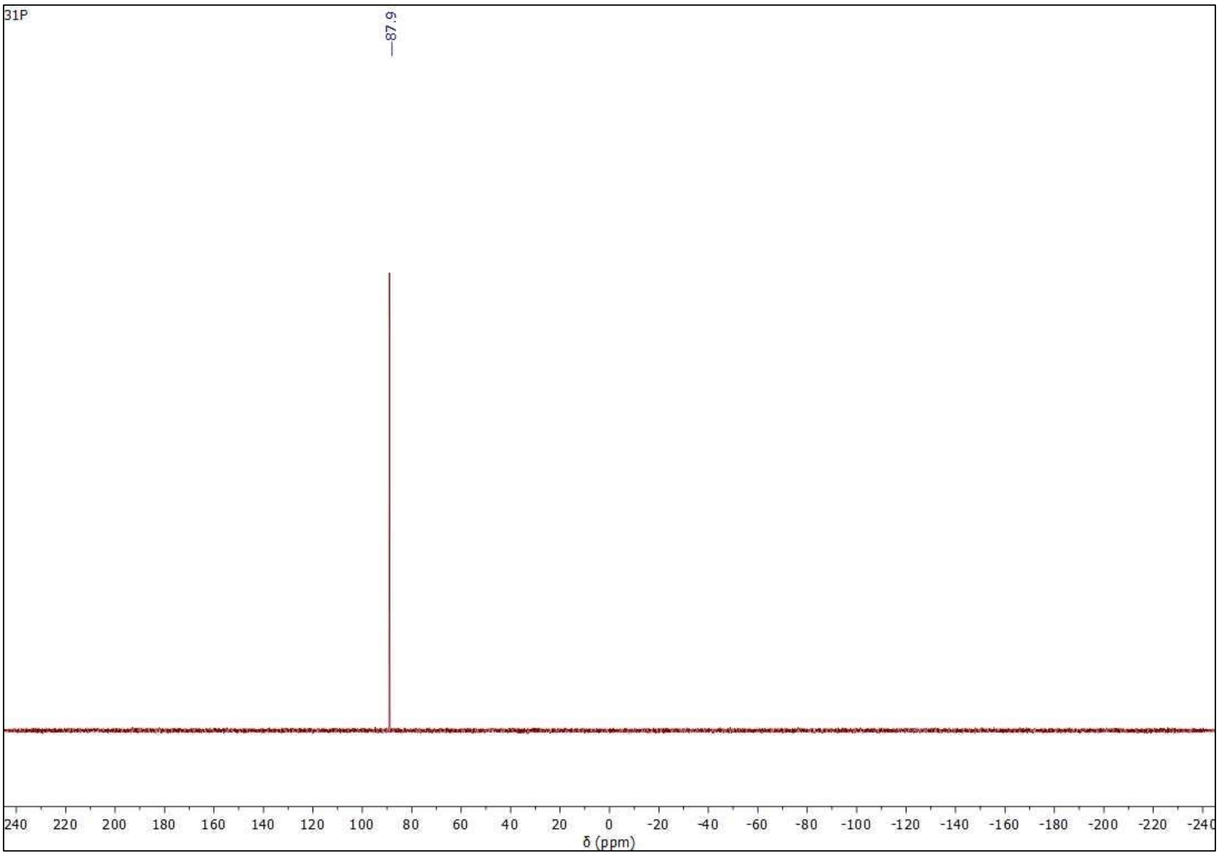

Figure S16. $^{31}P\{^{1}H\}$ NMR spectrum of **2c** in CD_2Cl_2.

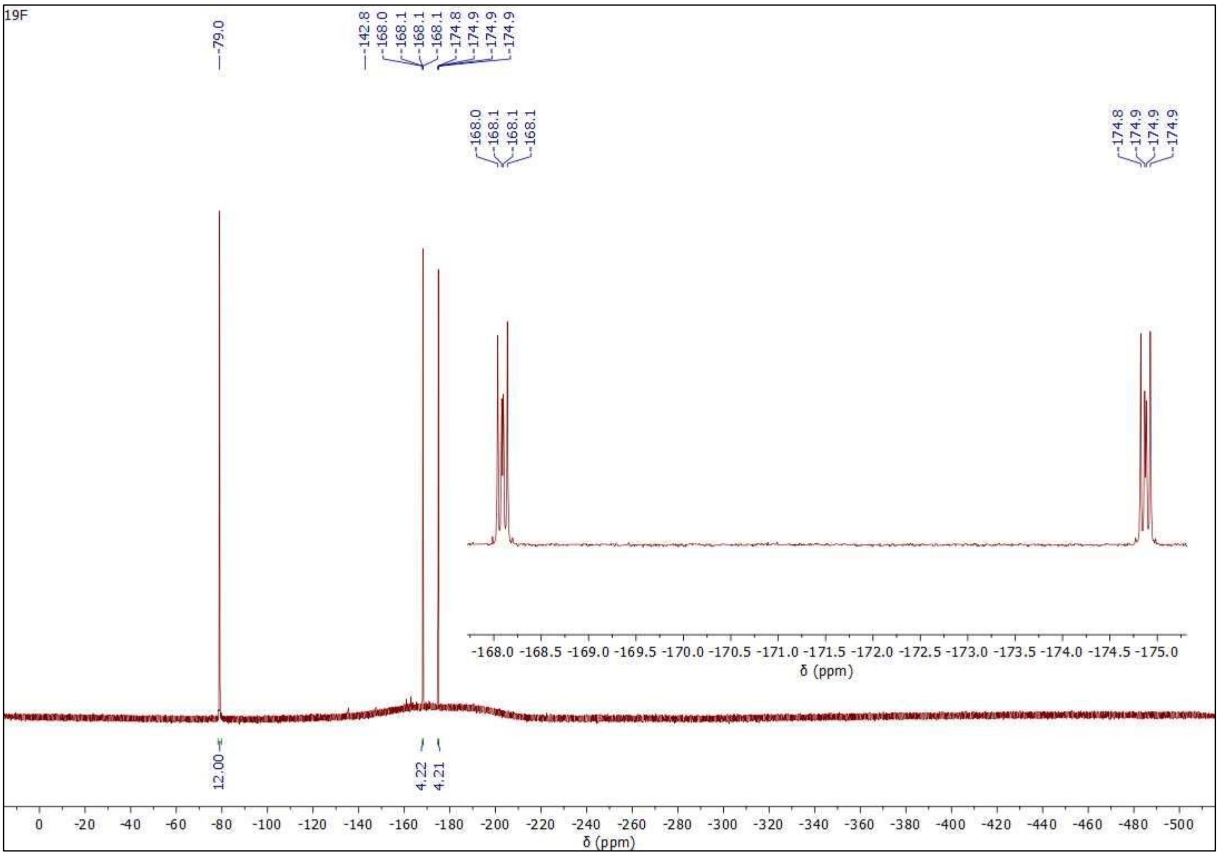

Figure S17. $^{19}F\{^1H\}$ NMR spectrum of **2c** in CD_2Cl_2.

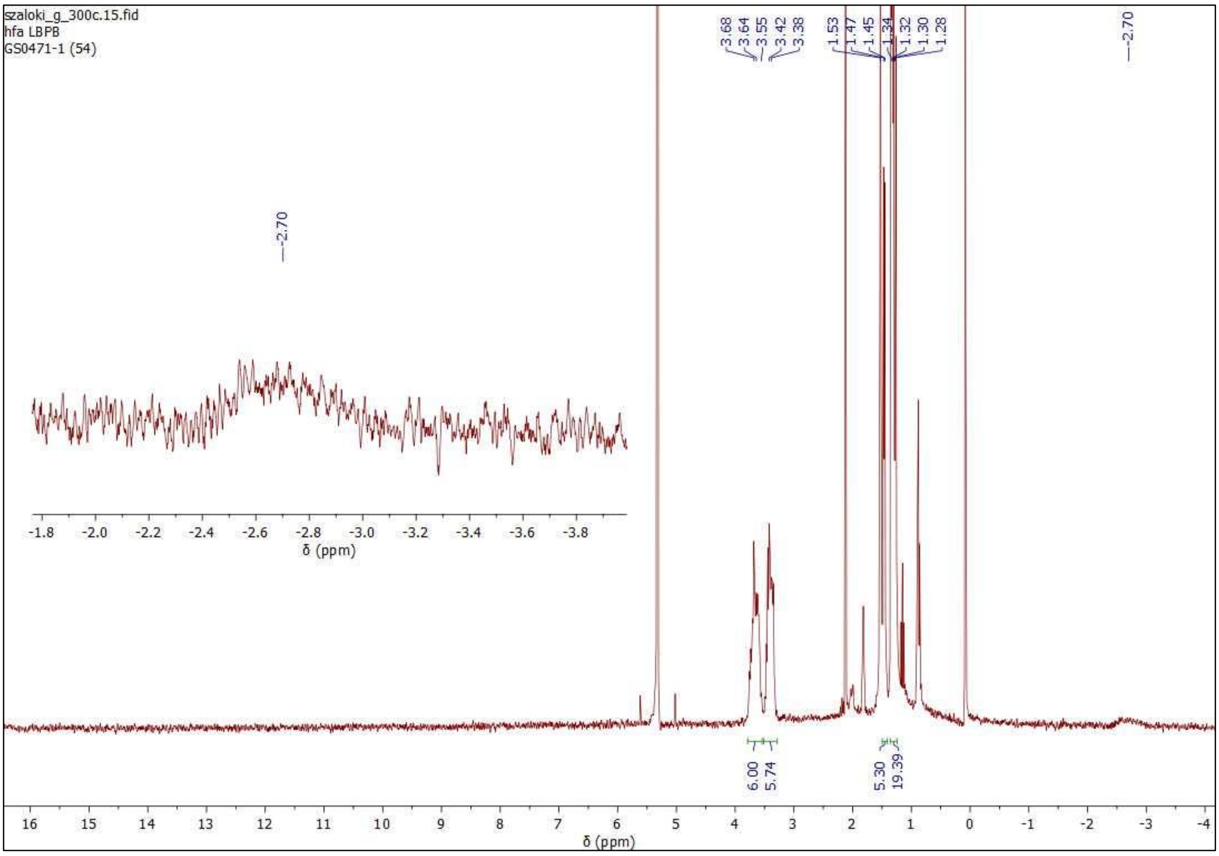

Figure S18. ^1H NMR spectrum of **3b** in CD_2Cl_2.

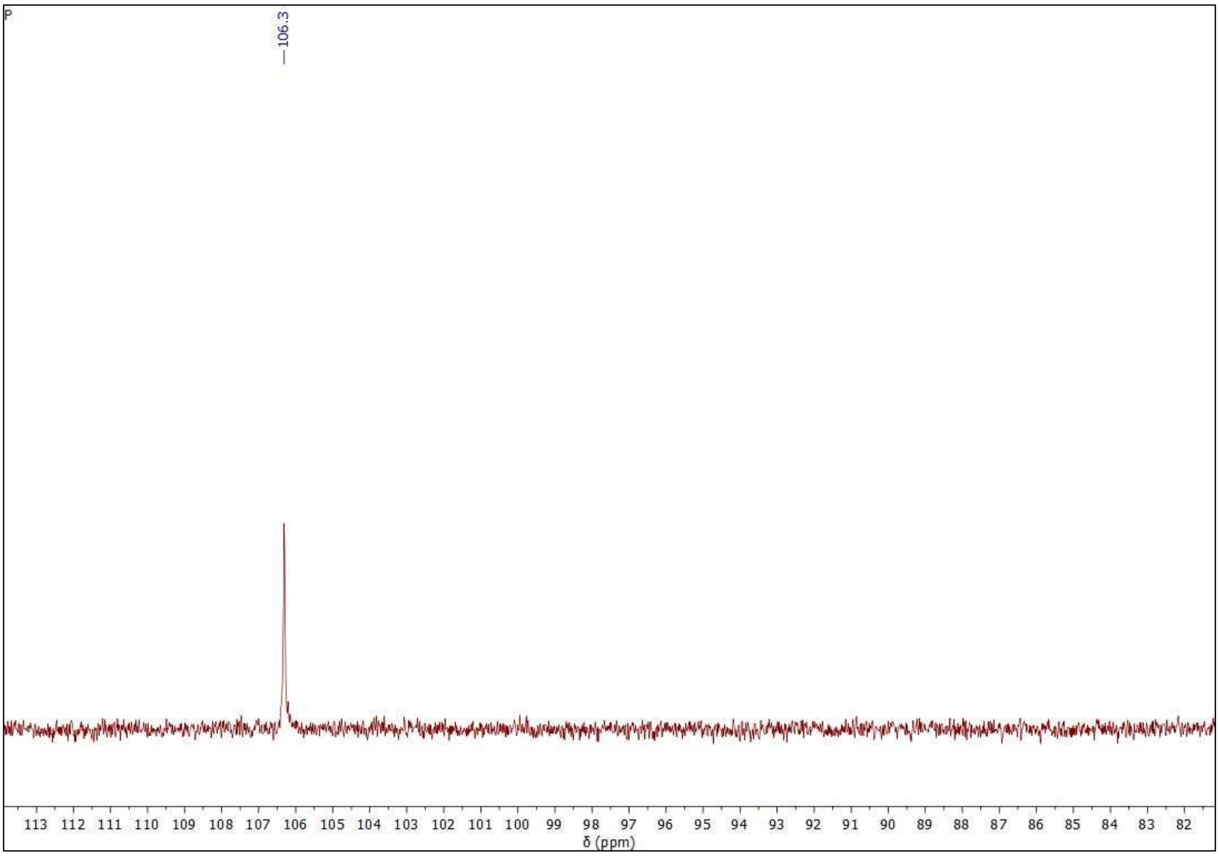

Figure S19. $^{31}P\{^{1}H\}$ NMR spectrum of **3b** in CD_2Cl_2.

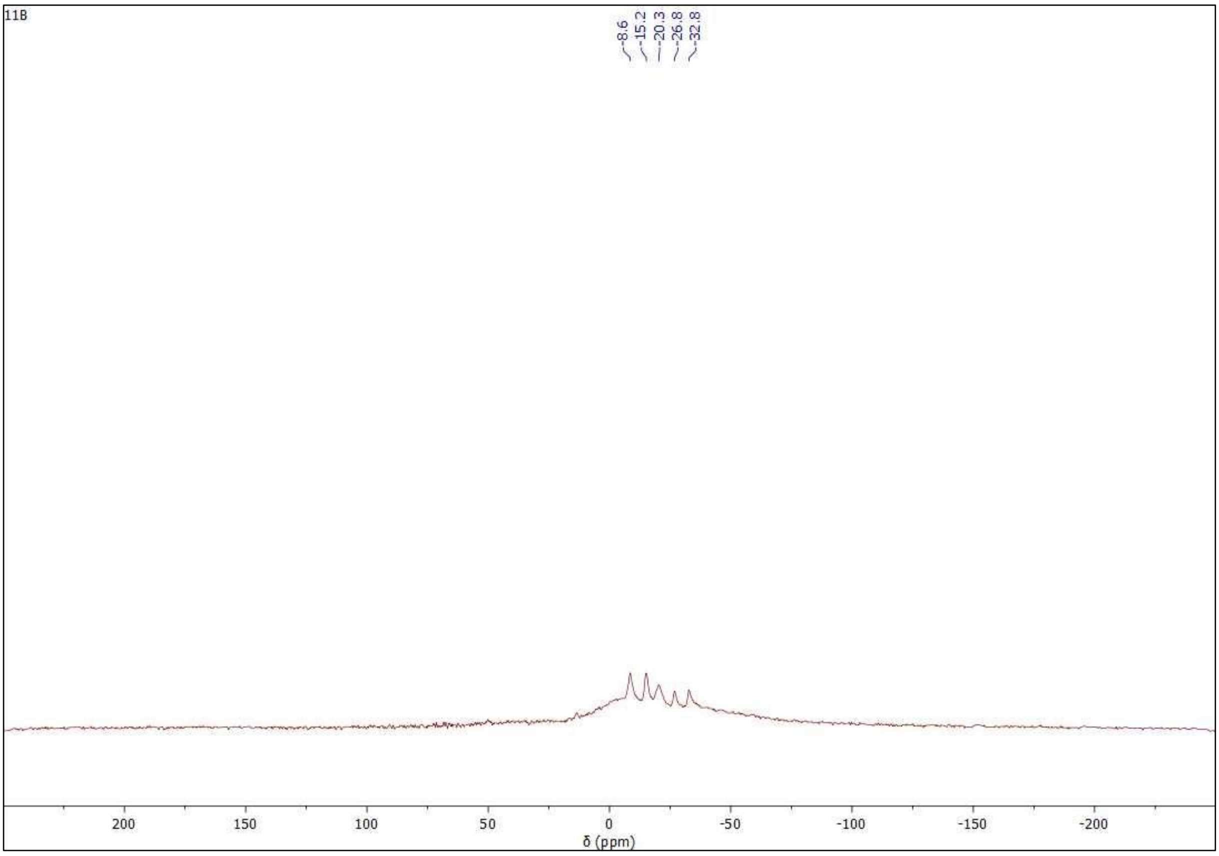

Figure S20. ^{11}B NMR spectrum of **3b** in CD_2Cl_2.

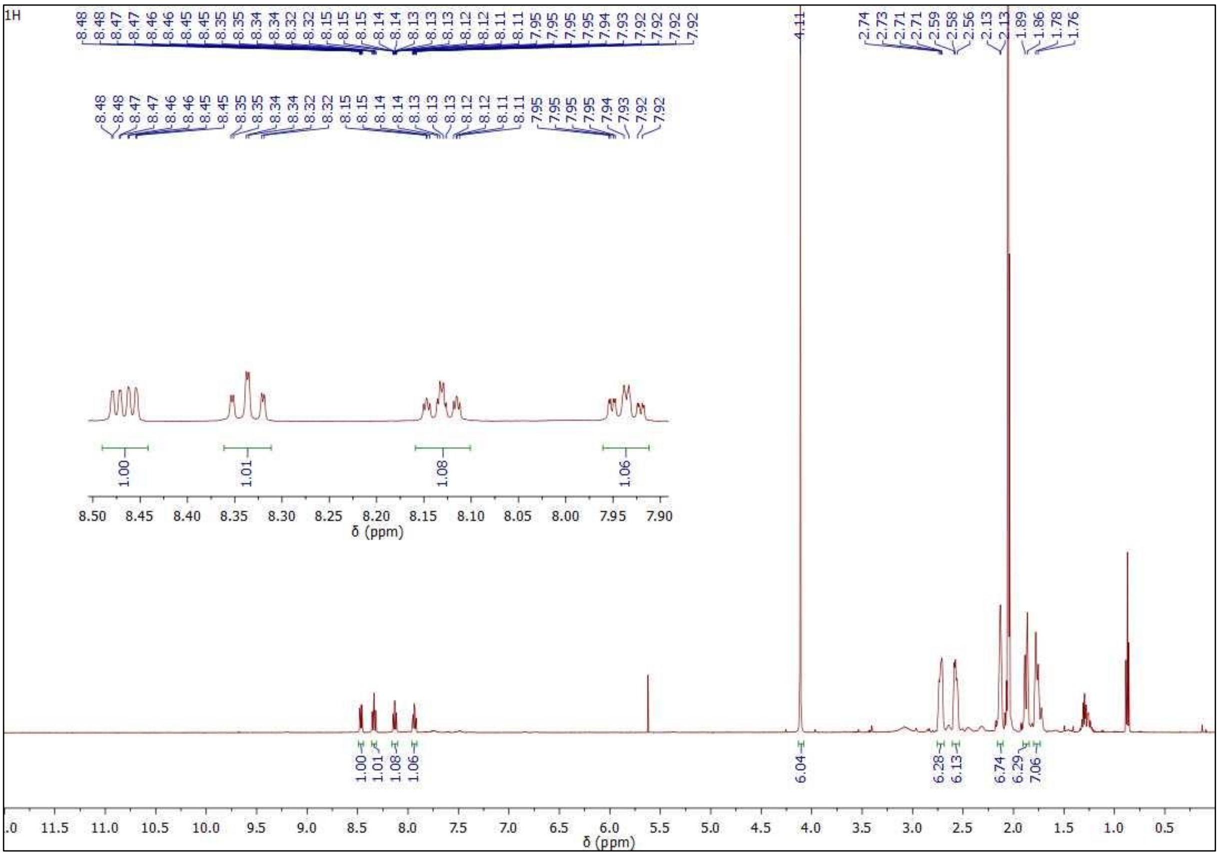

Figure S21. ^{1}H NMR spectrum of **5a** in acetone-d_6.

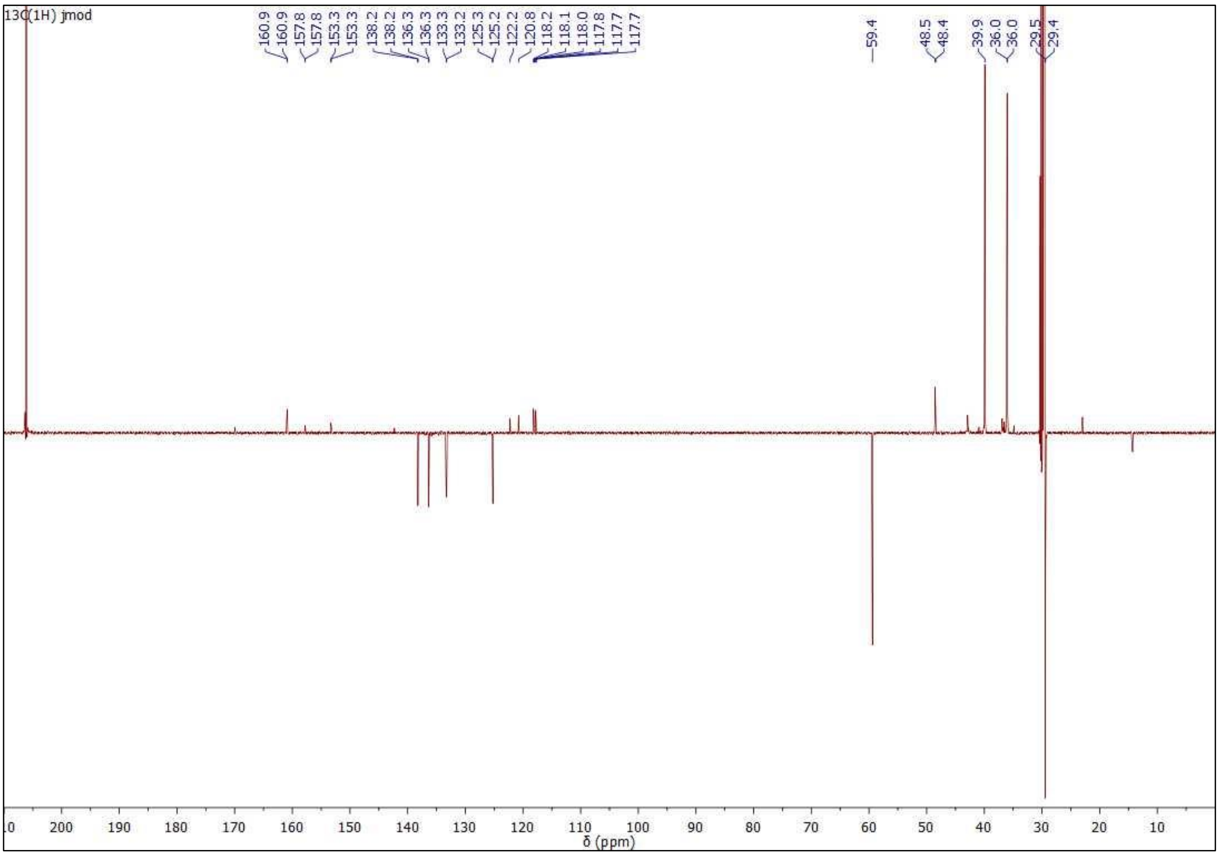

Figure S22. $^{13}C\{^{1}H\}$ NMR spectrum of **5a** in acetone-d_6.

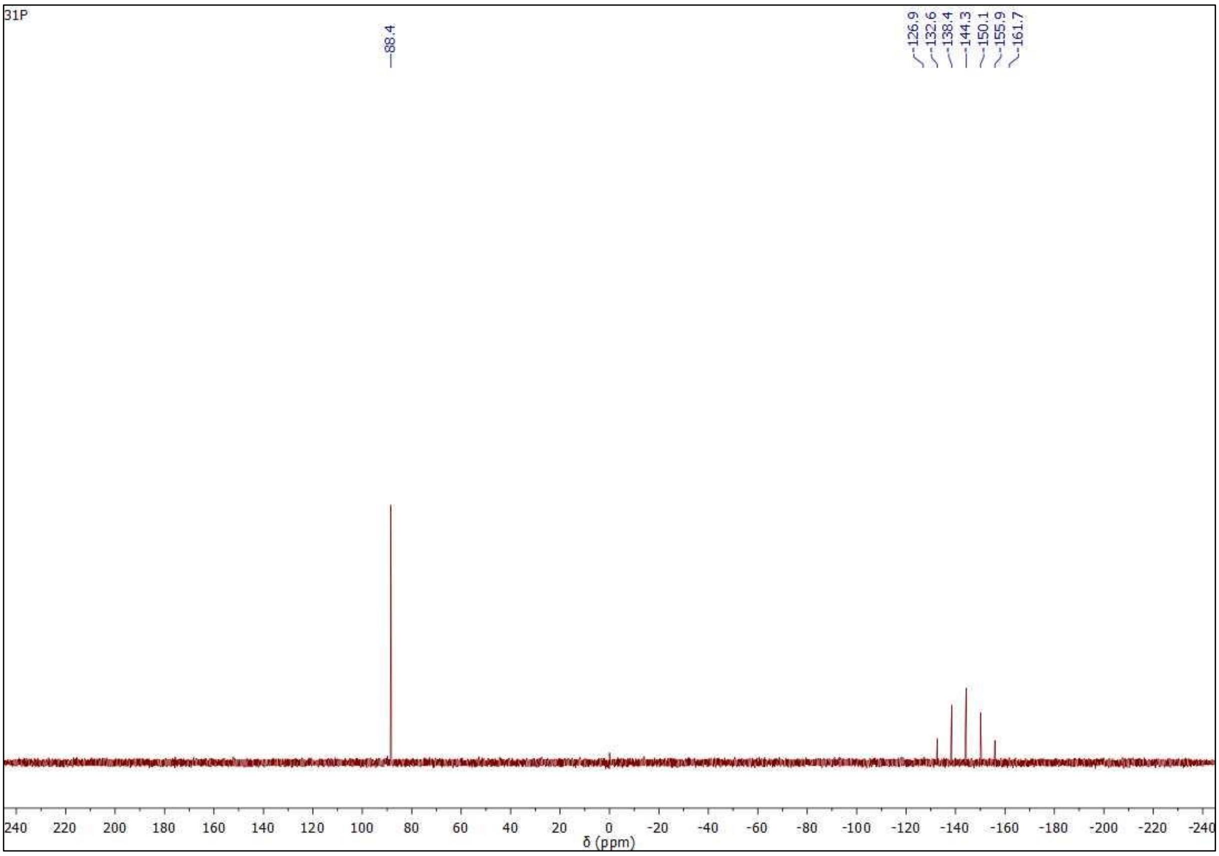

Figure S23. $^{31}P\{^1H\}$ NMR spectrum of **5a** in acetone-d_6.

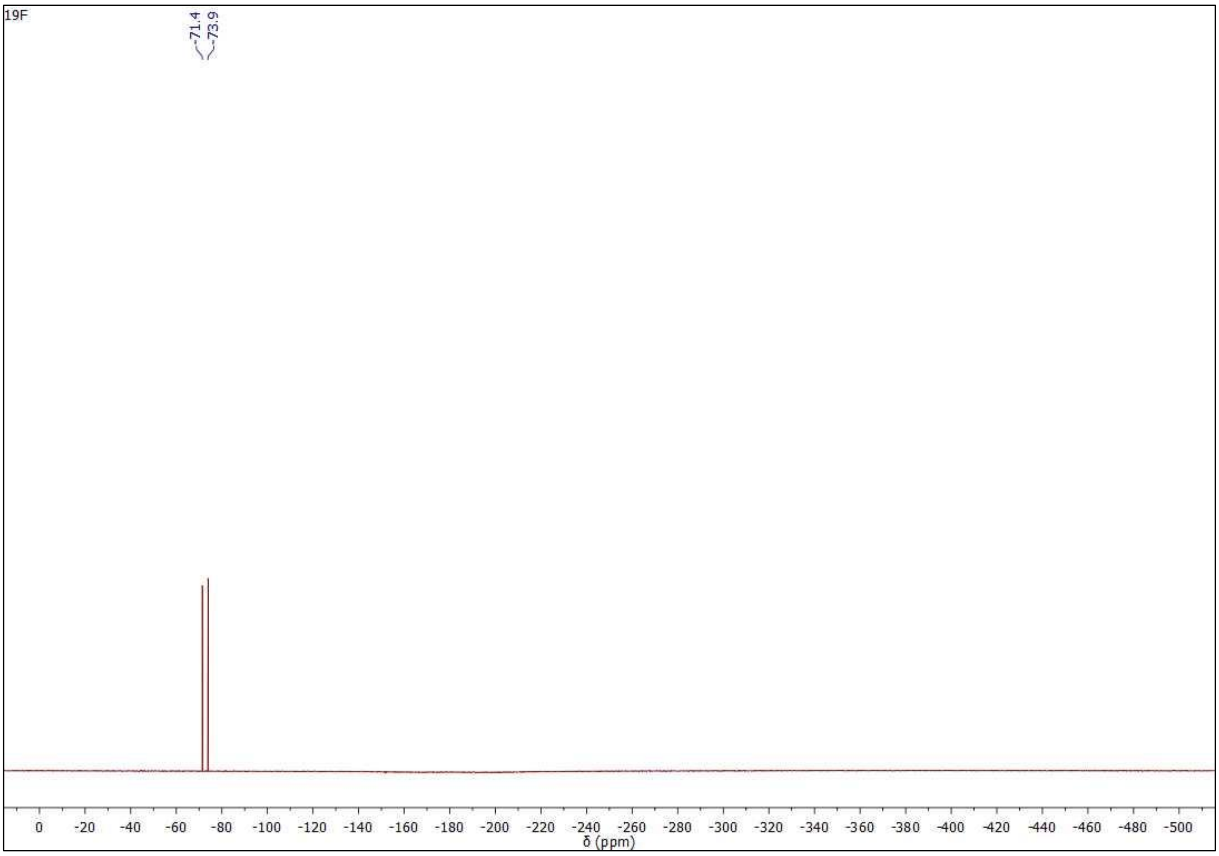

Figure S24. $^{19}F\{^1H\}$ NMR spectrum of **5a** in acetone-d_6.

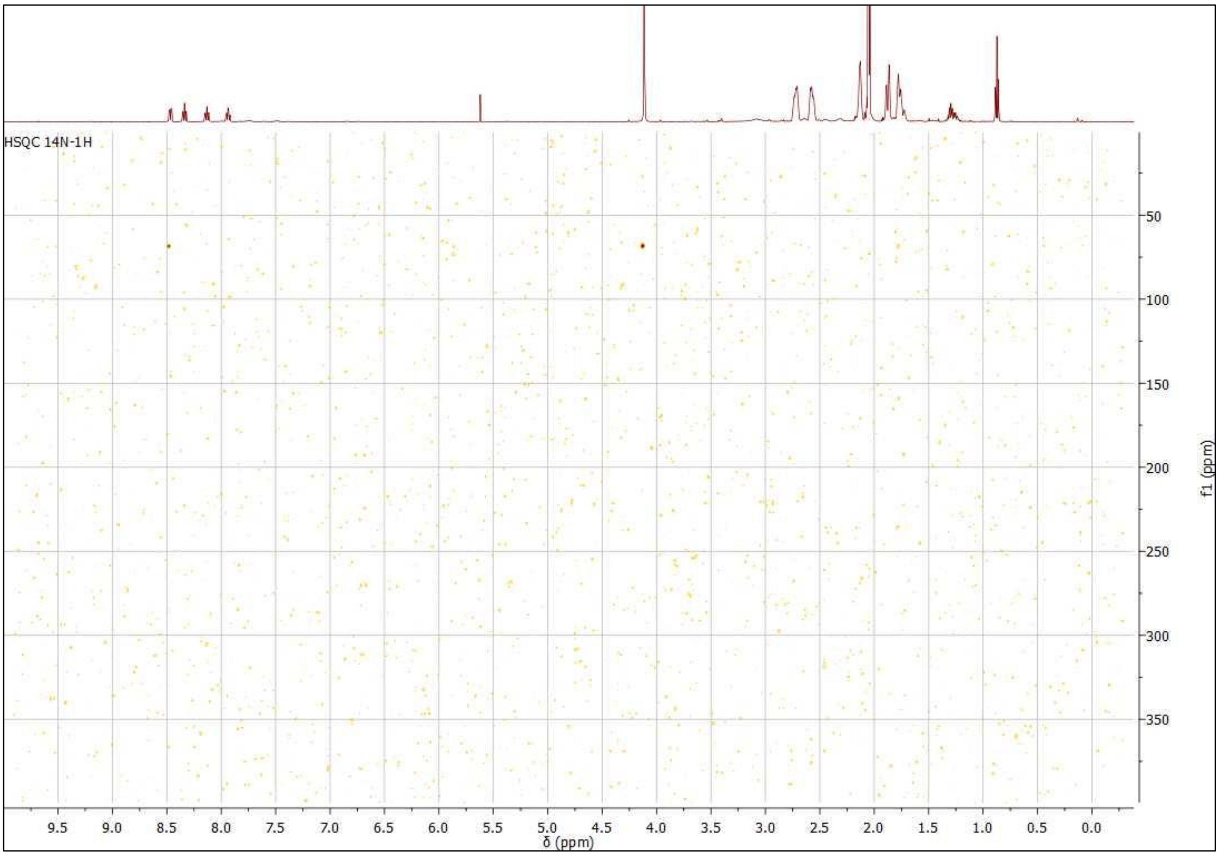

Figure S25. HSQC ^{15}N-^{1}H NMR spectrum of **5a** in acetone-d_6.

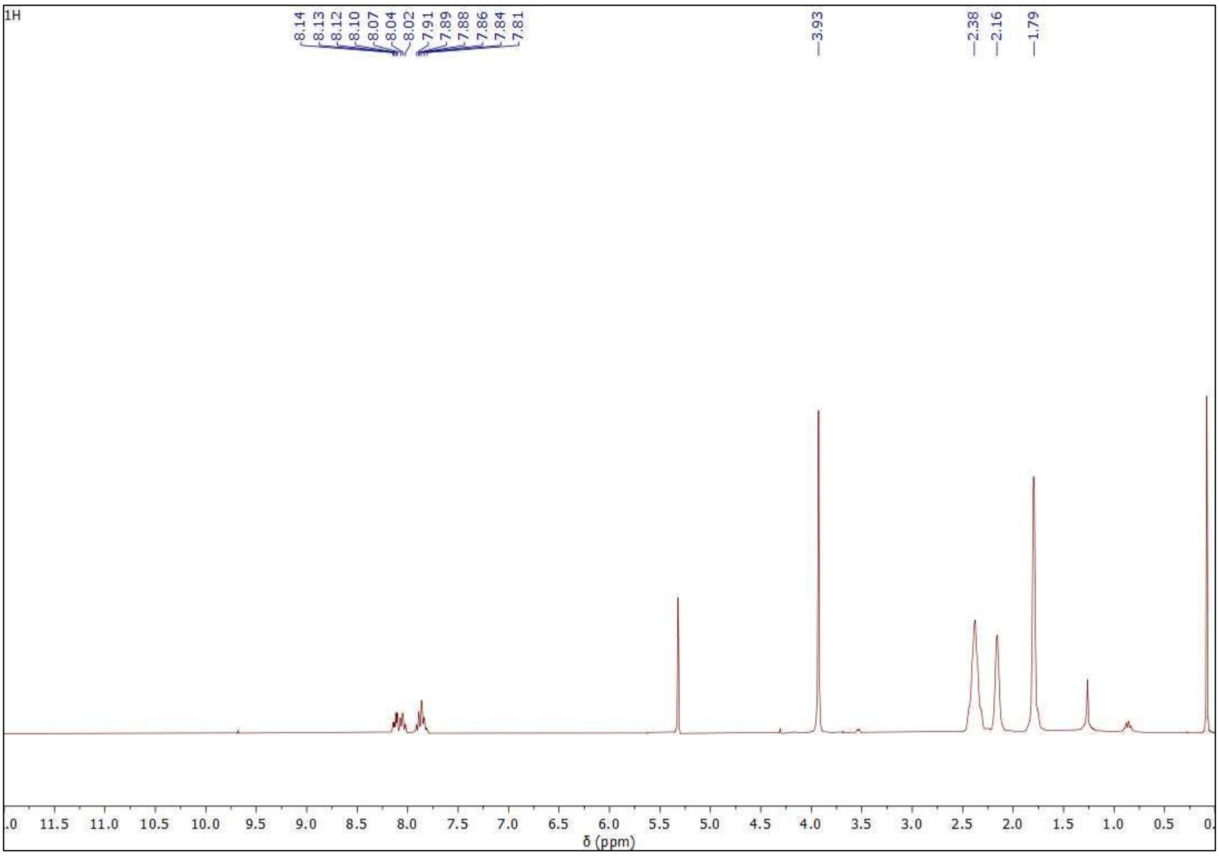

Figure S26. ^{1}H NMR spectrum of **5b** in CD_2Cl_2.

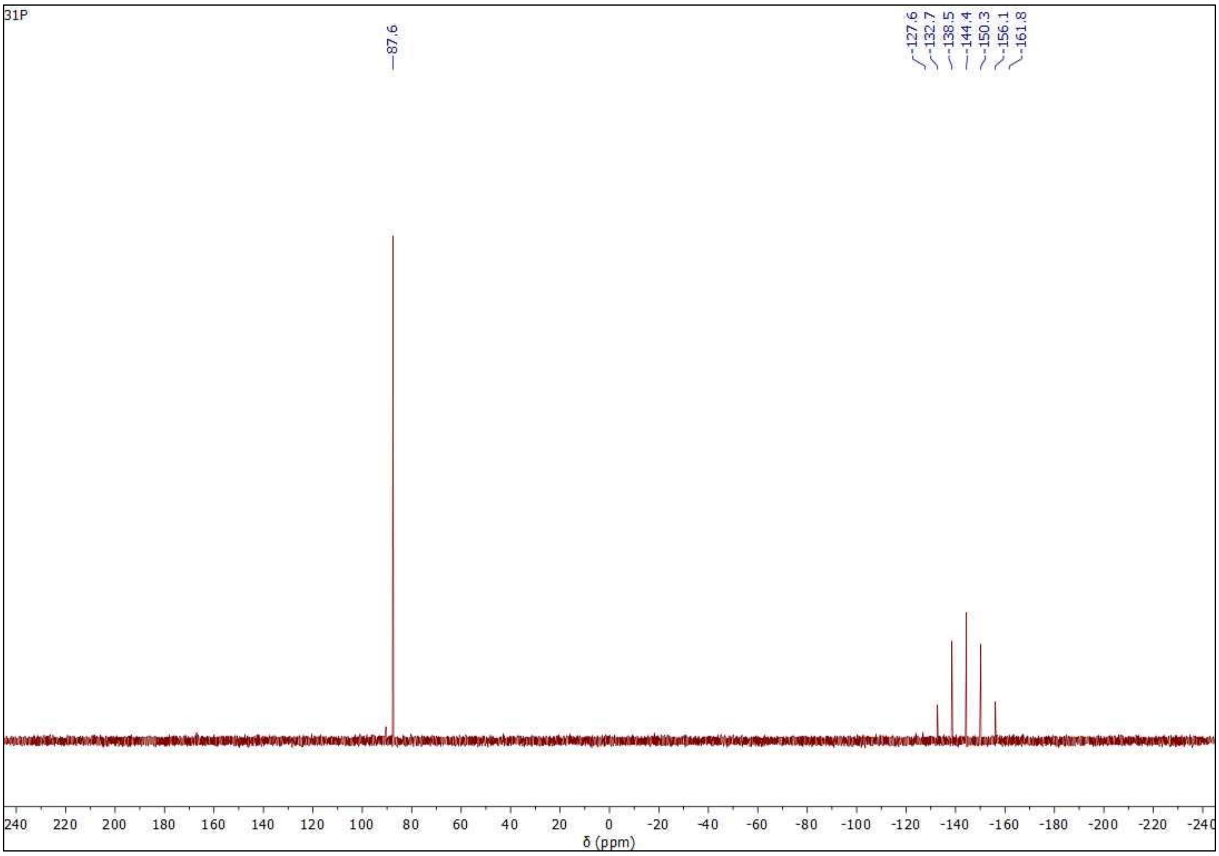

Figure S27. $^{31}P\{^{1}H\}$ NMR spectrum of **5b** in CD_2Cl_2.

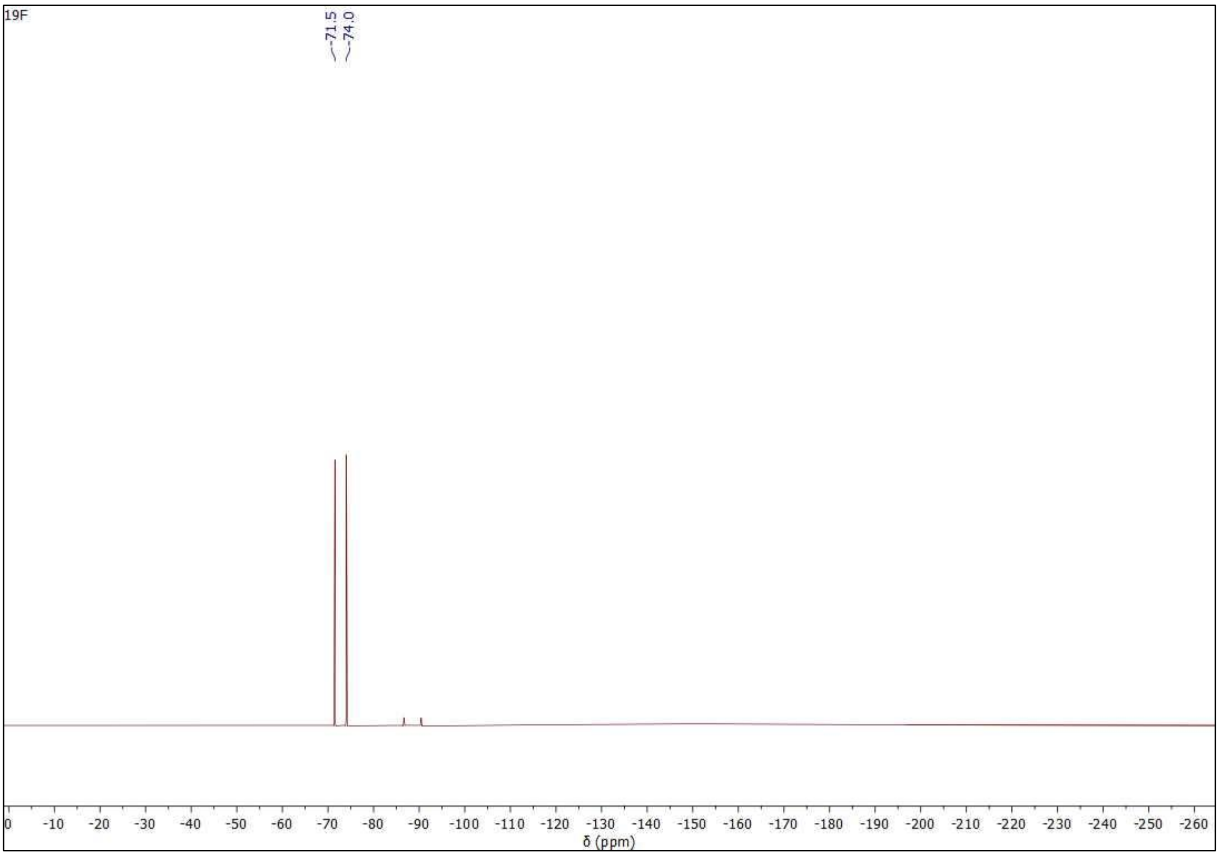

Figure S28. $^{19}F\{^{1}H\}$ NMR spectrum of **5b** in CD_2Cl_2.

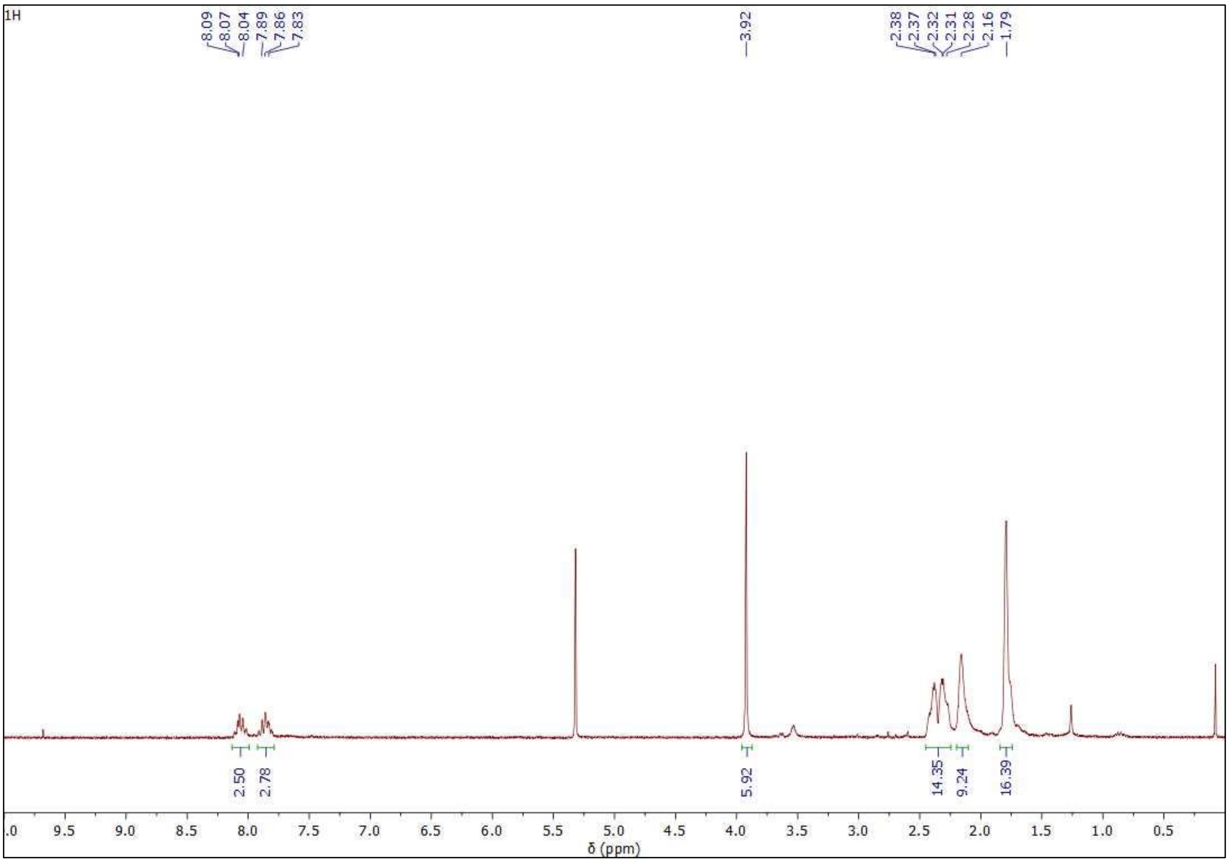

Figure S29. ^{1}H NMR spectrum of **5c** in CD_2Cl_2.

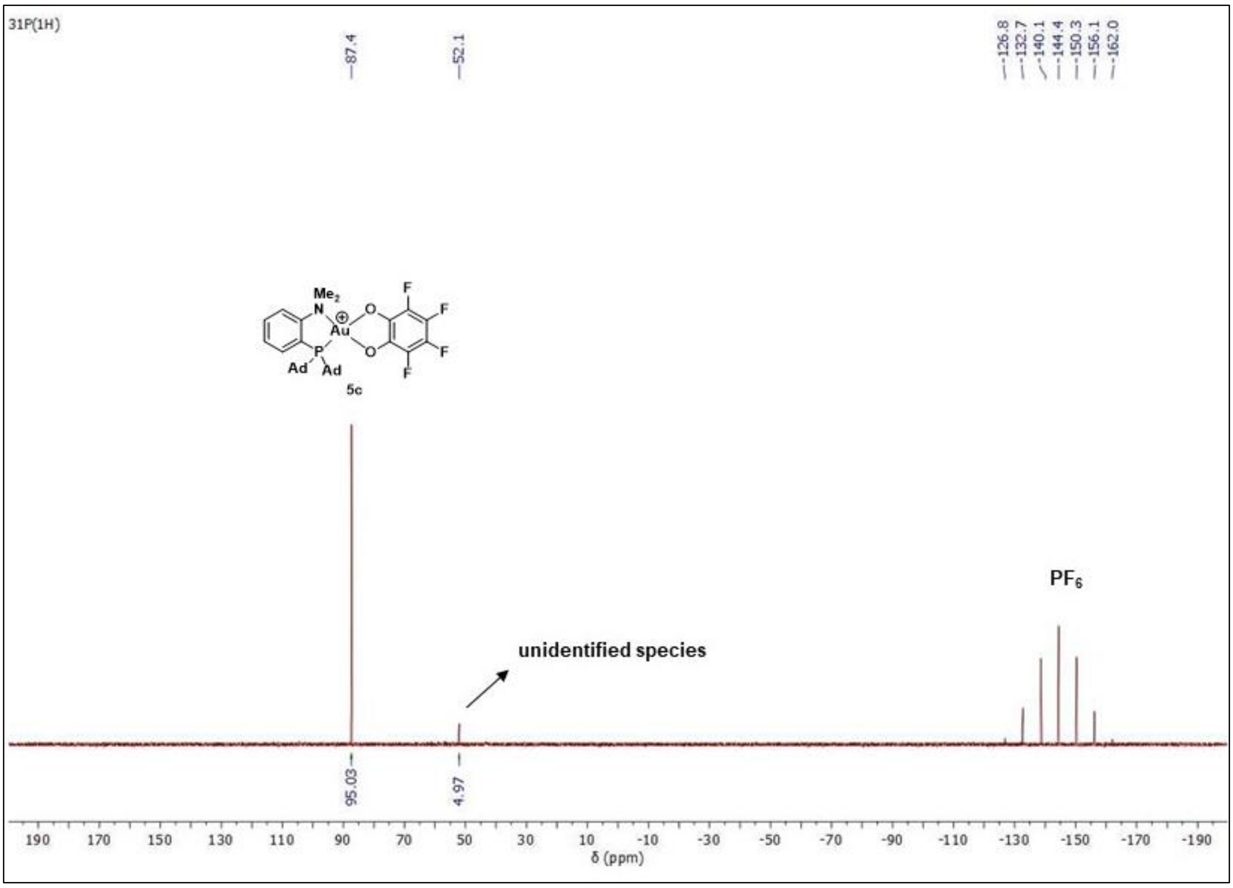

Figure S30. $^{31}P\{^{1}H\}$ NMR spectrum of **5c** in CD_2Cl_2.

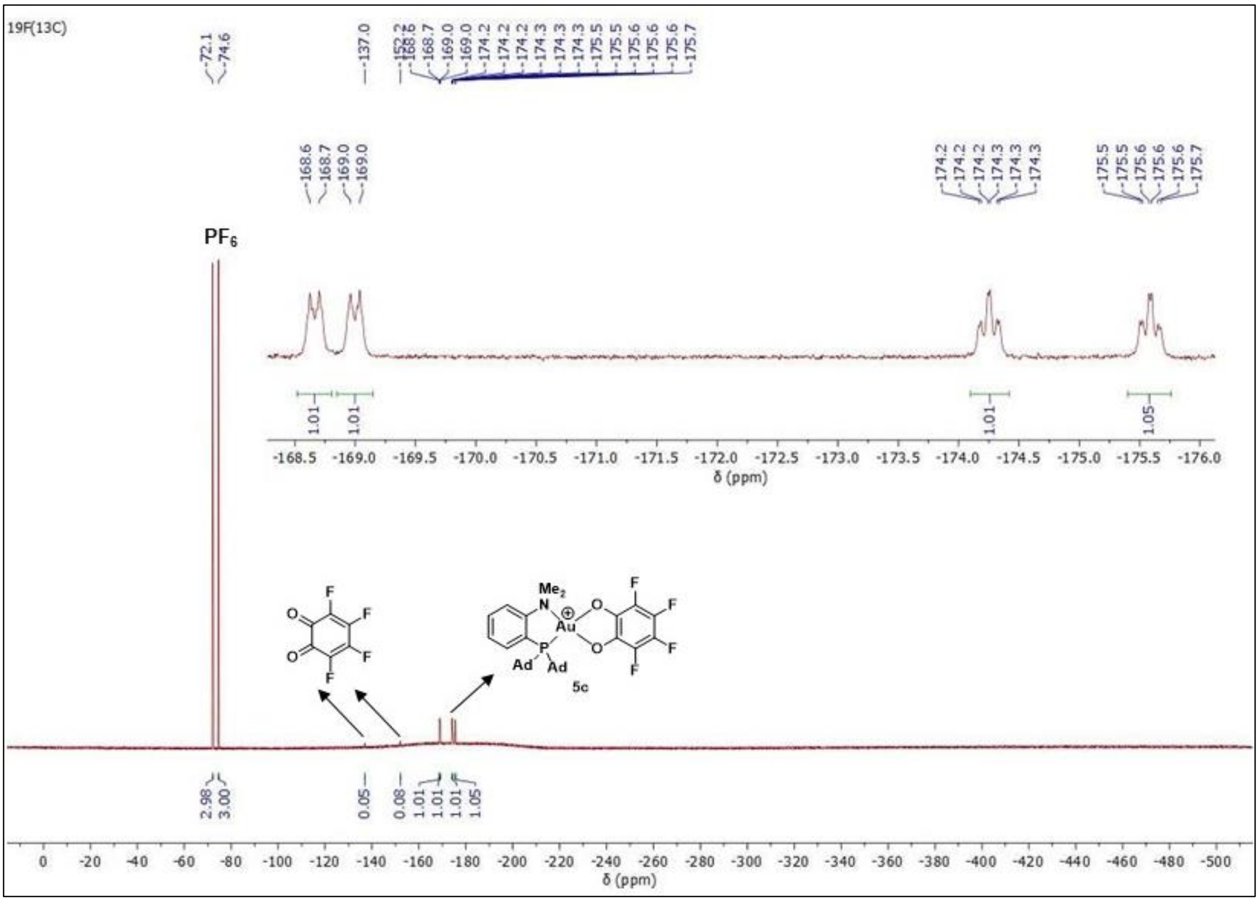

Figure S31. $^{19}F\{^1H\}$ NMR spectrum of **5c** in CD_2Cl_2.

3. Reactions of 5a

3.1 Reaction of **5a** with (nBu)$_4$NCl

To a solution of **5a** (5 mg, 0.005 mmol) CD_2Cl_2 (0.4 mL) a solution of (nBu)$_4$NCl in CD_2Cl_2 (100 µL, 1 equiv. 5 x 10^{-2}M) was added at rt. Upon addition the grey green solution became orange instantaniously. A ^{1}H and ^{31}P NMR spectrum was recorded before and after the addition. ^{13}C NMR was also recorded after the addition to confirm the release of *o*-chloranil.

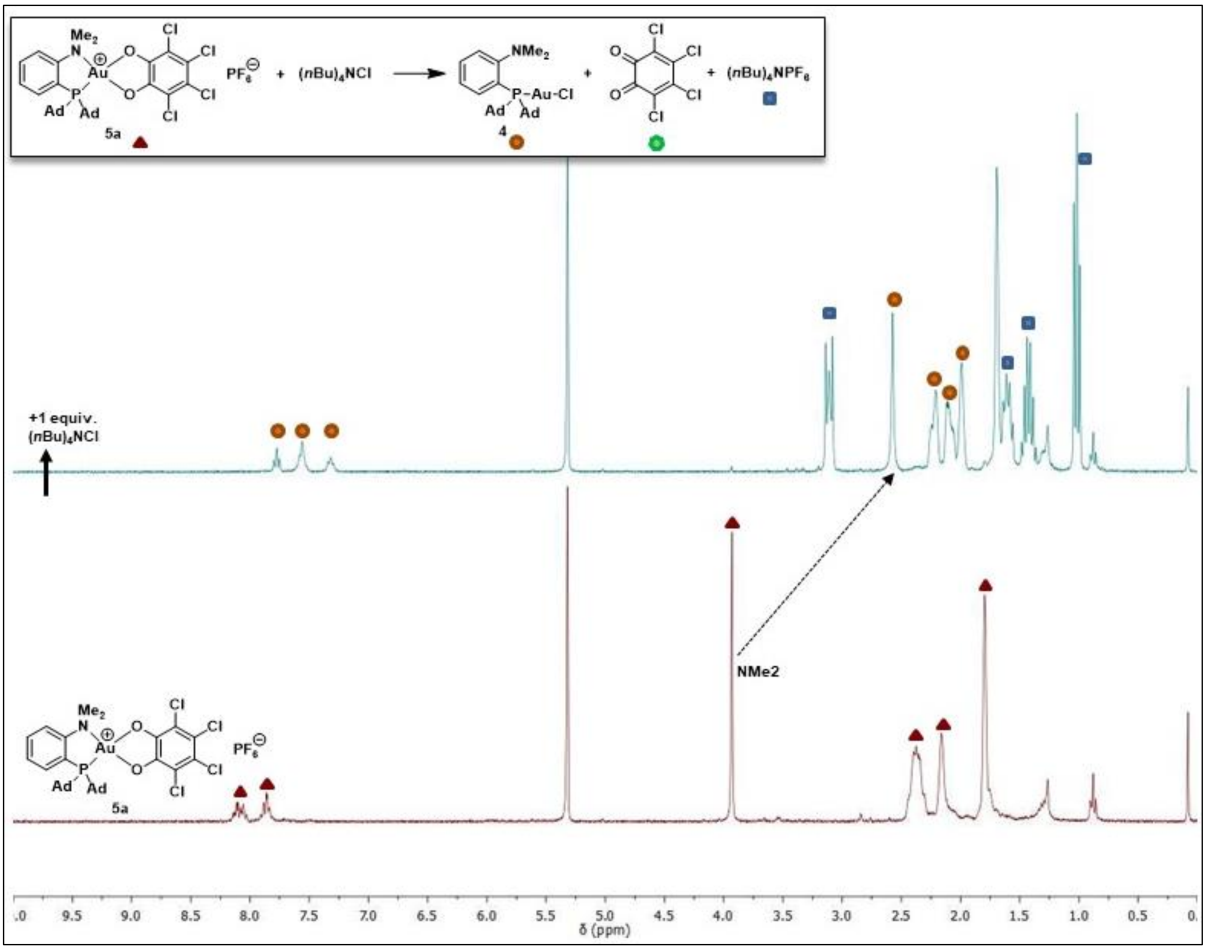

Figure S32. ^{1}H NMR spectrum of an equimolar mixture of **5a** and (nBu)$_4$NCl in CD_2Cl_2.

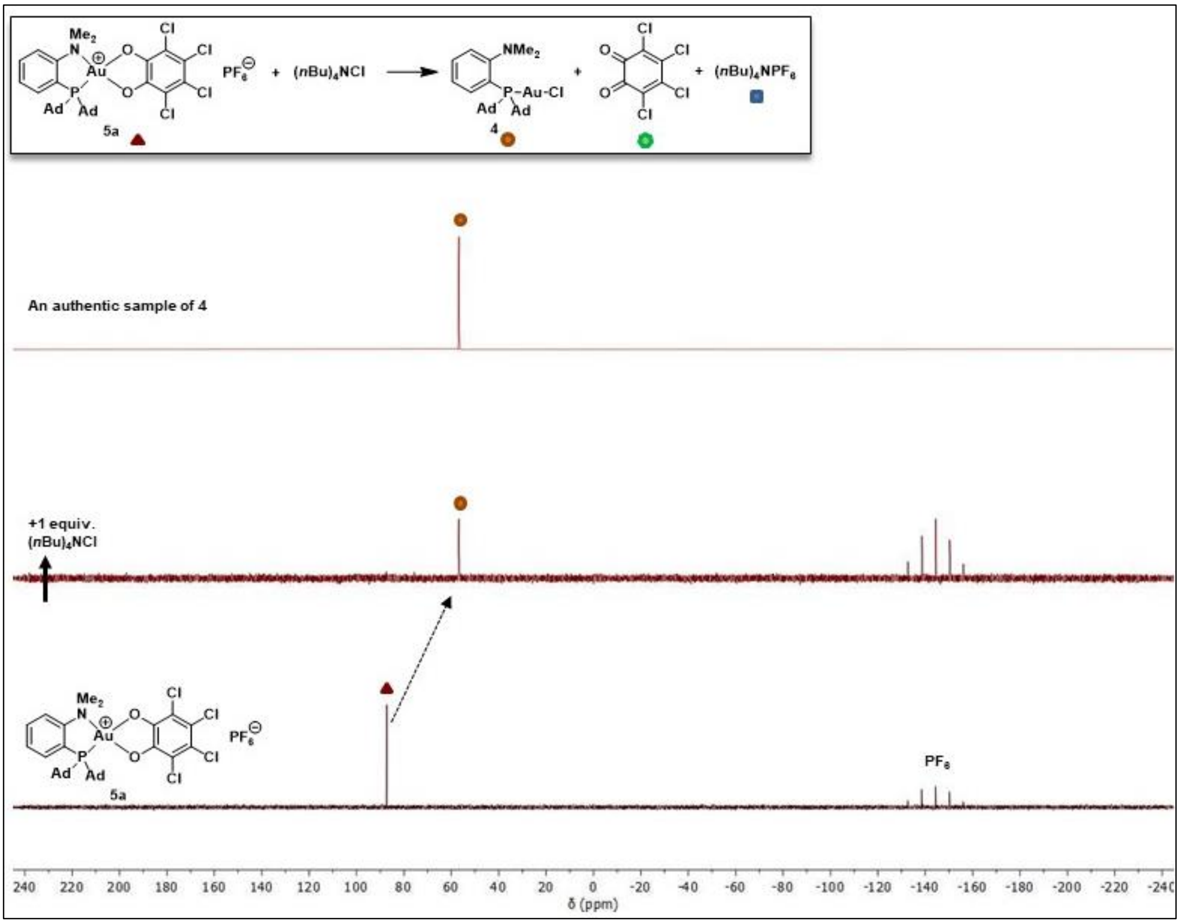

Figure S33. $^{31}P\{^{1}H\}$ NMR spectrum of an equimolar mixture of **5a** and (nBu)$_4$NCl in CD_2Cl_2.

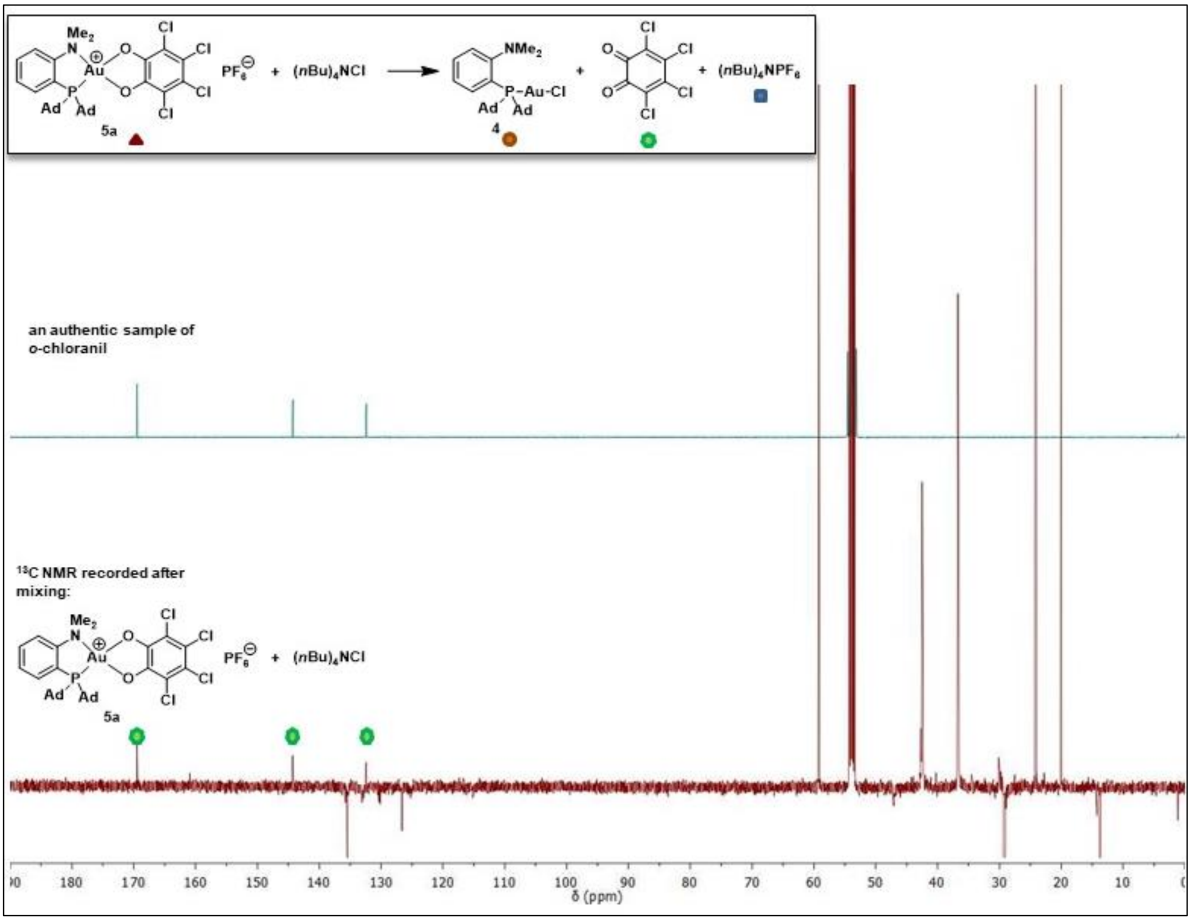

Figure S34. $^{13}C\{^1H\}$ NMR spectrum of an equimolar mixture of **5a** and $(n\text{Bu})_4$NCl in CD_2Cl_2.

3.2 Reaction of **5a** with ethylene

1. A solution of **5a** (5 mg, 0.005 mmol) CD_2Cl_2 (0.5 mL) was placed into a J Young NMR tube. ($^1H/^{31}P$ NMR was recorded).
2. Then the headspace was replaced with ethylene (1 bar). The tube was shaken during 2 minutes at rt, while the grey-green solution became gradually orange. ($^1H/^{31}P$ was recorded).
3. The solvent was removed under reduced pressure and the tube was left under vacuum for another 30 minutes at rt. The solid material was resolubilized (CD_2Cl_2). ($^1H/^{31}P$ NMR was recorded).

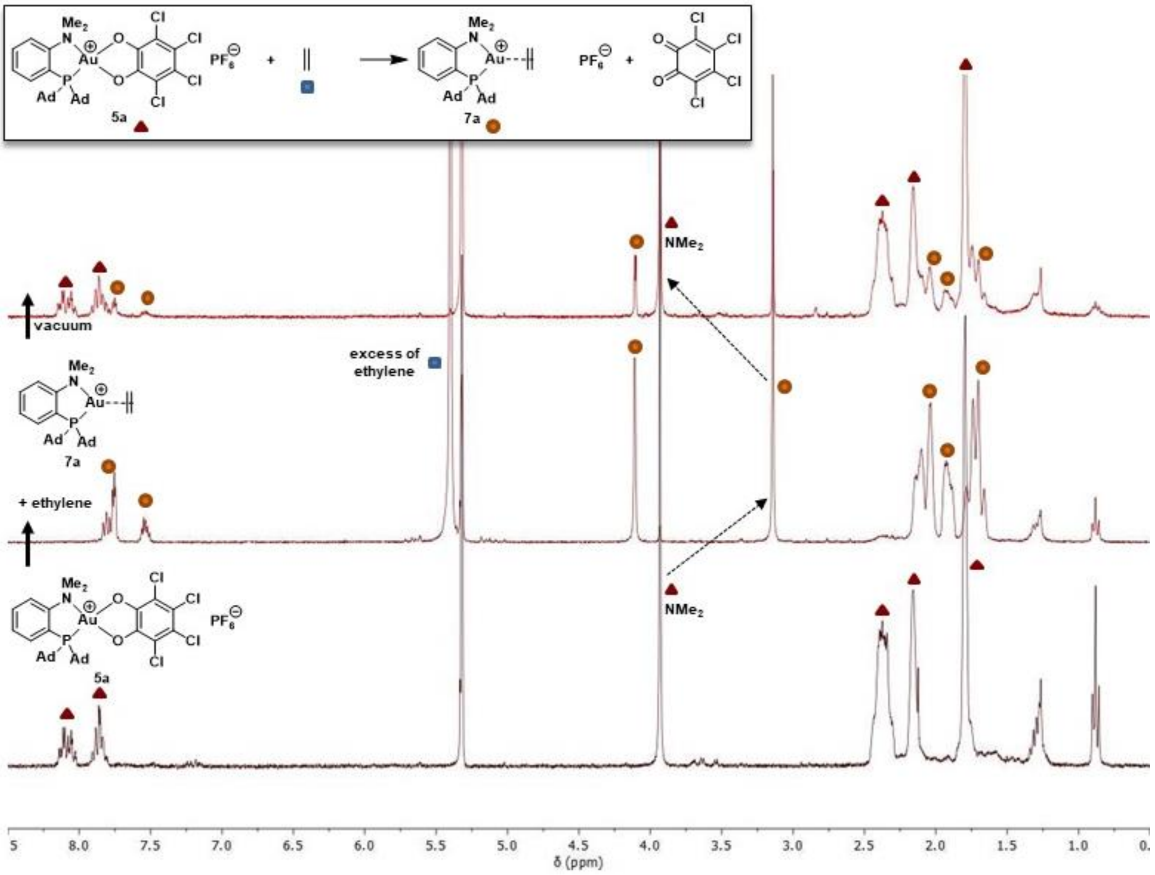

Figure S35. ^1H NMR spectrum of the reaction of **5a** with an excess of ethylene in CD_2Cl_2.

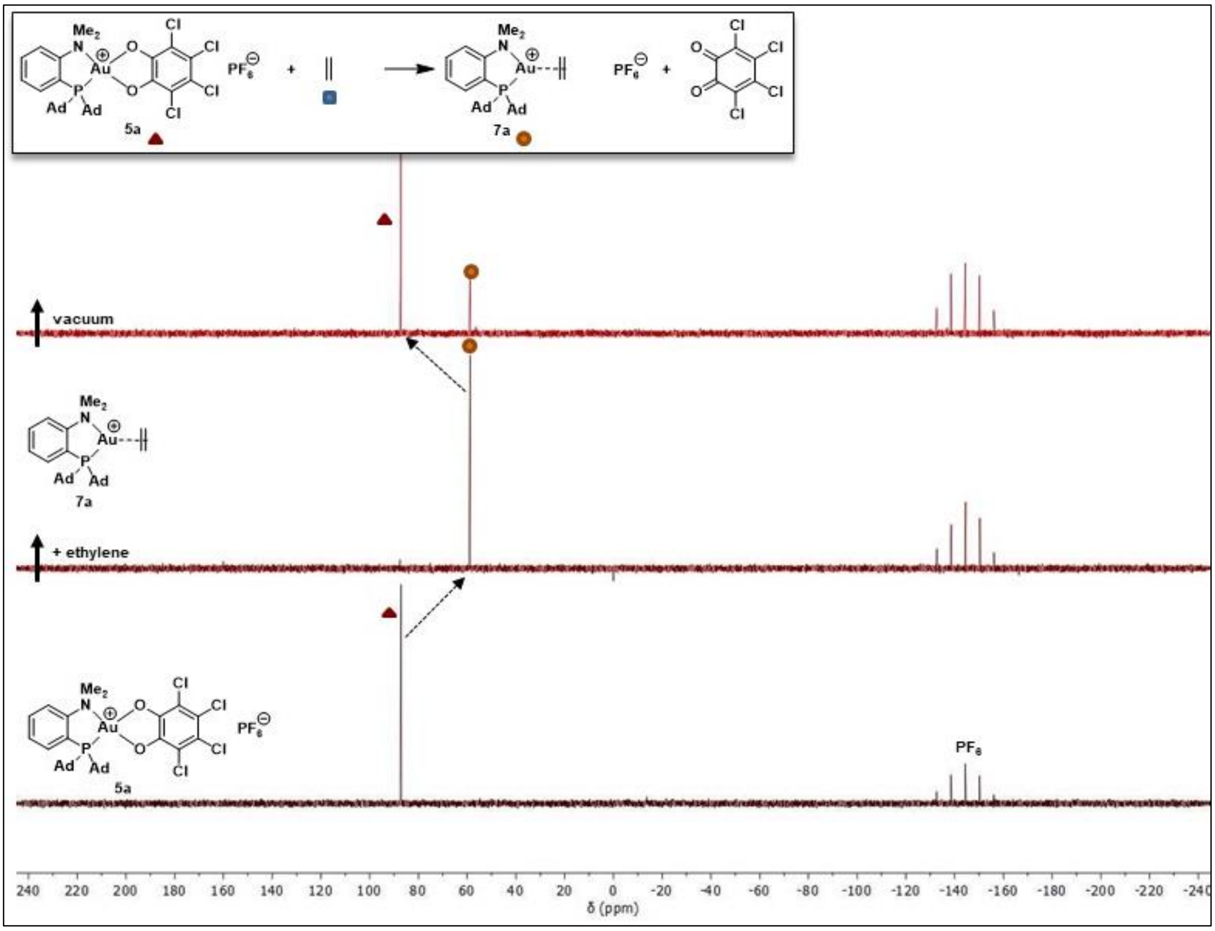

Figure S36. $^{31}P\{^1H\}$ NMR spectrum of the reaction of **5a** with an excess of ethylene in CD_2Cl_2.

3.3 Reaction of **5a** with styrene

1. A solution of **5a** (5 mg, 0.005 mmol) CD_2Cl_2 (0.4 mL) was placed into a J Young NMR tube. ($^1H/^{31}P$ NMR was recorded).
2. Then a solution of styrene in CD_2Cl_2 (5 x 10^{-2}M) was added at rt in increasing amounts. ($^1H/^{31}P$ NMR was recorded).

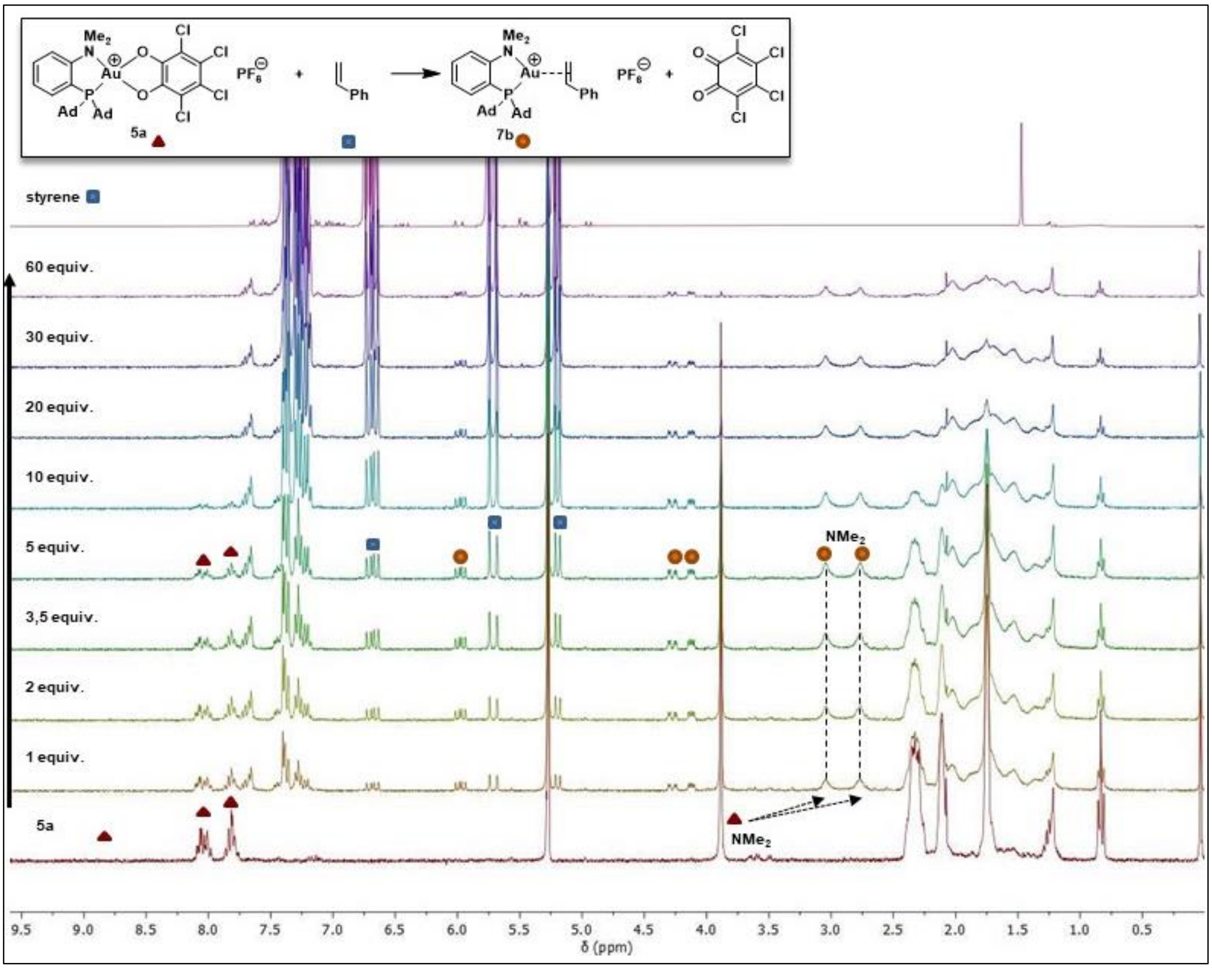

Figure S37. ^1H NMR spectra of the reaction of **5a** after the addition of increasing amounts of styrene in CD_2Cl_2.

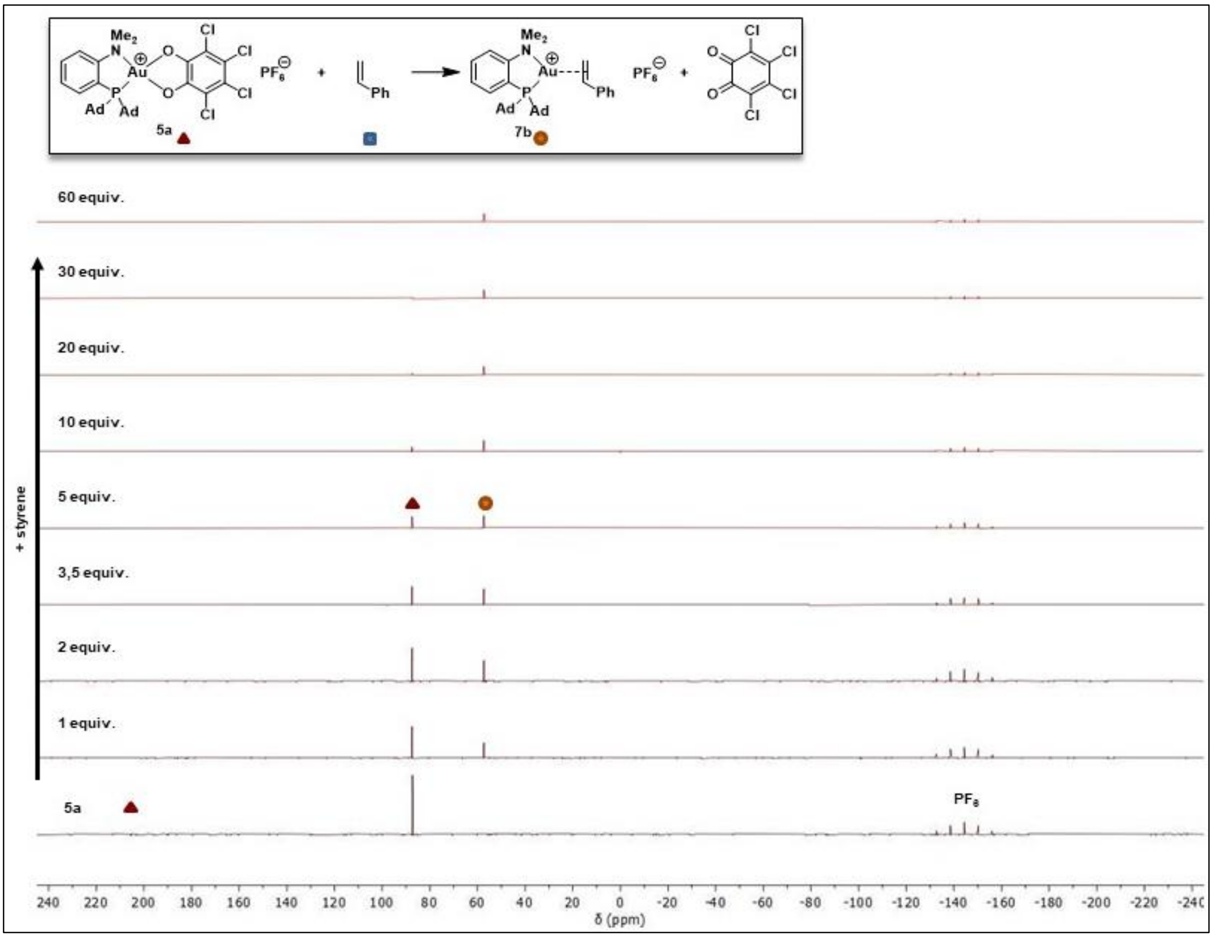

Figure S38. $^{31}P\{^{1}H\}$ NMR spectra of the reaction of **5a** after the addition of increasing amounts of styrene in CD_2Cl_2.

3.4 Reaction of **5a** with norbornene

1. A solution of **5a** (5 mg, 0.005 mmol) CD_2Cl_2 (0.4 mL) was placed into a J Young NMR tube. (^1H/^{31}P NMR was recorded).
2. Then a solution of norbornene in CD_2Cl_2 (100 µL, 1 equiv. 5 x 10^{-2}M) was added at rt. (^1H/^{31}P NMR was recorded).
3. **7c** was unambigously identified by using an authetic sample of **7c-SbF₆**. **7c-SbF₆** was prepared by treating **4** with $AgSbF_6$, then with norbornene.

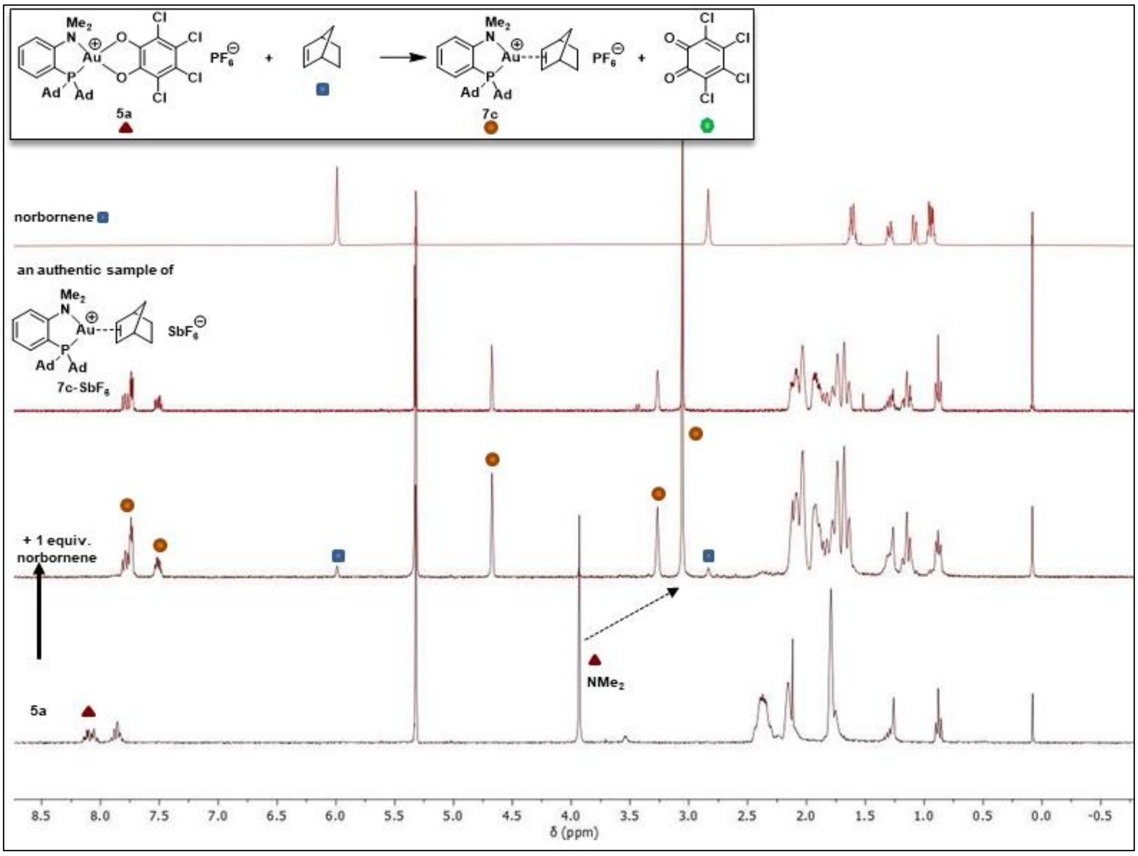

Figure S39. ^1H NMR spectrum of an equimolar mixture of **5a** and norbornene in CD_2Cl_2.

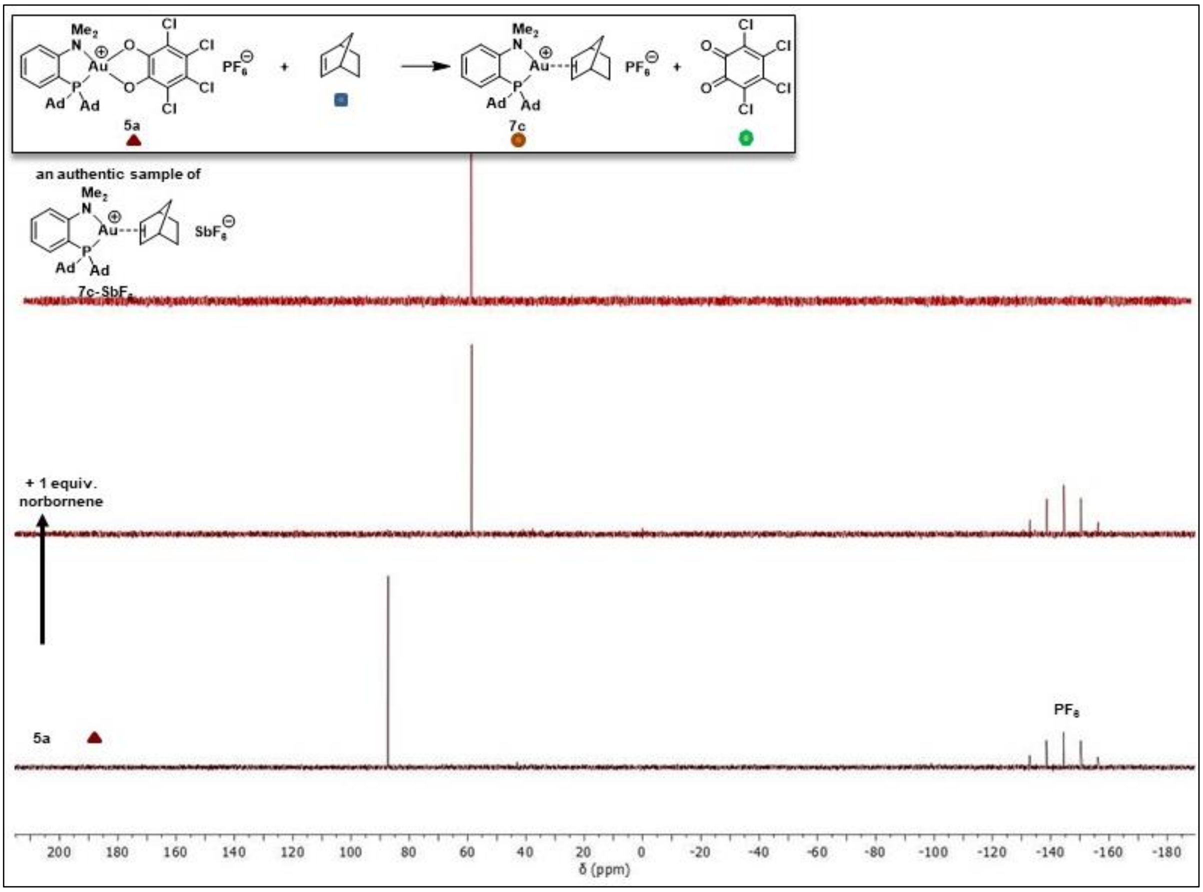

Figure S40. ^{31}P{^{1}H} NMR spectrum of an equimolar mixture of **5a** and norbornene in CD_2Cl_2.

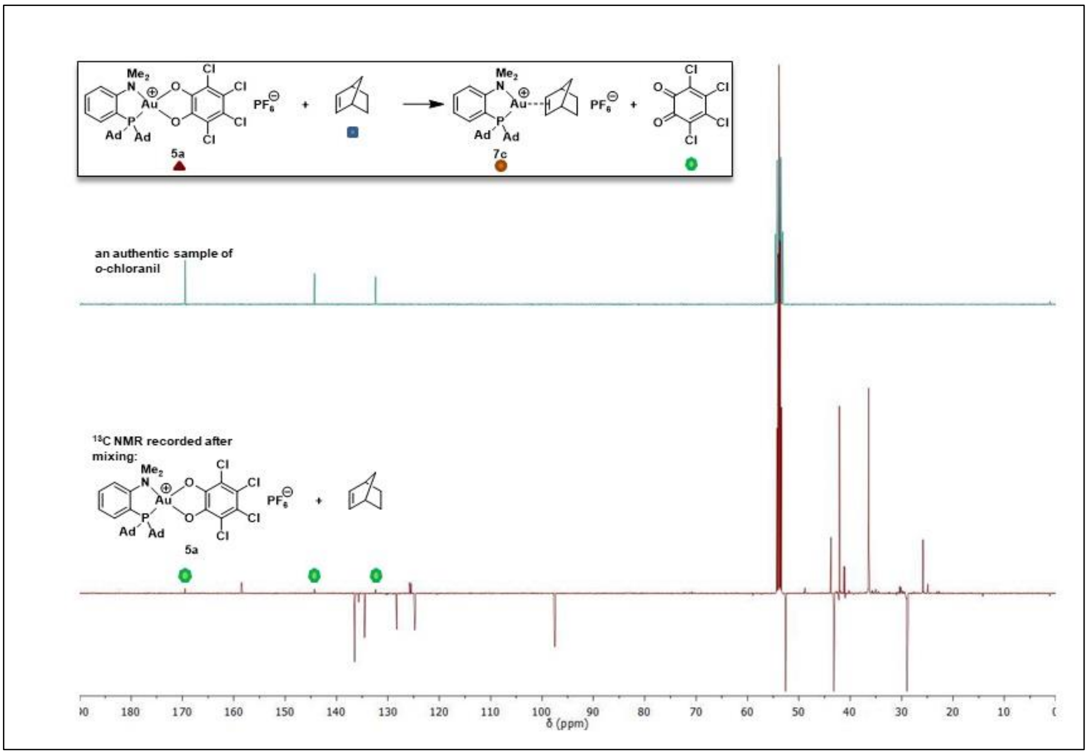

Figure S41. ^{13}C{^{1}H} NMR spectrum of an equimolar mixture of **5a** and norbornene in CD_2Cl_2.

4. Crystallographic data

Crystallographic data were collected at 193(2) K on a Bruker-AXS D8-Venture equipped with a PHOTON III detector and using MoK_{α} radiation (λ=0.71073 Å). Phi- and omega-scans were used. An empirical absorption correction was applied[4]. The structures were solved using an intrinsic phasing method (SHELXT)[5] and refined using the least-squares method on F^2 [6]. All non-H atoms were refined with anisotropic displacement parameters. Hydrogen atoms were refined isotropically at calculated positions using a riding model. For **3b**, as in the related structure of the Au(III) complex deriving from *nido*-*o*-carboranyl diphosphines, the four carborane open-face H atoms were located in difference Fourier maps and the H atom bridging two boron atoms was refined using the same B-H distance restraints as in the previous report by Laguna *et al.*[7] For **6c**, the SQUEEZE function of PLATON[8] was used to eliminate the contribution of the electron density in the final refinement of highly disordered solvent.

CCDC 2180464 (**3b**), 2180465 (**5a**), 2180466 (**5b**), 2180467 (**5c**) and 2180468 (**6c**) contain the supplementary crystallographic data for this paper. These data can be obtained free of charge from The Cambridge Crystallographic Data Centre via www.ccdc.cam.ac.uk/data_request/cif.

Table S1. Crystal Data, Data Collection, and Structure Refinement for **3b**, **5a-c** and **6c**.

	**3b**	**5a**	**5b**	**5c**	**6c**
formula	$C_{24}H_{46}AuB_9Br_4N_4O_2P_2$	$C_{34}H_{40}AuCl_4NO_2P$, SbF_6,0.5(CH_2Cl_2)	$C_{34}H_{40}AuBr_4NO_2P$, SbF_6,0.5(CH_2Cl_2)	$C_{34}H_{40}AuF_4NO_2P$, SbF_6	$C_{35}H_{50}AuNP$, SbF_6
M_r	1098.45	1142.62	1320.46	1034.35	948.46
crystal system	monoclinic	monoclinic	monoclinic	monoclinic	monoclinic
space group	$P\ 2_1/c$	$C\ 2/c$	$C\ 2/c$	$P\ 2_1/c$	$P\ 2_1/c$
a (Å)	21.4705(13)	34.1585(18)	34.5469(17)	17.2368(9)	12.1539(4)
b (Å)	18.6774(12)	9.9683(6)	10.0066(6)	11.7530(6)	16.3040(7)
c (Å)	19.3095(10)	22.6313(13)	22.7409(13)	18.2426(10)	18.9124(9)
α (°)	90	90	90	90	90
β (°)	90.509(2)	95.446(2)	95.0516(19)	109.910(2)	97.080(2)
γ (°)	90	90	90	90	90
V (Å^3)	7743.1(8)	7671.2(8)	7830.9(8)	3474.8(3)	3719.1(3)
Z	8	8	8	4	4
ρ_{calc} (g cm^{-3})	1.885	1.979	2.240	1.977	1.694
μ (mm^{-1})	8.041	4.978	8.686	15.209	4.764
F(000)	4224	4440	5016	2008	1864
crystal size (mm^3)	0.12x0.06x0.01	0.25x0.25x0.20	0.22x0.10x0.04	0.20x0.20x0.16	0.20x0.20x0.04
T/K	193(2)	193(2)	193(2)	193(2)	193(2)
measd reflns	227256	113874	116299	62938	238431
Unique reflns (Rint)	14181 (0.0988)	9564 (0.0368)	9776 (0.0426)	6860 (0.0624)	18925 (0.0444)
Data/restraints/parameters	14181/4/869	9564/0/467	9776/16/480	6860/225/517	18925/0/416
GOF on F^2	1.063	1.088	1.058	1.068	1.072
R_1[a] [I>2σ(I)]	0.0346	0.0211	0.0242	0.0350	0.0315
wR_2[b] [all data]	0.0793	0.0414	0.0505	0.0841	0.0635

[a] $R_1 = \Sigma||F_o| - |F_c||/ \Sigma |F_o|$. [b] $wR_2 = [\Sigma[w(F_o^2 - F_c^2)^2] / \Sigma [w(F_o^2)^2]]^{1/2}$.

5. X-ray absorption analyses

Samples were prepared as solid pellets in a cellulose matrix. Au L_3-edge data was acquired at cryogenic temperatures in transmission mode using liquid He cryostats available at the ALBA CLAESS beamline and ESRF BM23. Several XAS repeats were collected to ensure reproducibility and statistics Data processing was carried out with the Athena software package.[9] The energy scale was calibrated by setting the first inflection point of the Au foil spectra at 11919 eV. EXAFS were extracted using the autobk algorithm employing a spline in the 0 to 20 Å^{-1} region of k-space having an R_{bkg} of 1. The FEFF6 code was used for scattering path generation, and multi (k^1, k^2, k^3)-weighted fits of the data were carried out in r-space over an r-range of 1.0 to 2.5 Å and a k-range of 3-17 Å^{-1}.[10] The S_0^2 value was set to 0.9, and a global E_0 was employed with the initial E_0 value set to the first inflection point of the rising edge. Single scattering paths were fit in terms of a Δr_{eff} and σ^2,. To assess the goodness of the fits both the R_{factor} (%R) and the reduced χ_ν^2 (χ_ν^2) were minimized, ensuring that the data was not over-fit.[11,12] An increase in the number of variables is generally expected to improve the Rf_{actor},however χ_ν^2 may go through a minimum then increase, which is an indication that the model is over-fitting the data. Best fit models were determined using a grid search with fixed values for path coordination numbers (N) by employing *larch*, the Python implementation of Artemis.[13]

Figure S42. EXAFS fits of **5a**. Multi (k^1, k^2, k^3)-weighted fits carried out in r-space (1-2.5 Å) over a k-range of 3-17 $Å^{-1}$ using a Hanning window (dk 1), and $S_0 = 0.9$. Bond distances and disorder parameters (Δr_{eff} and σ^2) were allowed to float having initial values of 0.0 Å and 0.003 $Å^2$ respectively, with a universal E_0 and $\Delta E_0 = 0$ eV. Best model fits are highlighted in bold and σ^2 values reported as (x10^3).

**5a**								
	R_{FACTOR}	**0.014**	0.023	0.054	0.016	0.017	0.025	0.015
	X^2_v	**799**	1288	2990	893	947	1559	934
	Var. No.	**4**	4	4	4	4	5	5
	ΔE_0	**6.8(1.0)**	6.5(1.2)	5.0(1.9)	7.7(1.0)	6.9(1.0)	5.2(1.3)	5.5(1.1)
	N	**1.5**	3.0	2.0	1.5	2.0	2.0	2.5
**M - O/N - M**	**r**	**1.96(0.01)**	2.01(0.01)	1.99(0.02)	1.96(0.01)	1.98(0.01)	1.98(0.01)	1.99(0.01)
	σ^2	**1.4(0.5)**	3.0(0.7)	1.0(0.9)	2.8(0.6)	1.6(0.5)	1.2(0.9)	2.9(0.8)
	N	**1.5**	-	-	2.0	1.0	-	-
**M - O/N - M**	**r**	**2.05(0.01)**	-	-	2.06(0.01)	2.07(0.01)	-	-
	σ^2	**1.4(0.5)**	-	-	2.8(0.6)	1.6(0.5)	-	-
	N	**1.0**	1.0	1.0	1.0	1.0	-	-
**M - P/Cl - M**	**r**	**2.27(0.01)**	2.27(0.02)	2.27(0.02)	2.26(0.01)	2.27(0.01)	-	-
	σ^2	**1.4(0.5)**	3.0(0.7)	1.0(0.9)	2.8(0.6)	1.6(0.5)	-	-
	N	-	-	-	-	-	1.0	1.0
**M -Cl/P - M**	**r**	-	-	-	-	-	2.22(0.01)	2.22(0.01)
	σ^2	-	-	-	-	-	1.2(0.9)	1.8(0.8)

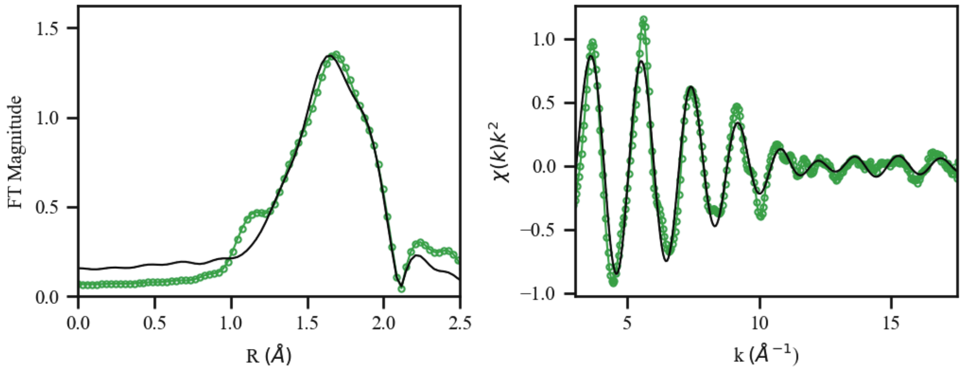

Figure S43. EXAFS fits of **MeDalPhosAuCl** (**4**). Multi (k^1, k^2, k^3)-weighted fits carried out in r-space (1-2.5 Å) over a k-range of 3-17 Å^{-1} using a Hanning window (dk 1), and $S_0 = 0.9$. Bond distances and disorder parameters (Δr_{eff} and σ^2) were allowed to float having initial values of 0.0 Å and 0.003 Å^2 respectively, with a universal E_0 and $\Delta E_0 = 0$ eV. Best model fits are highlighted in bold and σ^2 values reported as (x10^3).

**4**								
	R_{FACTOR}	0.297	0.124	0.237	0.063	**0.032**	0.033	0.036
	X^2_v	4367	1826	3485	1026	**577**	595	731
	Var. No.	3	3	3	4	**5**	5	6
	ΔE_0	22.4(4.4)	8.9(2.7)	4.9(5.1)	13.8(1.8)	**7.2(1.9)**	14.3(1.8)	10.8(1.9)
**M - O/N - M**	N	1.0	-	-	1.0	-	1.0	1.0
	**r**	2.14(0.05)	-	-	2.10(0.02)	-	2.10(0.03)	2.13(0.05)
	σ^2	1.0(0.1)	-	-	1.0(0.0)	-	4.1(3.0)	6.0(9.4)
**M - O/N - M**	N	-	-	-	-	-	-	-
	**r**	-	-	-	-	-	-	-
	σ^2	-	-	-	-	-	-	-
**M - P/Cl - M**	N	-	-	1.0	1.0	**1.0**	-	1.0
	**r**	-	-	2.29(0.03)	2.33(0.01)	**2.30(0.07)**	-	2.26(0.09)
	σ^2	-	-	1.0(1.8)	1.0(0.0)	**5.0(7.0)**	-	6.0(9.4)
**M -Cl/P - M**	N	-	1.0	-	-	**1.0**	1.0	1.0
	**r**	-	2.26(0.02)	-	-	**2.27(0.03)**	2.29(0.01)	2.29(0.05)
	σ^2	-	1.0(0.3)	-	-	**2.8(3.3)**	1.8(0.9)	2.3(2.2)

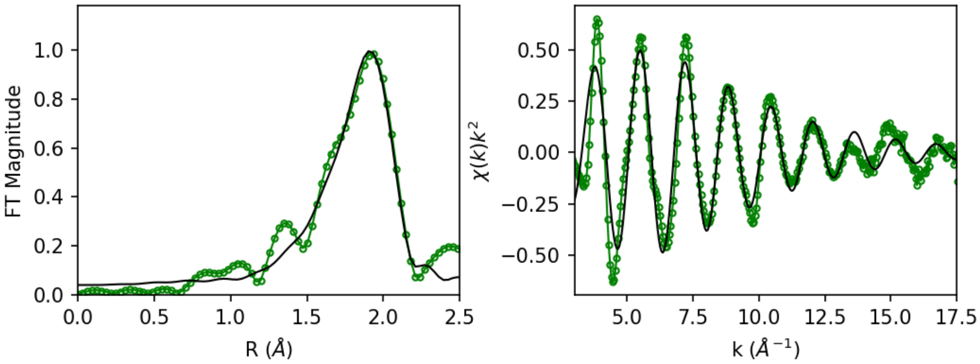

6. Computational details

All calculations were performed with the Gaussian 16 package[14] with the B3PW91[15] hybrid functional and D3 dispersion correction of Grimme with Becke–Johnson damping (DFT-D3*(*BJ)),[16] by taking into account solvent effect (Dichloromethane : DCM) by means of the polarizable continuum model PCM[17] on real systems. The gold atom was described with the relativistic electron core potential SDD and associated basis set,[18] augmented by a set of f-orbital polarization functions.[19] The 6-31G** basis set were employed for all other atoms. All stationary points involved were fully optimized by taking into account solvent effect and dispersion. Frequency calculations were undertaken to confirm the nature of the stationary points, yielding one imaginary frequency for transition states (TS) and all of them positive for *minima*. The connectivity of the transition state **TS1** and their adjacent *minima* was confirmed by intrinsic reaction coordinate (IRC)[20] calculations.

To have better insights on the process and know when Au to *o*-quinone electron transfer occurs, the Potential Energy Surface (PES) was scrutinized, from **TS1** to **5a**, by scanning the reaction coordinate $O_{cistoP}AuPC_{Ph}$ degree by degree. Due to the large number of points to be calculated, we performed this scan at B3PW91-D3(BJ)/SDD+f(Au),6-31G**(C,H,N,O,P,Cl) level of theory by removing solvent effect. Then, Intrinsic Bond Orbitals (IBO) analysis was carried out on the main points of the PES. To do this, the calculations of wave functions have been made with version 7.4.2 of Turbomole[21] at B3LYP-D3(BJ)/def2-TZVP level of theory on the main points of the scan optimized at B3LYP-D3(BJ)/SDD+f(Au), 6-31G(d,p) level of theory by using Gaussian 16. Orbital visualizations were produced with IboView (v20150427),[22] program developed to analyze molecular electronic structure, based on Intrinsic Atomic Orbitals (IAOs).

Electronic configuration of all structures along the path, from **TS1** to **5a**, was determined using Natural Bond Orbital[23] analyses (NBO). These calculations were performed with NBO, 5.9 version.[24] For this purpose, the NBO orbitals associated with the d-orbitals of gold have been analyzed in detail as well as their occupancy.

The ^{13}C, ^{1}H, ^{31}P NMR chemical shifts (δ in ppm) were computed at PCM(DCM)-B3PW91-D3(BJ) level by taking into account solvent effects (DCM), employing the direct implementation of the Gauge Including Atomic Orbitals (GIAO),[25] with the IGLOII[26] basis set on C, H, O, N, Cl and P atoms, SDD+f on Au and using as reference $SiMe_4$ or H_3PO_4 optimized at the same level of theory, for respectively H, C or P atoms.

Figure S44. Optimized geometries and frontier orbitals of the Au(I) *o*-benzoquinone (**5'a-H**, left) and Au(III) catecholate (**5a-H**, right) valence isomers of the (P^N)Au(O^O)H_4^+ complex, computed at PCM(DCM)-B3PW91-D3(BJ)/SDD+f(Au),6-31G**(C,H,N,O,P). Distances in Å. Plot of the frontier orbitals with cutoff : 0.05. Hydrogen atoms have been omitted for clarity. Relative stability (ΔG in kcal/mol).

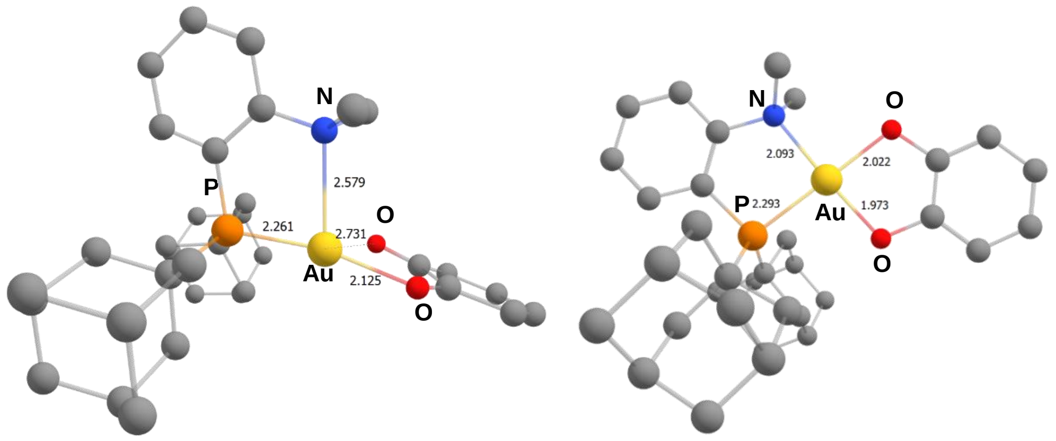

5'a-H
(+ 1.1 kcal/mol)

5a-H
(0)

LUMO **LUMO**

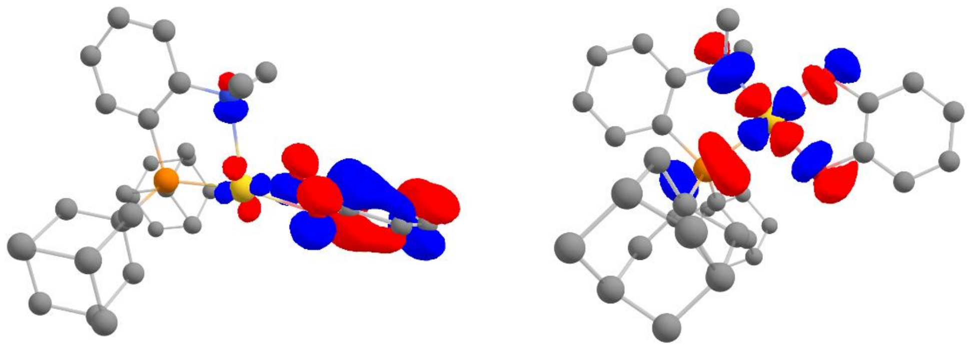

HOMO **HOMO**

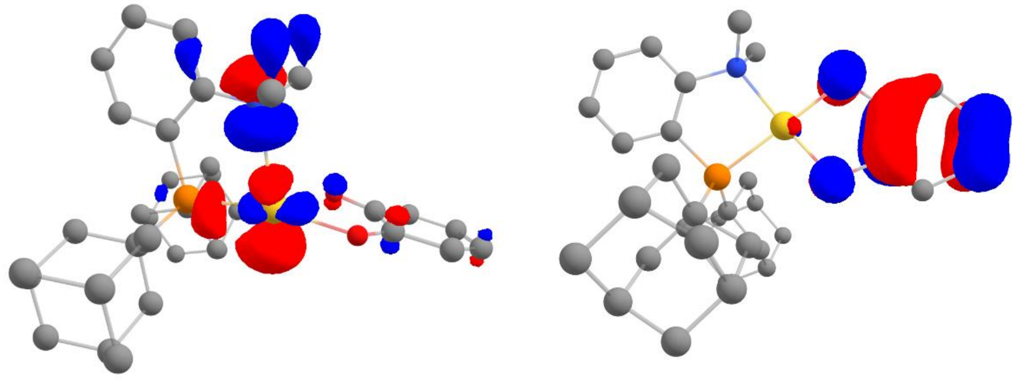

Table S2. Computed NMR chemical shifts (^{1}H, ^{13}C, ^{31}P) for **5a** and **5’a** at PCM(DCM)-B3PW91-D3(BJ)/SDD+f(Au),IGLO-II(C,H,N,O,P,Cl).

		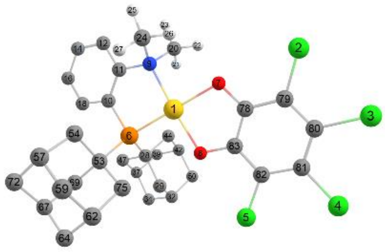	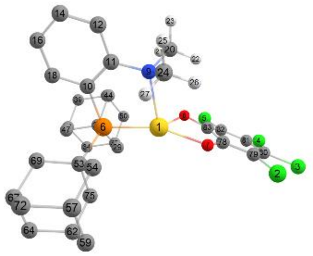
		**5a**	**5’a**
**Amino group**	C_{20}	60.7	57.0
	H_{21}	3.3	3.1
	H_{22}	4.3	2.9
	H_{23}	3.7	3.1
	C_{24}	60.4	56.9
	H_{25}	3.6	3.1
	H_{26}	4.1	3.3
	H_{27}	3.4	3.2
**Catecholate *o*-Quinone**	C_{78}	156.0	170.2
	C_{79}	128.0	140.4
	C_{80}	134.4	155.8
	C_{81}	133.5	155.3
	C_{82}	128.2	141.1
	C_{83}	158.9	173.1
**Phosphine**	P_{6}	90.8	66.0

Figure S45. Optimized geometry and frontier orbitals of **TS1**, computed at PCM(DCM)-B3PW91-D3(BJ)/SDD+f(Au),6-31G**(H,C,N,O,Cl,P). Distances in Å.

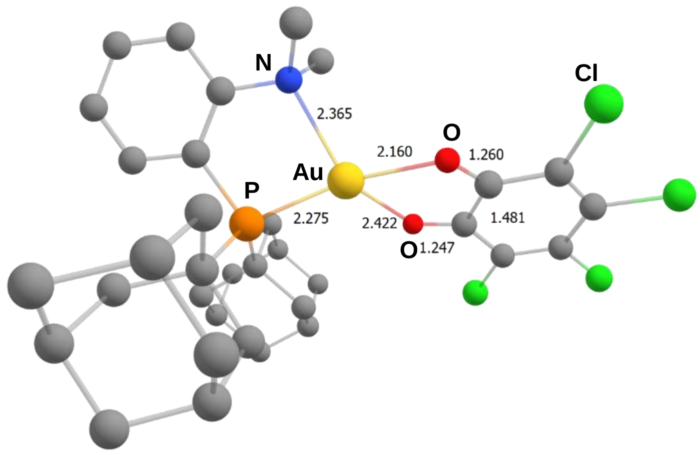

TS1

LUMO **HOMO**

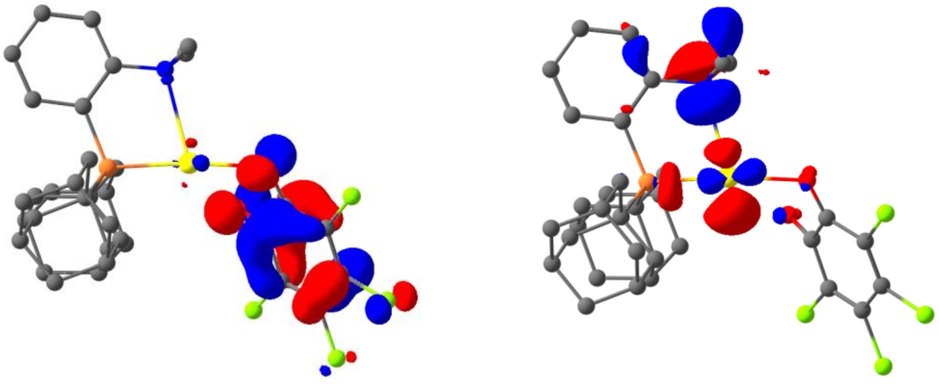

Figure S46. IBO analysis along the reaction path, from **TS1** to **5a**, the reaction coordinate being the bond dihedral angle $O_{cistoP}AuPC_{Ph}$ (D in °). Scan of the Potential Energy Surface carried out at B3PW91-D3(BJ)/SDD+f(Au),6-31G** (H,C,N,O,P,Cl) level without solvent effect. Evolution of the main IBO orbitals located on gold and o-quinone/catecholate moieties. Electron configuration from NBO calculations, by analyzing d NBO orbitals. The main orbital evolutions are framed in red. [&]**TS1** and **5a** were optimized at PCM(DCM)-B3PW91-D3(BJ)/SDD+f(Au),6-31G** (H,C,N,O,P,Cl) level.

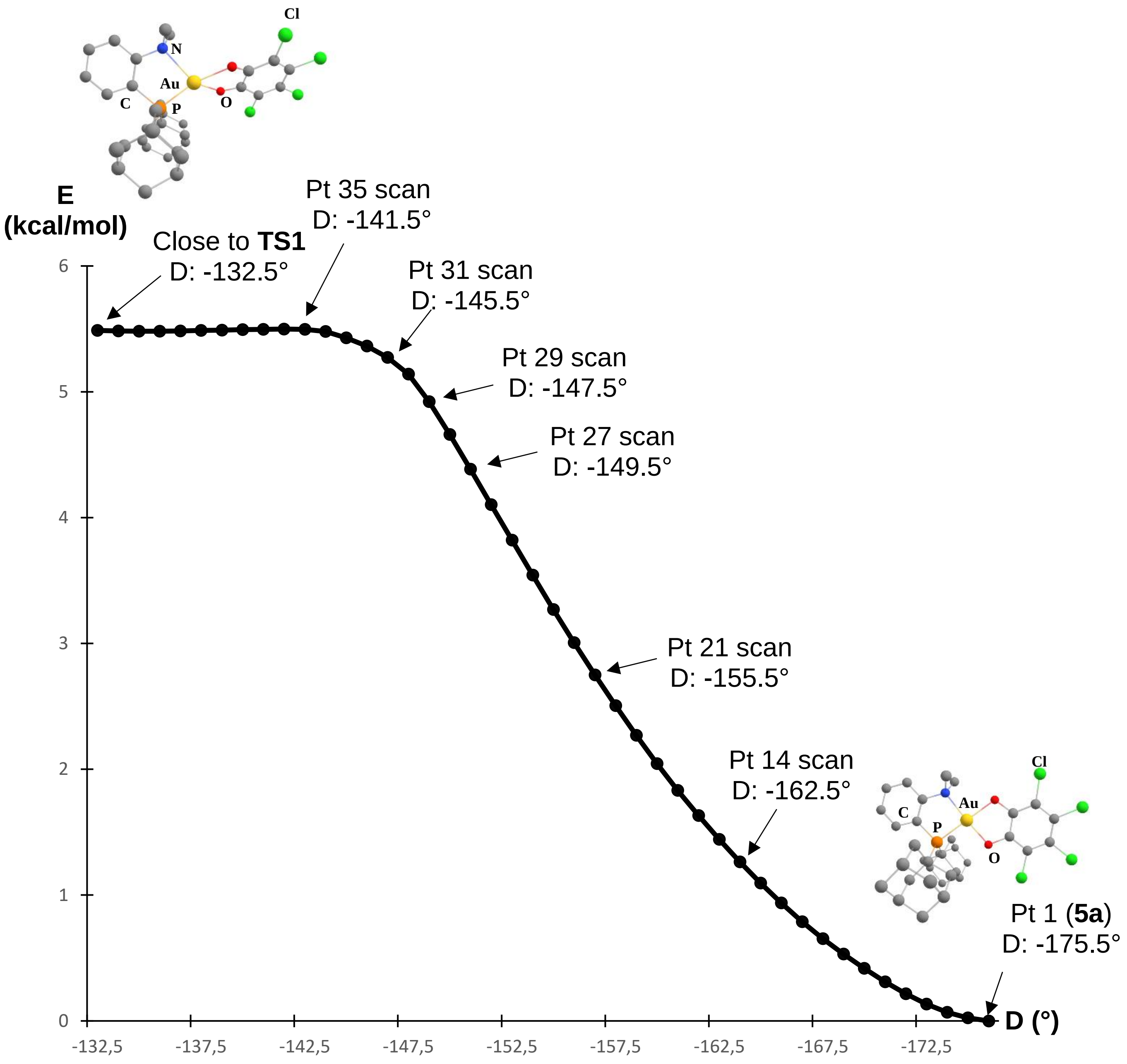

	dxz	dx2-y2
**5a[&]**<br>**D : -175.5°**<br>**NBO: d^{8}**	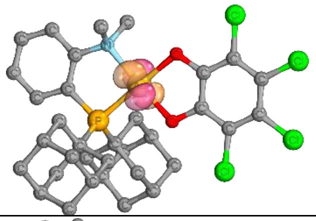	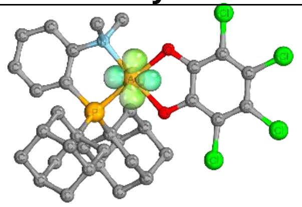
**Pt 14 scan**<br>**D : -162.5°**<br>**NBO: d^{8}**	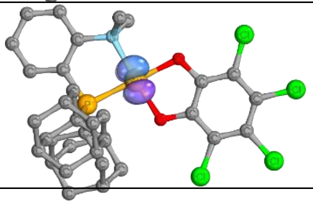	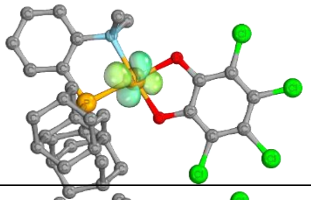
**Pt 21 scan**<br>**D : -155.5°**<br>**NBO: d^{8}**	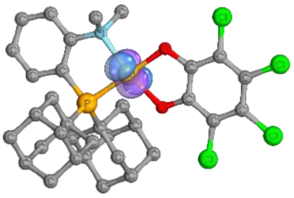	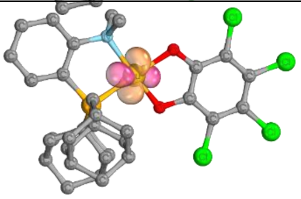
**Pt 27 scan**<br>**D : -149.5°**<br>**NBO: d^{8}**	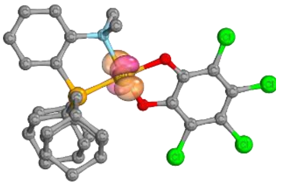	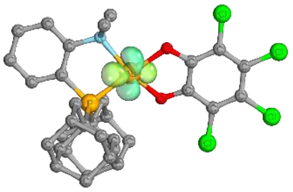
**Pt 29 scan**<br>**D : -147.5°**<br>**NBO: d^{9}**	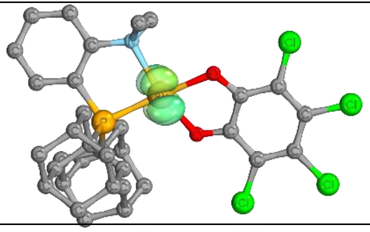	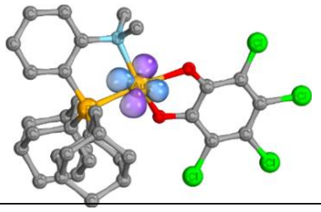
**Pt 31 scan**<br>**D : -145.5°**<br>**NBO: d$^{9.6}$**	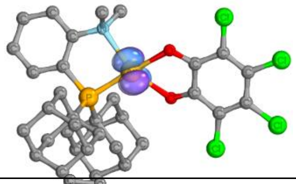	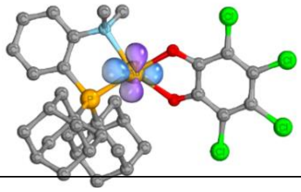
**Pt 35 scan**<br>**D : -141.5°**<br>**NBO: d$^{9.7}$**	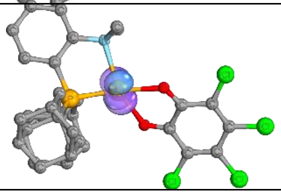	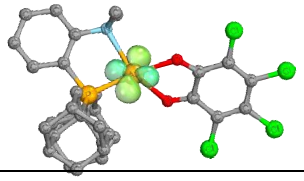
**TS1[&]**<br>**D : -126.9**<br>**NBO: d$^{9.7}$**	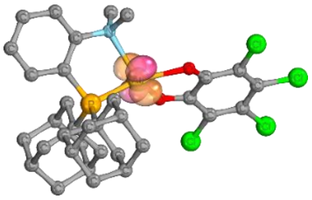	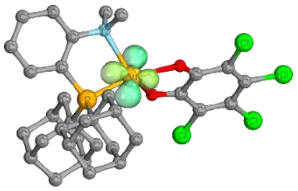

	dyz orbital	dz2 orbital
**5a[&]**<br>**D : -175.5°**<br>**NBO: d^{8}**	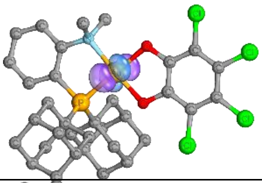	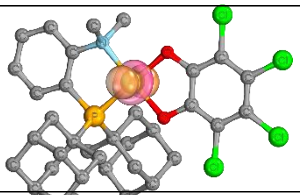
**Pt 14 scan**<br>**D : -162.5°**<br>**NBO: d^{8}**	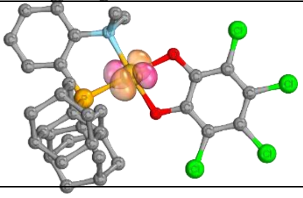	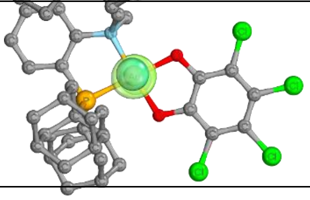
**Pt 21 scan**<br>**D : -155.5°**<br>**NBO: d^{8}**	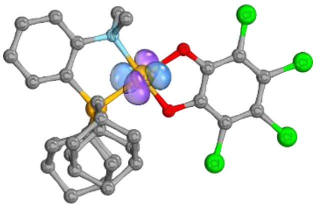	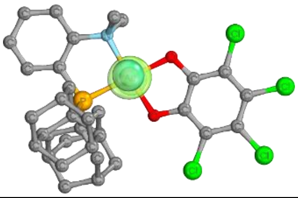
**Pt 27 scan**<br>**D : -149.5°**<br>**NBO: d^{8}**	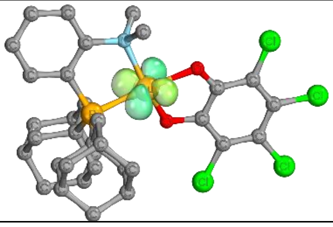	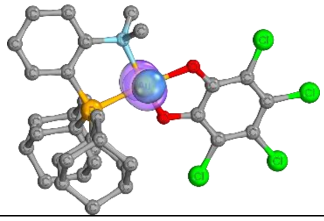
**Pt 29 scan**<br>**D : -147.5°**<br>**NBO: d^{9}**	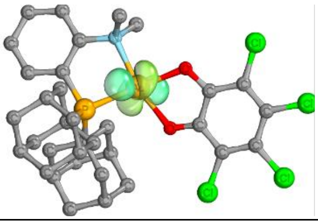	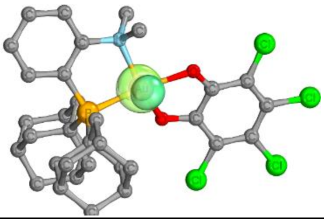
**Pt 31 scan**<br>**D : -145.5°**<br>**NBO: $d^{9.6}$**	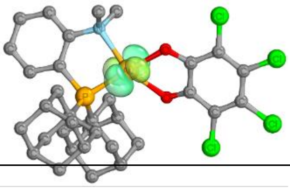	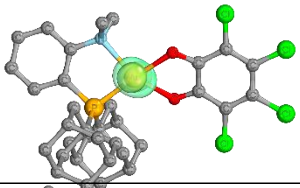
**Pt 35 scan**<br>**D : -141.5°**<br>**NBO: $d^{9.7}$**	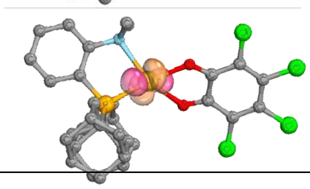	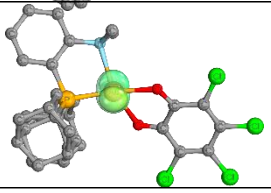
**TS1[&]**<br>**D : -126.9**<br>**NBO: $d^{9.7}$**	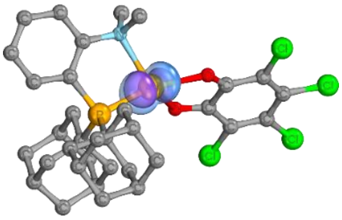	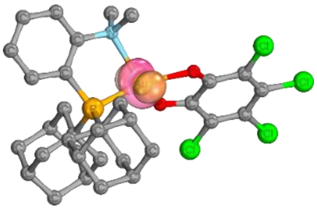

	dxy orbital	
5a[&]<br>D : -175.5°<br>NBO: d^8	/	
Pt 14 scan<br>D : -162.5°<br>NBO: d^8	/	
Pt 21 scan<br>D : -155.5°<br>NBO: d^8	apparition of a new<br>d orbital : $d^8 \rightarrow d^{10}$<br>(cf HOMO Au(III))	
Pt 27 scan<br>D : -149.5°<br>NBO: d^8	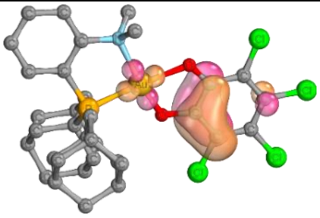	
Pt 29 scan<br>D : -147.5°<br>NBO: d^9	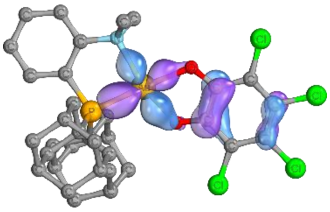	
Pt 31 scan<br>D : -145.5°<br>NBO: $d^{9.6}$	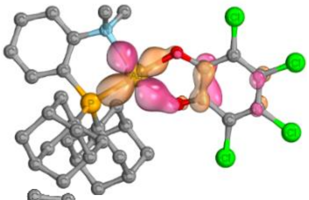	
Pt 35 scan<br>D : -141.5°<br>NBO: $d^{9.7}$	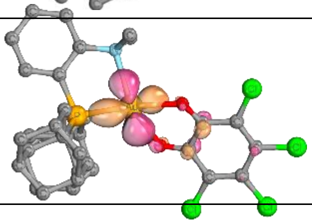	
TS1[&]<br>D : -126.9<br>NBO: $d^{9.7}$	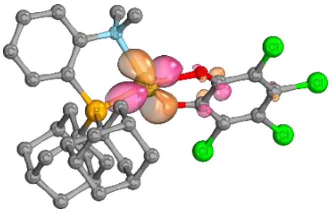	

	N→Au	P→Au
**5a[&]**<br>**D : -175.5°**<br>**NBO: d^{8}**	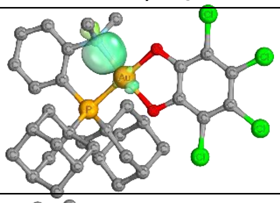	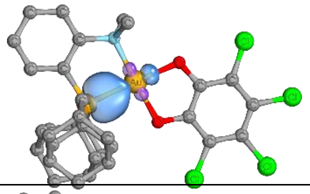
**Pt 14 scan**<br>**D : -162.5°**<br>**NBO: d^{8}**	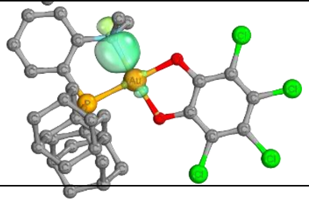	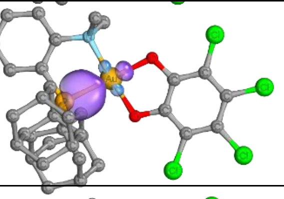
**Pt 21 scan**<br>**D : -155.5°**<br>**NBO: d^{8}**	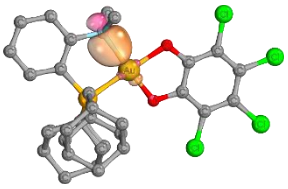	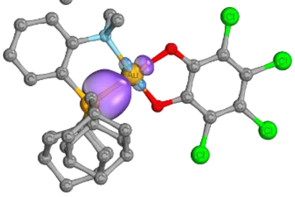
**Pt 27 scan**<br>**D : -149.5°**<br>**NBO: d^{8}**	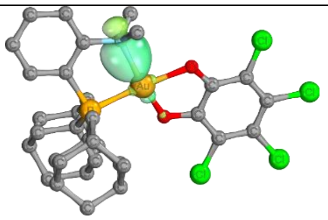	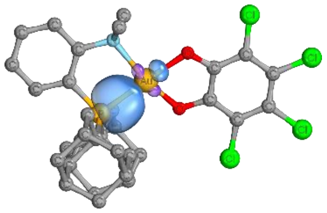
**Pt 29 scan**<br>**D : -147.5°**<br>**NBO: d^{9}**	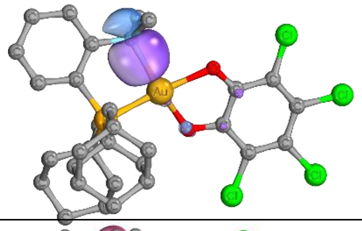	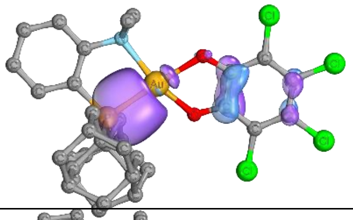
**Pt 31 scan**<br>**D : -145.5°**<br>**NBO: $d^{9.6}$**	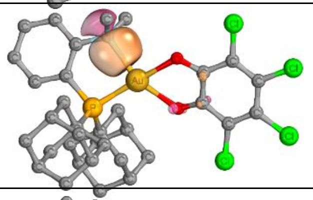	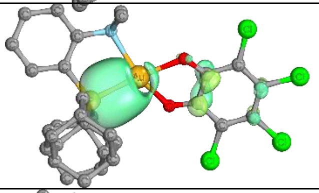
**Pt 35 scan**<br>**D : -141.5°**<br>**NBO: $d^{9.7}$**	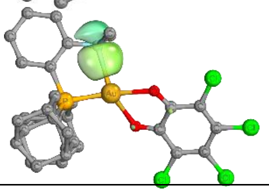	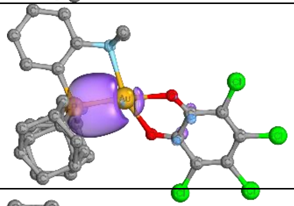
**TS1[&]**<br>**D : -126.9**<br>**NBO: $d^{9.7}$**	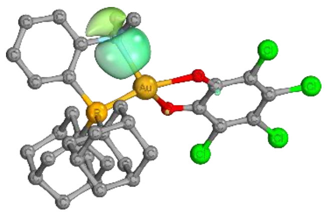	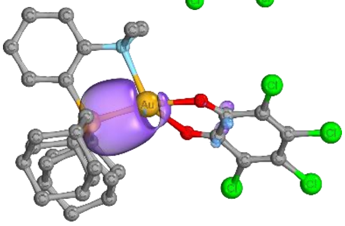

	O→Au (O-Au bond)	O→Au (O-Au bond)
**5a[&]**<br>**D : -175.5°**<br>**NBO: d^8**	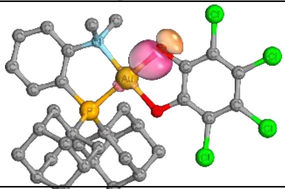	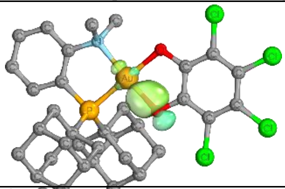
**Pt 14 scan**<br>**D : -162.5°**<br>**NBO: d^8**	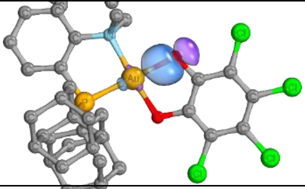	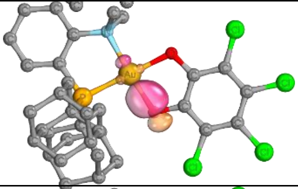
**Pt 21 scan**<br>**D : -155.5°**<br>**NBO: d^8**	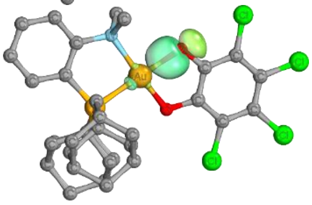	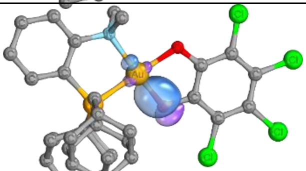
**Pt 27 scan**<br>**D : -149.5°**<br>**NBO: d^8**	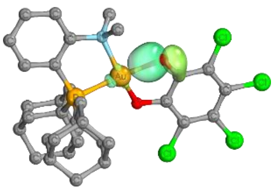	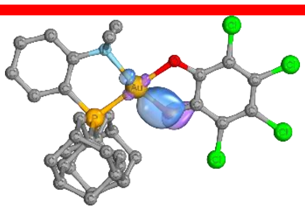
**Pt 29 scan**<br>**D : -147.5°**<br>**NBO: d^9**	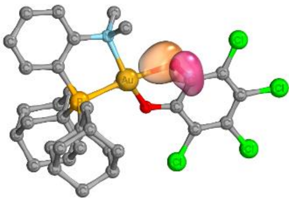	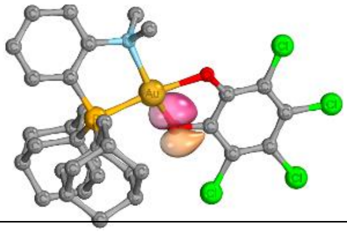
**Pt 31 scan**<br>**D : -145.5°**<br>**NBO: $d^{9.6}$**	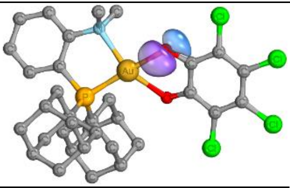	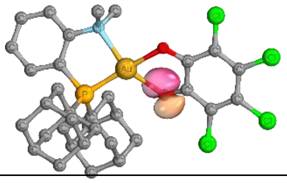
**Pt 35 scan**<br>**D : -141.5°**<br>**NBO: $d^{9.7}$**	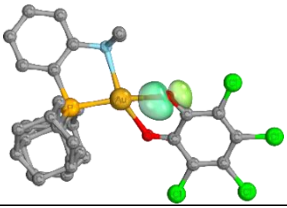	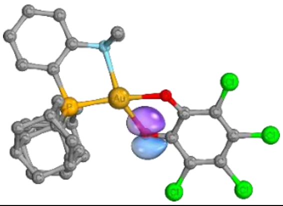
**TS1[&]**<br>**D : -126.9**<br>**NBO: $d^{9.7}$**	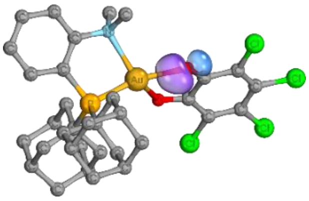	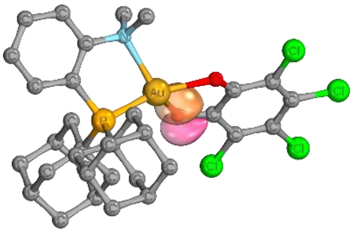

From TS1 to 5a, increase of O→Au interaction.

	n_O *versus* π_{CO}	n_O *versus* π_{CO}
**5a[&]**<br>**D : -175.5°**<br>**NBO: d^8**	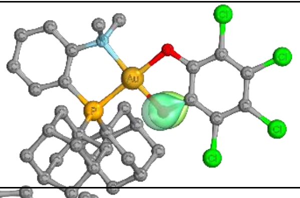	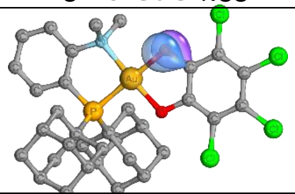
**Pt 14 scan**<br>**D : -162.5°**<br>**NBO: d^8**	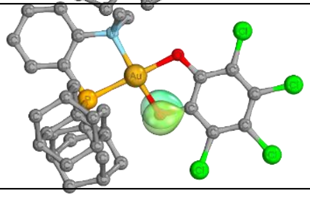	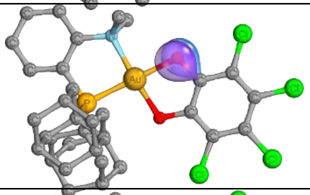
**Pt 21 scan**<br>**D : -155.5°**<br>**NBO: d^8**	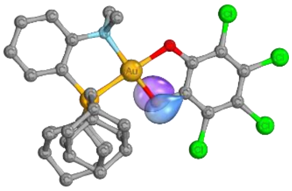	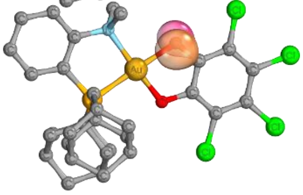
**Pt 27 scan**<br>**D : -149.5°**<br>**NBO: d^8**	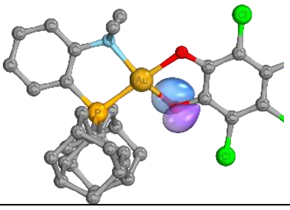	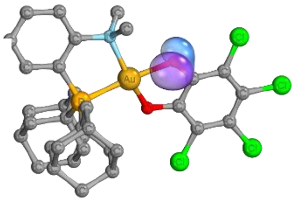
**Pt 29 scan**<br>**D : -147.5°**<br>**NBO: d^9**	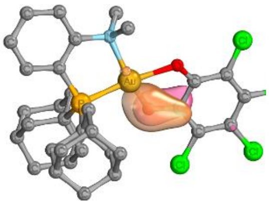	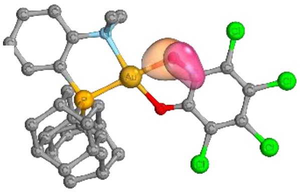
**Pt 31 scan**<br>**D : -145.5°**<br>**NBO: $d^{9.6}$**	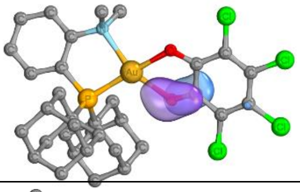	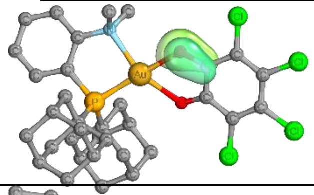
**Pt 35 scan**<br>**D : -141.5°**<br>**NBO: $d^{9.7}$**	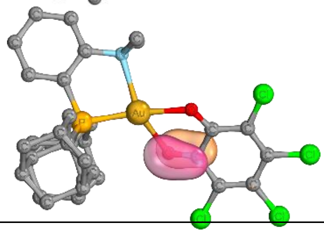	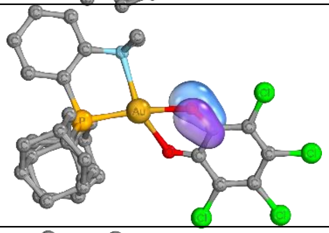
**TS1[&]**<br>**D : -126.9**<br>**NBO: $d^{9.7}$**	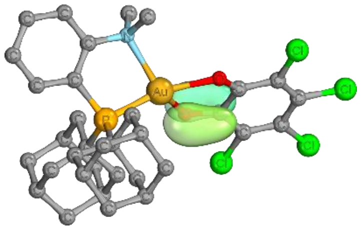	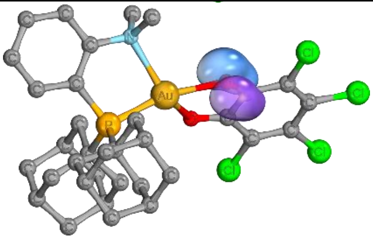

n_O^{π}
(catecholate)

↑

π_{CO}
(o-quinone)

Figure S47. Optimized geometry and frontier orbitals of the Au(III) catecholate complex **2a-H** (right) computed at PCM(DCM)-B3PW91-D3(BJ)/SDD+f(Au),6-31G**(H,B,C,N,O,P). Distances in Å, bond angles in °. & Optimized geometry of Au(I) *o*-quinone **2'a-H** (left), located as a transition state on the PES (reaction coordinate $OAuPC_B$: -147.8°). Relative stability (ΔG in kcal/mol).

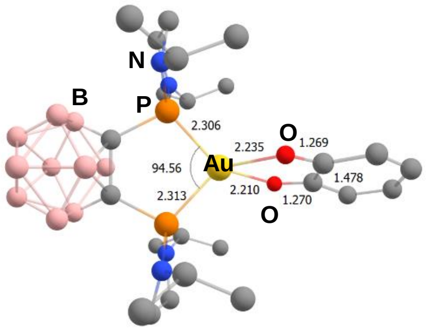

2'a-H (TS)&
(+ 6.4 kcal/mol)

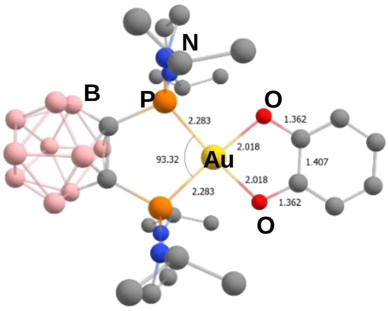

2a-H
(0)

LUMO

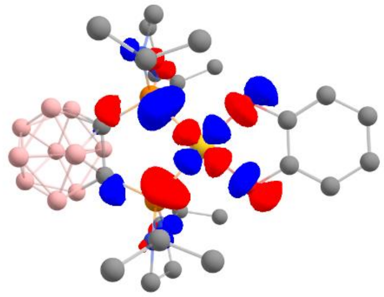

HOMO

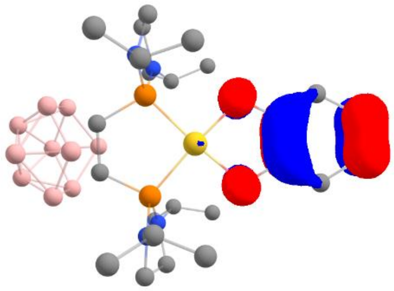

Figure S48. Optimized geometry of the Au(III) catecholate complex **2a** and Au(I) *o*-quinone complex **2’a**, computed at PCM(DCM)-B3PW91-D3(BJ)/SDD+f(Au),6-31G**(H,B,C,N,O,P,Cl). Distances in Å, bond angles in °. [&] The structure of **2’a** has been only found by imposing constraint (reaction coordinate OAuPC$_B$ frozen at -140.9°). Relative stability (ΔG in kcal/mol).

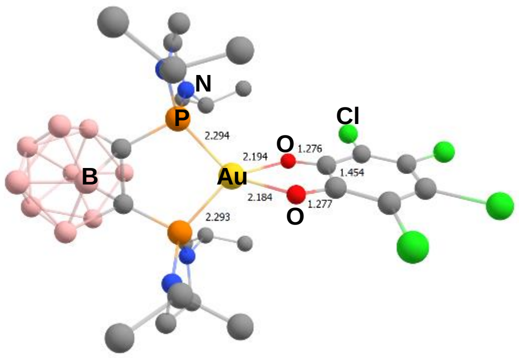

2’a (OAuPC$_B$ frozen at -140.9°)[&]
(+ 14.2 kcal/mol)

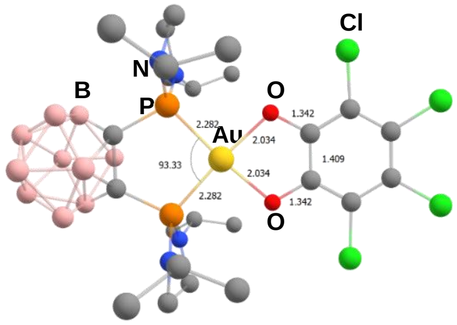

2a
(0)

LUMO

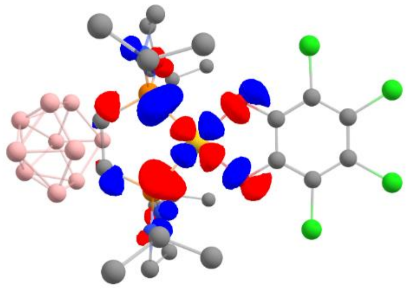

HOMO

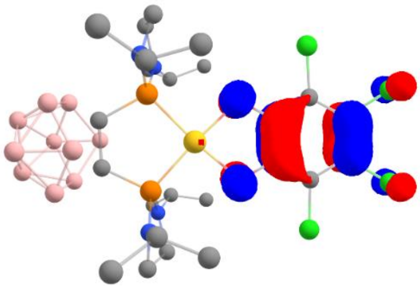

Figure S49. Optimized geometries and frontier orbitals of the Au(I) *o*-benzoquinone (**2'a**, left) and Au(III) catecholate (**2a**, right) valence isomers of the (P^P)Au(O^O)Cl_4^+ complex, computed without dispersion effect at PCM(DCM)-B3PW91/SDD+f(Au),6-31G**(B,C,H,N,O,P,Cl). Distances in Å, bond angles in °. Frontier orbitals (cutoff : 0.05). Hydrogen atoms have been omitted for clarity. Relative stability (ΔG in kcal/mol).

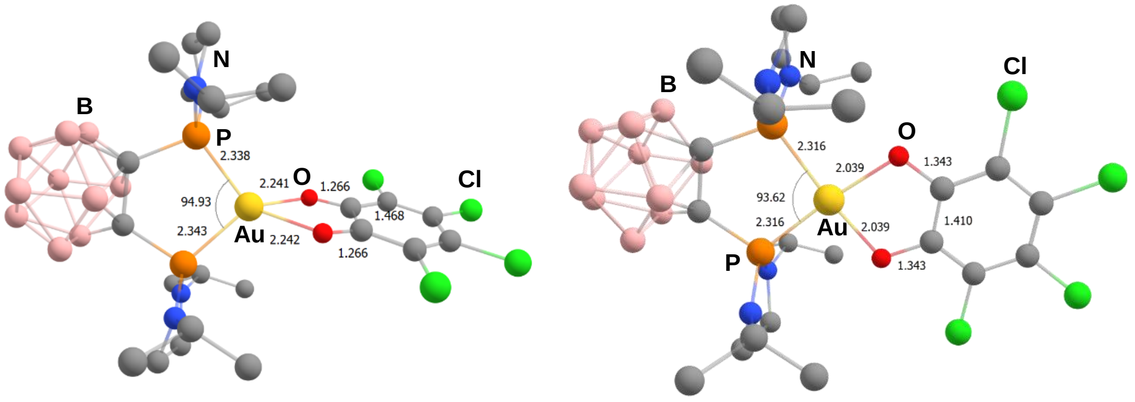

2'a
(+ 6.8 kcal/mol)

2a
(0)

LUMO **LUMO**

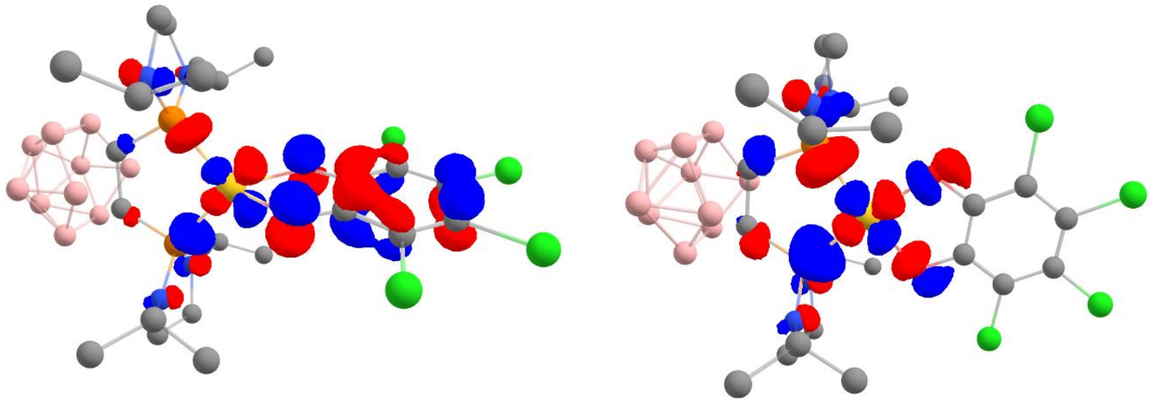

HOMO **HOMO**

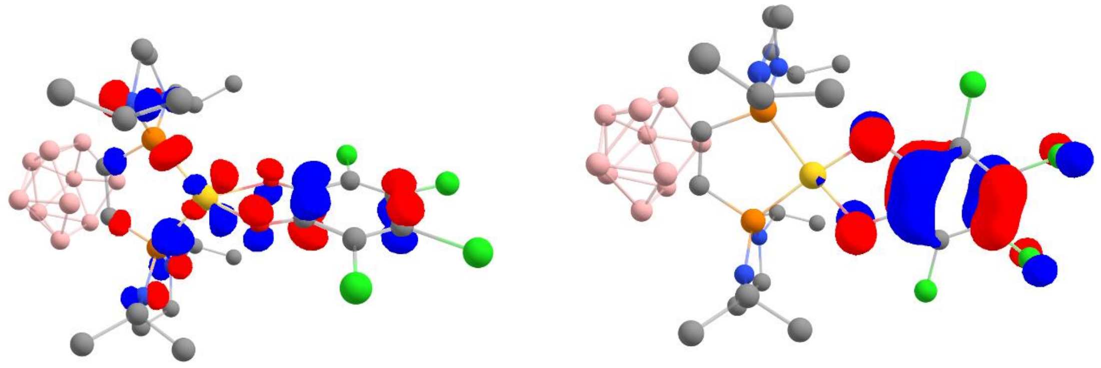

Figure S50. Optimized geometries and frontier orbitals of the Au(I) *o*-benzoquinone (**2'a-H**, left) and Au(III) catecholate (**2a-H**, right) valence isomers of the (P^P)Au(O^O)H_4^+ complex, computed without dispersion at PCM(DCM)-B3PW91/SDD+f(Au),6-31G**(B,C,H,N,O,P). Distances in Å, bond angles in °. Frontier molecular orbitals (cutoff : 0.05). Hydrogen atoms have been omitted for clarity. Relative stability (ΔG in kcal/mol).

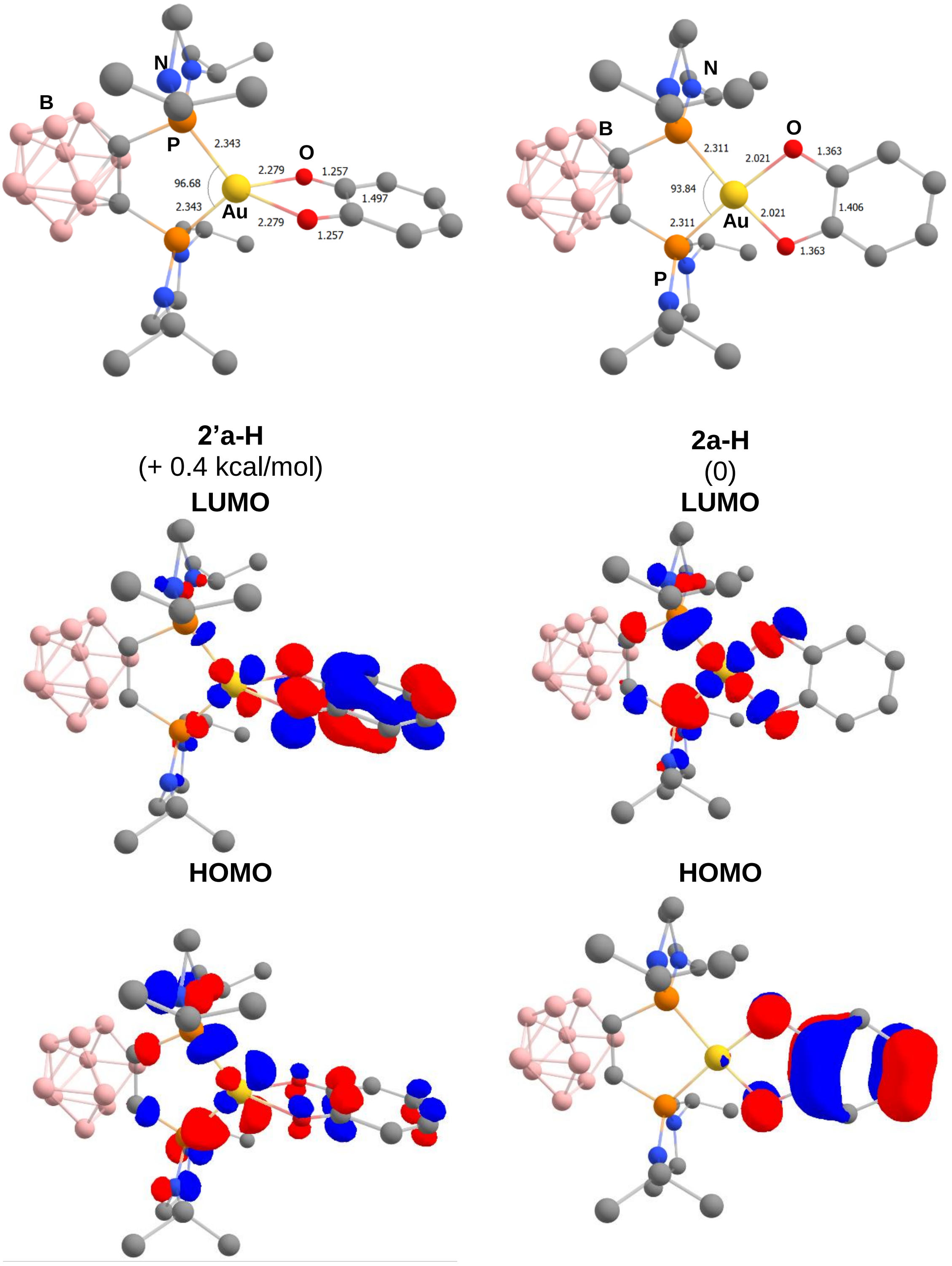

6. Z-matrices and energies in au

(P,N)Au$^+$(O,O)X$_4$ (X: Cl or H)
at B3PW91-D3(BJ) level with dispersion

5a
Sum of electronic & zero-point Energies -3841.601340
Sum of electronic & thermal Free Energies -3841.671203

Au	0.197899000	-1.008970000	0.169122000
Cl	4.846893000	-2.799239000	0.354327000
Cl	6.961583000	-0.487552000	-0.128152000
Cl	5.947642000	2.448959000	-0.663350000
Cl	2.821307000	3.022907000	-0.712055000
P	-1.869774000	-0.025236000	0.106095000
O	2.049883000	-1.863246000	0.228056000
O	1.197049000	0.648033000	-0.247732000
N	-0.750956000	-2.789831000	0.678282000
C	-2.862142000	-1.451011000	0.619080000
C	-2.217837000	-2.673322000	0.830716000
C	-2.946710000	-3.805444000	1.182814000
H	-2.455123000	-4.756176000	1.353013000
C	-4.329147000	-3.720730000	1.315504000
H	-4.891074000	-4.607021000	1.589046000
C	-4.986201000	-2.512768000	1.094062000
H	-6.064375000	-2.448966000	1.189758000
C	-4.256925000	-1.383211000	0.745548000
H	-4.775249000	-0.449927000	0.565598000
C	-0.124222000	-3.222179000	1.970996000
H	-0.401336000	-2.515474000	2.751703000
H	0.956987000	-3.236833000	1.836848000
H	-0.480065000	-4.220949000	2.229520000
C	-0.409461000	-3.778995000	-0.394499000
H	-0.757773000	-4.770181000	-0.099898000
H	0.673470000	-3.788852000	-0.515931000
H	-0.894893000	-3.478215000	-1.321816000
C	-1.946786000	1.312840000	1.392049000
C	-1.216284000	2.565357000	0.867775000
H	-0.194809000	2.310234000	0.568976000
H	-1.733915000	2.964140000	-0.010108000
C	-1.201576000	3.635500000	1.967806000
H	-0.677443000	4.515611000	1.579568000
C	-2.643464000	4.003838000	2.333019000
H	-3.165846000	4.401339000	1.453868000
H	-2.649160000	4.791078000	3.095859000
C	-3.368181000	2.762308000	2.864468000
H	-4.404753000	3.013505000	3.115246000
C	-2.644621000	2.232413000	4.106390000
H	-2.641644000	2.996108000	4.892713000
H	-3.170413000	1.356470000	4.506133000
C	-1.206037000	1.862028000	3.732800000
H	-0.682465000	1.461722000	4.607984000
C	-1.218753000	0.778588000	2.645816000
H	-1.723328000	-0.121316000	3.018483000
H	-0.185937000	0.507112000	2.398228000
C	-3.395368000	1.666408000	1.785551000
H	-3.899857000	0.783581000	2.189961000
H	-3.965563000	2.015093000	0.920240000
C	-0.469934000	3.097290000	3.201908000
H	0.564468000	2.839107000	2.943606000
H	-0.428443000	3.869268000	3.979011000
C	-2.323000000	0.424364000	-1.641752000
C	-2.568362000	-0.889990000	-2.412850000
H	-1.667133000	-1.514540000	-2.387864000
H	-3.381038000	-1.460721000	-1.952850000
C	-2.924784000	-0.567310000	-3.871189000
H	-3.097711000	-1.512905000	-4.396854000
C	-1.769594000	0.194564000	-4.528546000
H	-0.860187000	-0.419128000	-4.524984000
H	-2.011844000	0.409566000	-5.575782000
C	-1.526786000	1.500387000	-3.765654000
H	-0.690639000	2.046102000	-4.216473000
C	-2.791499000	2.364897000	-3.797385000
H	-3.046472000	2.618767000	-4.832818000
H	-2.617861000	3.308328000	-3.265065000
C	-3.948679000	1.598044000	-3.148795000
H	-4.856019000	2.211953000	-3.158029000
C	-3.598346000	1.289526000	-1.684660000
H	-4.437159000	0.765875000	-1.214101000
H	-3.443101000	2.227895000	-1.144430000
C	-4.196418000	0.287777000	-3.903348000
H	-4.475950000	0.501945000	-4.941290000
H	-5.031710000	-0.258525000	-3.447893000
C	-1.154646000	1.179702000	-2.311723000
H	-0.924817000	2.102384000	-1.774565000
H	-0.249168000	0.566341000	-2.289616000
C	2.969055000	-0.908894000	0.024137000
C	4.340239000	-1.172803000	0.048482000
C	5.267347000	-0.140643000	-0.165461000
C	4.819129000	1.164482000	-0.404764000
C	3.441649000	1.432003000	-0.430470000
C	2.527839000	0.402921000	-0.221280000

5'a
Sum of electronic & zero-point Energies -3841.579566
Sum of electronic & thermal Free Energies -3841.654017

Au	0.078846000	-0.724818000	0.484715000
Cl	4.258529000	-3.401216000	-0.266775000
Cl	6.919799000	-1.759813000	-0.704910000
Cl	6.901336000	1.335103000	-0.622722000
Cl	4.217354000	2.904120000	-0.025942000
P	-1.973913000	0.149623000	0.070739000
O	1.964260000	-1.617801000	0.130768000
O	2.011467000	1.033017000	0.570333000
N	-0.831026000	-1.003499000	2.713615000
C	-2.804516000	0.127588000	1.694059000
C	-2.160858000	-0.429844000	2.811559000
C	-2.802310000	-0.433232000	4.052366000
H	-2.307603000	-0.868800000	4.914310000
C	-4.068729000	0.120524000	4.198411000
H	-4.550727000	0.110693000	5.170532000
C	-4.709230000	0.688416000	3.099943000
H	-5.694451000	1.130255000	3.204277000
C	-4.080304000	0.688599000	1.861542000
H	-4.587993000	1.135811000	1.016921000
C	0.135511000	-0.304391000	3.581235000
H	0.120768000	0.760396000	3.347644000
H	1.134086000	-0.701578000	3.389024000
H	-0.100612000	-0.444902000	4.643380000
C	-0.842840000	-2.454927000	2.965077000
H	-1.157141000	-2.680638000	3.992001000
H	0.161699000	-2.851348000	2.808521000
H	-1.530757000	-2.934793000	2.268455000

C	-1.777366000	1.933471000	-0.448012000
C	-0.675847000	2.027431000	-1.528469000
H	0.243709000	1.566577000	-1.153977000
H	-0.971620000	1.482149000	-2.428995000
C	-0.411433000	3.497819000	-1.878086000
H	0.365922000	3.532812000	-2.650283000
C	-1.698174000	4.141775000	-2.404541000
H	-2.033213000	3.628604000	-3.314846000
H	-1.513005000	5.189038000	-2.672135000
C	-2.782281000	4.061899000	-1.324903000
H	-3.711055000	4.510906000	-1.695292000
C	-2.313186000	4.801281000	-0.066260000
H	-2.141483000	5.859271000	-0.298093000
H	-3.090259000	4.760613000	0.707573000
C	-1.020269000	4.160847000	0.451421000
H	-0.685515000	4.679037000	1.357541000
C	-1.291554000	2.693473000	0.805846000
H	-2.045630000	2.638945000	1.597769000
H	-0.374752000	2.226657000	1.186404000
C	-3.061165000	2.588672000	-0.985266000
H	-3.864049000	2.538192000	-0.243658000
H	-3.405004000	2.066849000	-1.884486000
C	0.067479000	4.237397000	-0.624771000
H	0.995053000	3.784809000	-0.254526000
H	0.285121000	5.285163000	-0.865154000
C	-2.960009000	-0.946466000	-1.068414000
C	-2.778478000	-2.387823000	-0.543783000
H	-1.709796000	-2.632496000	-0.491589000
H	-3.186706000	-2.465664000	0.471673000
C	-3.488245000	-3.378409000	-1.475184000
H	-3.341920000	-4.390845000	-1.081592000
C	-2.881009000	-3.271867000	-2.878895000
H	-1.812491000	-3.519862000	-2.847209000
H	-3.362019000	-3.992398000	-3.551095000
C	-3.075605000	-1.848315000	-3.411776000
H	-2.638923000	-1.763091000	-4.413404000
C	-4.570293000	-1.512051000	-3.465088000
H	-5.086158000	-2.199586000	-4.145970000
H	-4.714010000	-0.497627000	-3.858095000
C	-5.168500000	-1.623963000	-2.058477000
H	-6.235091000	-1.373202000	-2.086890000
C	-4.467657000	-0.636375000	-1.111276000
H	-4.897813000	-0.739494000	-0.110504000
H	-4.647506000	0.388406000	-1.449019000
C	-4.983828000	-3.050505000	-1.531180000
H	-5.499835000	-3.763121000	-2.185770000
H	-5.428917000	-3.144571000	-0.532729000
C	-2.369983000	-0.847909000	-2.487288000
H	-2.508420000	0.163844000	-2.882538000
H	-1.291440000	-1.048567000	-2.458010000
C	3.058706000	-1.009358000	0.068468000
C	4.295038000	-1.686860000	-0.185796000
C	5.443421000	-0.959356000	-0.384667000
C	5.433558000	0.498224000	-0.343570000
C	4.282461000	1.192622000	-0.085085000
C	3.054409000	0.482164000	0.212207000

TS1

Sum of electronic & zero-point Energies -3841.579070

Sum of electronic & thermal Free Energies -3841.651476

Au	-0.122782000	-0.650015000	-0.579220000
Cl	-4.556663000	-3.178223000	-0.376035000
Cl	-7.023973000	-1.369519000	0.418196000
Cl	-6.652658000	1.666157000	0.942264000
Cl	-3.795856000	3.002056000	0.618700000
P	1.958852000	0.090991000	-0.037700000
O	-2.050504000	-1.600798000	-0.363101000
O	-1.847939000	1.045997000	-0.468597000
N	0.967382000	-1.562428000	-2.469538000
C	2.956690000	-0.427791000	-1.473831000
C	2.374996000	-1.190965000	-2.499146000
C	3.149438000	-1.605817000	-3.584050000
H	2.702254000	-2.200872000	-4.373094000
C	4.493143000	-1.260383000	-3.668344000
H	5.079313000	-1.589641000	-4.519941000
C	5.077284000	-0.493190000	-2.664211000
H	6.123723000	-0.213634000	-2.722595000
C	4.313974000	-0.085288000	-1.577667000
H	4.783076000	0.506347000	-0.802762000
C	0.238374000	-1.005343000	-3.629738000
H	0.383729000	0.074742000	-3.657877000
H	-0.824128000	-1.228120000	-3.521604000
H	0.595170000	-1.443070000	-4.569506000
C	0.791797000	-3.025849000	-2.372571000
H	1.184426000	-3.530511000	-3.263015000
H	-0.271847000	-3.248486000	-2.277453000
H	1.317961000	-3.391653000	-1.491057000
C	1.954713000	1.954957000	0.034738000
C	0.840875000	2.432961000	0.992744000
H	-0.115532000	1.988578000	0.705677000
H	1.057338000	2.119183000	2.017219000
C	0.735074000	3.963293000	0.943825000
H	-0.060041000	4.272580000	1.631961000
C	2.068227000	4.583898000	1.373370000
H	2.309147000	4.288216000	2.402226000
H	1.997828000	5.678189000	1.358119000
C	3.171952000	4.118210000	0.418672000
H	4.134249000	4.545050000	0.723826000
C	2.837893000	4.559376000	-1.010893000
H	2.783745000	5.653462000	-1.060139000
H	3.631288000	4.242378000	-1.699560000
C	1.497085000	3.948722000	-1.435138000
H	1.257291000	4.253222000	-2.460433000
C	1.604716000	2.419519000	-1.396950000
H	2.372063000	2.082624000	-2.102514000
H	0.650860000	1.972201000	-1.705857000
C	3.288217000	2.585820000	0.472002000
H	4.103501000	2.268793000	-0.184297000
H	3.536503000	2.274919000	1.492024000
C	0.389020000	4.406585000	-0.481017000
H	-0.572312000	3.974624000	-0.784288000
H	0.285983000	5.497654000	-0.521324000
C	2.627430000	-0.832213000	1.437750000
C	2.355635000	-2.328193000	1.164441000
H	1.284154000	-2.486631000	0.986989000
H	2.893436000	-2.643602000	0.261938000
C	2.804394000	-3.170420000	2.364992000
H	2.599385000	-4.224254000	2.144403000
C	2.017442000	-2.734394000	3.605895000
H	0.943278000	-2.890381000	3.444721000
H	2.308755000	-3.345218000	4.468607000
C	2.298312000	-1.255732000	3.893164000
H	1.734646000	-0.934387000	4.776373000
C	3.798729000	-1.047396000	4.128787000
H	4.127307000	-1.629051000	4.998289000
H	4.001912000	0.008203000	4.348571000

C	4.575989000	-1.487026000	2.883396000
H	5.649194000	-1.328427000	3.039138000
C	4.138954000	-0.649710000	1.669904000
H	4.695959000	-0.984583000	0.789938000
H	4.383746000	0.402490000	1.842065000
C	4.304502000	-2.968267000	2.602121000
H	4.635539000	-3.578739000	3.450689000
H	4.873523000	-3.296568000	1.723328000
C	1.857471000	-0.399276000	2.698700000
H	2.065099000	0.652149000	2.920308000
H	0.777036000	-0.497092000	2.533163000
C	-3.099193000	-0.912626000	-0.242279000
C	-4.402014000	-1.485099000	-0.128416000
C	-5.465018000	-0.685453000	0.231913000
C	-5.290130000	0.732381000	0.481737000
C	-4.059153000	1.328424000	0.345398000
C	-2.945173000	0.556786000	-0.134490000

5a-H

Sum of electronic & zero-point Energies -2003.389914

Sum of electronic & thermal Free Energies -2003.451333

Au	1.282585000	-0.309739000	0.577915000
P	-0.940002000	0.071422000	0.160328000
O	1.894097000	0.087054000	-1.255328000
O	3.241201000	-0.617343000	0.976289000
N	0.753770000	-0.705946000	2.563847000
C	-1.583599000	-0.199702000	1.834374000
C	-0.691010000	-0.572852000	2.843822000
C	-1.149659000	-0.831523000	4.132823000
H	-0.463764000	-1.119821000	4.920778000
C	-2.506918000	-0.725166000	4.418001000
H	-2.856196000	-0.930066000	5.424110000
C	-3.409228000	-0.364393000	3.420217000
H	-4.468430000	-0.287921000	3.639546000
C	-2.950054000	-0.104408000	2.135554000
H	-3.658842000	0.166699000	1.363706000
C	1.533909000	0.263347000	3.399777000
H	1.164604000	1.270178000	3.208295000
H	2.583361000	0.184209000	3.117522000
H	1.409391000	0.017721000	4.455929000
C	1.219789000	-2.099896000	2.850985000
H	1.118556000	-2.310350000	3.917314000
H	2.266746000	-2.172077000	2.557205000
H	0.614575000	-2.800048000	2.276715000
C	-1.194596000	1.848856000	-0.319990000
C	-0.775244000	2.040206000	-1.790436000
H	0.248518000	1.680472000	-1.938982000
H	-1.433684000	1.462437000	-2.446894000
C	-0.887115000	3.526357000	-2.157471000
H	-0.582243000	3.644589000	-3.203161000
C	-2.341168000	3.978485000	-1.983640000
H	-2.998408000	3.394002000	-2.639607000
H	-2.445750000	5.030437000	-2.274101000
C	-2.759614000	3.800502000	-0.519953000
H	-3.803035000	4.108724000	-0.389801000
C	-1.851371000	4.639185000	0.384410000
H	-1.940674000	5.700730000	0.125713000
H	-2.158576000	4.532475000	1.432213000
C	-0.401143000	4.178987000	0.208600000
H	0.258046000	4.757343000	0.865524000
C	-0.277034000	2.697410000	0.588492000
H	-0.552410000	2.551157000	1.640704000
H	0.767356000	2.386166000	0.467865000
C	-2.648106000	2.320511000	-0.117342000
H	-2.931885000	2.217561000	0.934502000
H	-3.342613000	1.723371000	-0.714326000
C	0.028998000	4.356499000	-1.251960000
H	1.071933000	4.041137000	-1.379210000
H	-0.027731000	5.414908000	-1.531834000
C	-1.645871000	-1.236648000	-0.961626000
C	-1.634360000	-2.565242000	-0.174735000
H	-0.610002000	-2.808619000	0.133471000
H	-2.243778000	-2.481792000	0.730664000
C	-2.179420000	-3.692194000	-1.062969000
H	-2.167220000	-4.621339000	-0.482267000
C	-1.296226000	-3.839459000	-2.305903000
H	-0.270334000	-4.095344000	-2.012944000
H	-1.668288000	-4.657015000	-2.934501000
C	-1.307329000	-2.524635000	-3.091662000
H	-0.664725000	-2.612960000	-3.974734000
C	-2.739484000	-2.188533000	-3.520752000
H	-3.132652000	-2.979494000	-4.170077000
H	-2.752106000	-1.256750000	-4.099833000
C	-3.622832000	-2.047838000	-2.276658000
H	-4.647587000	-1.796568000	-2.572151000
C	-3.086594000	-0.907290000	-1.397077000
H	-3.738545000	-0.784841000	-0.526125000
H	-3.107028000	0.027813000	-1.964938000
C	-3.615490000	-3.357440000	-1.481012000
H	-4.024694000	-4.168708000	-2.094259000
H	-4.255765000	-3.265650000	-0.594772000
C	-0.753263000	-1.396026000	-2.211241000
H	-0.718160000	-0.466472000	-2.784011000
H	0.274190000	-1.621928000	-1.913739000
C	3.262111000	-0.008886000	-1.314732000
C	3.939102000	0.252029000	-2.500681000
C	5.332046000	0.140455000	-2.527841000
C	6.030058000	-0.227626000	-1.378697000
C	5.347648000	-0.488914000	-0.187845000
C	3.958844000	-0.378409000	-0.153348000
H	5.877419000	-0.775692000	0.715640000
H	7.112233000	-0.312720000	-1.402476000
H	5.865767000	0.343970000	-3.451007000
H	3.374807000	0.537551000	-3.383371000

5’a-H

Sum of electronic & zero-point Energies -2003.381817

Sum of electronic & thermal Free Energies -2003.449519

Au	1.060338000	-0.800555000	-0.094394000
P	-0.972228000	0.149907000	0.185775000
O	2.842991000	-1.734914000	-0.777011000
O	3.239486000	0.837718000	0.065320000
N	0.662405000	-1.334868000	2.397234000
C	-1.397180000	-0.013301000	1.955290000
C	-0.560157000	-0.714331000	2.843859000
C	-0.914443000	-0.810748000	4.193025000
H	-0.270235000	-1.359103000	4.873121000
C	-2.073018000	-0.214730000	4.675787000
H	-2.327033000	-0.300604000	5.727535000
C	-2.899057000	0.493217000	3.806144000
H	-3.802971000	0.970814000	4.169548000
C	-2.561106000	0.587756000	2.462280000
H	-3.212315000	1.143394000	1.800351000
C	1.845872000	-0.795872000	3.075088000
H	1.860585000	0.289180000	2.961895000
H	2.740602000	-1.210415000	2.604568000

H	1.868791000	-1.045750000	4.145573000
C	0.601542000	-2.797460000	2.467074000
H	0.524205000	-3.162354000	3.501600000
H	1.507158000	-3.210414000	2.016590000
H	-0.263158000	-3.150472000	1.902893000
C	-0.831674000	1.978827000	-0.187365000
C	-0.003144000	2.155652000	-1.480483000
H	0.952280000	1.629471000	-1.374742000
H	-0.523209000	1.710628000	-2.333754000
C	0.248767000	3.644895000	-1.745781000
H	0.829497000	3.737611000	-2.671147000
C	-1.089990000	4.374858000	-1.896617000
H	-1.650004000	3.965309000	-2.746872000
H	-0.918504000	5.438374000	-2.102729000
C	-1.900365000	4.215109000	-0.606431000
H	-2.864951000	4.726146000	-0.706955000
C	-1.118569000	4.809142000	0.571101000
H	-0.950245000	5.880047000	0.404497000
H	-1.699275000	4.712074000	1.497190000
C	0.223967000	4.082237000	0.713375000
H	0.782947000	4.496986000	1.560401000
C	-0.035005000	2.594880000	0.983543000
H	-0.592400000	2.483271000	1.919165000
H	0.916493000	2.060664000	1.100809000
C	-2.167366000	2.723295000	-0.349581000
H	-2.782193000	2.621435000	0.549543000
H	-2.734067000	2.306343000	-1.188682000
C	1.037822000	4.240001000	-0.575096000
H	2.002948000	3.728317000	-0.474710000
H	1.246205000	5.300835000	-0.760627000
C	-2.284770000	-0.776884000	-0.762300000
C	-2.068024000	-2.271235000	-0.436861000
H	-1.033239000	-2.554128000	-0.668820000
H	-2.222563000	-2.439755000	0.636297000
C	-3.039568000	-3.134344000	-1.250863000
H	-2.861043000	-4.187447000	-1.004078000
C	-2.791798000	-2.900663000	-2.745817000
H	-1.764573000	-3.185461000	-3.006530000
H	-3.464573000	-3.530584000	-3.340301000
C	-3.027725000	-1.423090000	-3.076985000
H	-2.847194000	-1.247851000	-4.143889000
C	-4.467370000	-1.033892000	-2.722123000
H	-5.174329000	-1.628189000	-3.313572000
H	-4.643948000	0.020669000	-2.969507000
C	-4.705976000	-1.273239000	-1.227214000
H	-5.730504000	-0.985897000	-0.963752000
C	-3.736005000	-0.415039000	-0.398977000
H	-3.911898000	-0.606931000	0.663706000
H	-3.933791000	0.644946000	-0.583606000
C	-4.481410000	-2.753162000	-0.899882000
H	-5.183652000	-3.375074000	-1.468150000
H	-4.672040000	-2.937144000	0.164959000
C	-2.057821000	-0.552107000	-2.268669000
H	-2.224549000	0.500380000	-2.521359000
H	-1.018895000	-0.793713000	-2.527618000
C	3.989007000	-1.241692000	-0.813030000
C	5.126772000	-2.004788000	-1.243448000
C	6.325951000	-1.383217000	-1.376138000
C	6.510311000	0.028701000	-1.085420000
C	5.495657000	0.811281000	-0.652586000
C	4.183759000	0.228187000	-0.428598000
H	4.984756000	-3.053480000	-1.479510000
H	7.187221000	-1.946086000	-1.721379000
H	7.501559000	0.448754000	-1.221485000
H	5.624878000	1.862619000	-0.420893000

1,2-benzoquinone

Sum of electronic & zero-point Energies -381.240343

Sum of electronic & thermal Free Energies -381.271235

O	1.729697000	1.371983000	-0.000308000
O	1.729690000	-1.371989000	0.000217000
C	0.664258000	0.778034000	-0.000050000
C	-0.634968000	1.453482000	-0.000133000
C	-1.773514000	0.729797000	-0.000056000
C	-1.773517000	-0.729791000	0.000067000
C	-0.634975000	-1.453480000	0.000146000
C	0.664253000	-0.778036000	0.000161000
H	-0.635789000	-2.538630000	0.000221000
H	-2.736376000	-1.232023000	0.000079000
H	-2.736371000	1.232033000	-0.000112000
H	-0.635778000	2.538632000	-0.000265000

3,4,5,6-tetrachloro-1,2-benzoquinone

Sum of electronic & zero-point Energies -2219.442049

Sum of electronic & thermal Free Energies -2219.479624

O	-1.358961000	2.779617000	0.001543000
O	1.358983000	2.779597000	-0.001435000
C	-0.773057000	1.719510000	0.000541000
C	-1.454525000	0.414744000	-0.000005000
C	-0.738777000	-0.740589000	-0.000120000
C	0.738761000	-0.740591000	0.000138000
C	1.454530000	0.414725000	-0.000022000
C	0.773072000	1.719513000	-0.000503000
Cl	3.166714000	0.453395000	0.000045000
Cl	1.540271000	-2.253336000	0.000512000
Cl	-1.540287000	-2.253327000	-0.000465000
Cl	-3.166710000	0.453409000	-0.000153000

(P,P)Au$^+$(O,O)Cl$_4$
at B3PW91-D3(BJ) level with dispersion

2a

Sum of electronic & zero-point Energies -4218.373956

Sum of electronic & thermal Free Energies -4218.456520

Au	-0.068974000	0.000036000	-0.000090000
P	1.496847000	1.659306000	-0.029529000
P	1.496728000	-1.659321000	0.029697000
N	1.451166000	2.733137000	-1.274335000
N	1.389412000	2.726771000	1.197539000
B	3.573054000	0.071842000	1.439875000
H	2.786548000	0.124567000	2.318040000
B	4.453905000	1.481165000	0.810917000
H	4.277741000	2.519302000	1.346945000
B	4.457563000	1.392300000	-0.961359000
H	4.283725000	2.373875000	-1.592432000
B	5.883425000	0.885182000	-0.046061000
H	6.876775000	1.531848000	-0.078581000
B	5.883372000	-0.885550000	0.044871000
H	6.876683000	-1.532291000	0.077063000
B	4.453519000	-1.481432000	-0.811632000
H	4.277083000	-2.519568000	-1.347576000
B	4.457780000	-1.392568000	0.960644000
H	4.284106000	-2.374143000	1.591751000
B	3.572584000	-0.072043000	-1.440291000
H	2.785800000	-0.124673000	-2.318215000
B	5.332762000	0.074741000	1.447796000
H	5.914170000	0.129490000	2.478865000

B	5.332279000	-0.075072000	-1.448801000
H	5.913341000	-0.129873000	-2.480064000
C	3.151645000	0.812832000	-0.044236000
C	3.151598000	-0.812996000	0.043965000
C	1.096711000	4.070510000	-0.757670000
H	0.013919000	4.221781000	-0.806970000
H	1.598327000	4.833964000	-1.354987000
C	1.579954000	4.096956000	0.690268000
H	2.635569000	4.379829000	0.755611000
H	0.987790000	4.793108000	1.284682000
C	1.077713000	2.343211000	-2.655487000
H	1.211756000	1.256993000	-2.705386000
C	2.023688000	2.989430000	-3.656498000
H	1.942803000	4.079989000	-3.624715000
H	1.763480000	2.666289000	-4.667858000
H	3.060299000	2.710981000	-3.457518000
C	-0.387776000	2.648367000	-2.947901000
H	-1.039691000	2.233616000	-2.174399000
H	-0.659607000	2.208393000	-3.910804000
H	-0.564947000	3.725528000	-3.016260000
C	0.960342000	2.433363000	2.579587000
H	0.932048000	1.340529000	2.654258000
C	1.980684000	2.960576000	3.578072000
H	2.968581000	2.529687000	3.400295000
H	1.667813000	2.707030000	4.594026000
H	2.062536000	4.050066000	3.516618000
C	-0.450934000	2.955121000	2.823369000
H	-0.485117000	4.048178000	2.790002000
H	-0.788218000	2.646208000	3.816610000
H	-1.138411000	2.558303000	2.072657000
N	1.451128000	-2.732909000	1.274754000
C	1.096212000	-4.070275000	0.758376000
H	0.013378000	-4.221215000	0.807770000
H	1.597642000	-4.833769000	1.355799000
C	1.579341000	-4.097135000	-0.689586000
H	2.634907000	-4.380186000	-0.754951000
H	0.987003000	-4.793329000	-1.283775000
C	0.960035000	-2.433781000	-2.579240000
H	0.932023000	-1.340950000	-2.654094000
C	1.980289000	-2.961447000	-3.577576000
H	2.968289000	-2.530770000	-3.399858000
H	1.667517000	-2.708033000	-4.593595000
H	2.061872000	-4.050943000	-3.515890000
C	-0.451382000	-2.955193000	-2.822982000
H	-0.485863000	-4.048238000	-2.789543000
H	-0.788580000	-2.646264000	-3.816247000
H	-1.138755000	-2.558120000	-2.072309000
C	1.077933000	-2.342709000	2.655934000
H	1.212509000	-1.256559000	2.705835000
C	2.023666000	-2.989350000	3.656892000
H	1.942435000	-4.079881000	3.624989000
H	1.763565000	-2.666213000	4.668283000
H	3.060370000	-2.711223000	3.457950000
C	-0.387695000	-2.647108000	2.948426000
H	-1.039405000	-2.231958000	2.174965000
H	-0.659236000	-2.206991000	3.911344000
H	-0.565462000	-3.724169000	3.016784000
N	1.388941000	-2.727026000	-1.197117000
O	-1.588440000	-1.351832000	-0.007770000
O	-1.588448000	1.351900000	0.007501000
C	-2.764557000	-0.704674000	-0.004568000
C	-3.976362000	-1.397743000	-0.010391000
C	-5.193614000	-0.700514000	-0.005622000
C	-5.193621000	0.700544000	0.005517000
C	-3.976376000	1.397787000	0.010234000
C	-2.764563000	0.704733000	0.004364000
Cl	-3.919028000	3.128165000	0.025178000
Cl	-6.686059000	1.575338000	0.013565000
Cl	-6.686043000	-1.575325000	-0.013604000
Cl	-3.918989000	-3.128120000	-0.025346000

2’a (frequency : -5 cm^{-1}, structure found only by imposing constraint C_OOAuP: 126.8°)
Sum of electronic & zero-point Energies -4218.352658
Sum of electronic & thermal Free Energies -4218.433968

Au	0.038572000	0.005207000	-0.015812000
P	-1.522492000	1.516885000	0.714615000
P	-1.523841000	-1.521914000	-0.714993000
N	-1.571235000	1.939613000	2.318085000
N	-1.488377000	3.039821000	0.107297000
B	-3.560355000	0.677713000	-1.338572000
H	-2.749011000	1.077032000	-2.098815000
B	-4.464546000	1.719284000	-0.218478000
H	-4.279497000	2.884499000	-0.283497000
B	-4.520437000	0.916683000	1.360734000
H	-4.369119000	1.549518000	2.346063000
B	-5.920182000	0.847590000	0.280327000
H	-6.913749000	1.439065000	0.546830000
B	-5.919771000	-0.730520000	-0.525333000
H	-6.913289000	-1.294168000	-0.846630000
B	-4.517620000	-1.641544000	0.052474000
H	-4.367648000	-2.811229000	0.129778000
B	-4.465736000	-0.838516000	-1.527562000
H	-4.279772000	-1.476708000	-2.503024000
B	-3.650058000	-0.623513000	1.220765000
H	-2.894263000	-1.043009000	2.024769000
B	-5.322540000	0.709441000	-1.397265000
H	-5.872719000	1.188686000	-2.331983000
B	-5.412271000	-0.607357000	1.182827000
H	-6.026070000	-1.070753000	2.085335000
C	-3.186078000	0.748483000	0.326883000
C	-3.185646000	-0.708089000	-0.421534000
C	-1.342576000	3.386548000	2.465504000
H	-0.279784000	3.594040000	2.633352000
H	-1.917829000	3.764590000	3.313413000
C	-1.812960000	4.016945000	1.155413000
H	-2.891088000	4.210702000	1.173227000
H	-1.290949000	4.955029000	0.963930000
C	-1.146311000	1.001699000	3.378347000
H	-1.217135000	0.002465000	2.934300000
C	-2.107960000	1.070238000	4.556498000
H	-2.086102000	2.057701000	5.027360000
H	-1.820245000	0.334795000	5.312495000
H	-3.131091000	0.860905000	4.237324000
C	0.305947000	1.224219000	3.791730000
H	0.976352000	1.194025000	2.927773000
H	0.610355000	0.433957000	4.482573000
H	0.435732000	2.181106000	4.305254000
C	-0.970336000	3.414042000	-1.219702000
H	-0.852443000	2.470603000	-1.764531000
C	-1.975812000	4.281769000	-1.964077000
H	-2.934704000	3.769683000	-2.070910000
H	-1.594564000	4.518939000	-2.960705000
H	-2.144986000	5.226568000	-1.437969000
C	0.404185000	4.062258000	-1.098376000
H	0.353038000	5.009365000	-0.552731000

H	0.801722000	4.274726000	-2.094433000
H	1.100585000	3.391853000	-0.590111000
N	-1.535749000	-2.033749000	-2.294405000
C	-1.305979000	-3.486962000	-2.353358000
H	-0.239131000	-3.704586000	-2.476238000
H	-1.856109000	-3.910843000	-3.196144000
C	-1.816472000	-4.043954000	-1.025582000
H	-2.893673000	-4.239161000	-1.064900000
H	-1.300840000	-4.969534000	-0.767255000
C	-1.076229000	-3.311548000	1.345695000
H	-0.949799000	-2.337166000	1.831149000
C	-2.142768000	-4.099673000	2.093923000
H	-3.091684000	-3.559050000	2.115501000
H	-1.820269000	-4.278682000	3.122907000
H	-2.311602000	-5.073119000	1.622636000
C	0.280818000	-4.006082000	1.334829000
H	0.225177000	-4.986057000	0.851443000
H	0.621878000	-4.163104000	2.361712000
H	1.022580000	-3.391955000	0.819797000
C	-1.043445000	-1.156438000	-3.377867000
H	-1.146825000	-0.135338000	-2.995332000
C	-1.923512000	-1.297109000	-4.611679000
H	-1.869252000	-2.309554000	-5.023232000
H	-1.584942000	-0.604770000	-5.387022000
H	-2.965775000	-1.073033000	-4.375156000
C	0.433926000	-1.394422000	-3.680008000
H	1.040983000	-1.333203000	-2.771755000
H	0.789910000	-0.630927000	-4.376243000
H	0.597269000	-2.370203000	-4.146322000
N	-1.519124000	-3.010031000	-0.025587000
O	1.788021000	-0.905266000	0.944674000
O	1.790739000	0.904722000	-0.959292000
C	2.919271000	-0.543808000	0.477415000
C	4.155306000	-1.110439000	0.886255000
C	5.346582000	-0.559747000	0.444803000
C	5.347823000	0.563130000	-0.443825000
C	4.157699000	1.112331000	-0.890787000
C	2.921055000	0.545004000	-0.486136000
Cl	4.110220000	2.444990000	-1.981581000
Cl	6.848720000	1.220441000	-0.969229000
Cl	6.845908000	-1.215513000	0.976672000
Cl	4.103853000	-2.444125000	1.975567000

2a-H

Sum of electronic & zero-point Energies -2380.161063

Sum of electronic & thermal Free Energies -2380.233456

Au	-0.939349000	0.000043000	0.000055000
P	0.627523000	1.660285000	-0.017777000
P	0.627362000	-1.660351000	0.017828000
N	0.611894000	2.752249000	-1.251458000
N	0.537530000	2.722170000	1.218030000
B	2.707702000	0.059399000	1.438739000
H	1.922353000	0.104591000	2.318609000
B	3.589383000	1.473450000	0.823298000
H	3.415593000	2.506732000	1.369763000
B	3.593193000	1.399562000	-0.948709000
H	3.421756000	2.386419000	-1.572355000
B	5.019429000	0.885463000	-0.038078000
H	6.013219000	1.532278000	-0.065138000
B	5.019350000	-0.885952000	0.037835000
H	6.013082000	-1.532858000	0.064825000
B	3.589189000	-1.473807000	-0.823439000
H	3.415264000	-2.507072000	-1.369894000
B	3.593131000	-1.399920000	0.948566000
H	3.421650000	-2.386763000	1.572218000
B	2.707596000	-0.059675000	-1.438817000
H	1.922181000	-0.104797000	-2.318629000
B	4.468194000	0.062375000	1.448295000
H	5.049876000	0.108334000	2.480006000
B	4.468087000	-0.062814000	-1.448499000
H	5.049691000	-0.108827000	-2.480251000
C	2.286566000	0.813919000	-0.037210000
C	2.286492000	-0.814155000	0.037162000
C	0.296765000	4.093700000	-0.724481000
H	-0.780069000	4.282299000	-0.780675000
H	0.827047000	4.848162000	-1.308812000
C	0.767918000	4.090931000	0.728654000
H	1.829796000	4.347093000	0.805411000
H	0.189829000	4.796706000	1.326081000
C	0.224112000	2.379826000	-2.631888000
H	0.348478000	1.292876000	-2.691201000
C	1.168826000	3.024680000	-3.635300000
H	1.097385000	4.115768000	-3.596149000
H	0.900263000	2.710476000	-4.647367000
H	2.204363000	2.736571000	-3.444131000
C	-1.240972000	2.697903000	-2.911855000
H	-1.887956000	2.277047000	-2.137259000
H	-1.523300000	2.270953000	-3.877747000
H	-1.411071000	3.777178000	-2.966624000
C	0.093342000	2.421843000	2.592092000
H	0.053580000	1.328587000	2.657222000
C	1.109716000	2.929887000	3.604707000
H	2.095404000	2.492672000	3.429571000
H	0.787157000	2.668531000	4.615714000
H	1.201368000	4.019269000	3.555338000
C	-1.315033000	2.955127000	2.827556000
H	-1.339978000	4.048428000	2.786988000
H	-1.660773000	2.654361000	3.820513000
H	-1.998756000	2.556879000	2.073797000
N	0.611635000	-2.752307000	1.251516000
C	0.296282000	-4.093706000	0.724540000
H	-0.780581000	-4.282131000	0.780749000
H	0.826452000	-4.848251000	1.308869000
C	0.767417000	-4.091015000	-0.728601000
H	1.829261000	-4.347314000	-0.805377000
H	0.189224000	-4.796716000	-1.326012000
C	0.092957000	-2.421848000	-2.592013000
H	0.053387000	-1.328586000	-2.657172000
C	1.109156000	-2.930103000	-3.604695000
H	2.094938000	-2.493064000	-3.429646000
H	0.786566000	-2.668714000	-4.615684000
H	1.200612000	-4.019500000	-3.555303000
C	-1.315537000	-2.954872000	-2.827356000
H	-1.340685000	-4.048168000	-2.786776000
H	-1.661303000	-2.654052000	-3.820288000
H	-1.999125000	-2.556489000	-2.073546000
C	0.223915000	-2.379817000	2.631950000
H	0.348920000	-1.292949000	2.691437000
C	1.168087000	-3.025371000	3.635423000
H	1.096031000	-4.116412000	3.596086000
H	0.899531000	-2.711169000	4.647493000
H	2.203819000	-2.737822000	3.444478000
C	-1.241405000	-2.697022000	2.911652000
H	-1.888001000	-2.275604000	2.137033000
H	-1.523598000	-2.270061000	3.877578000
H	-1.412198000	-3.776198000	2.966207000

N	0.537195000	-2.722226000	-1.217974000
O	-2.435097000	-1.354436000	-0.014339000
O	-2.434990000	1.354643000	0.014312000
C	-3.631141000	-0.703316000	-0.005464000
C	-4.836243000	-1.402848000	-0.008834000
C	-6.041032000	-0.697174000	-0.004412000
C	-6.040977000	0.697670000	0.004159000
C	-4.836132000	1.403248000	0.008657000
C	-3.631085000	0.703620000	0.005369000
H	-4.815692000	2.488894000	0.016302000
H	-6.979510000	1.243510000	0.007792000
H	-6.979608000	-1.242940000	-0.008106000
H	-4.815889000	-2.488495000	-0.016481000

2'a-H (freq: -20.9 cm^{-1}, TS, reaction coordinate: $OAuPC_B$)
Sum of electronic & zero-point Energies -2380.147633
Sum of electronic & thermal Free Energies -2380.223205

Au	0.925605000	-0.111957000	0.066977000
P	-0.481395000	1.718200000	-0.069956000
P	-0.785438000	-1.657970000	0.081698000
N	-0.563113000	2.875490000	1.123435000
N	-0.327364000	2.762554000	-1.337009000
B	-2.564651000	0.154653000	-1.617418000
H	-1.704626000	0.061127000	-2.423718000
B	-3.369556000	1.682747000	-1.203892000
H	-3.060507000	2.645975000	-1.815275000
B	-3.538603000	1.754088000	0.558273000
H	-3.340079000	2.769091000	1.128172000
B	-4.920300000	1.295483000	-0.445154000
H	-5.848537000	2.026716000	-0.560936000
B	-5.080160000	-0.467668000	-0.390019000
H	-6.125220000	-1.025922000	-0.466661000
B	-3.792947000	-1.103816000	0.644958000
H	-3.779281000	-2.093690000	1.290646000
B	-3.632317000	-1.174314000	-1.117042000
H	-3.511144000	-2.218832000	-1.652749000
B	-2.834065000	0.268519000	1.230941000
H	-2.146326000	0.226318000	2.190710000
B	-4.314627000	0.309241000	-1.804641000
H	-4.795928000	0.321428000	-2.889053000
B	-4.586167000	0.421890000	1.076374000
H	-5.261135000	0.510297000	2.048153000
C	-2.212218000	0.994865000	-0.173918000
C	-2.358566000	-0.645203000	-0.131393000
C	-0.238209000	4.200581000	0.576521000
H	0.828782000	4.420015000	0.700683000
H	-0.819464000	4.969162000	1.091865000
C	-0.600993000	4.139386000	-0.907350000
H	-1.658194000	4.380112000	-1.064305000
H	0.004219000	4.838929000	-1.485712000
C	-0.252068000	2.550014000	2.528807000
H	-0.360038000	1.462581000	2.610057000
C	-1.265347000	3.201898000	3.459789000
H	-1.208696000	4.293204000	3.399420000
H	-1.059132000	2.915065000	4.494628000
H	-2.282284000	2.893929000	3.207859000
C	1.187288000	2.899508000	2.893342000
H	1.892072000	2.429414000	2.202454000
H	1.405692000	2.532899000	3.900077000
H	1.349951000	3.981338000	2.893549000
C	0.395401000	2.436239000	-2.575181000
H	0.389197000	1.342087000	-2.639109000
C	-0.348907000	2.987485000	-3.783521000
H	-1.376593000	2.616495000	-3.806229000
H	0.155001000	2.681768000	-4.704250000
H	-0.376447000	4.081700000	-3.766718000
C	1.847991000	2.897293000	-2.500189000
H	1.915383000	3.985609000	-2.404872000
H	2.385807000	2.607002000	-3.407047000
H	2.347678000	2.440438000	-1.640951000
N	-0.903457000	-2.871479000	-1.053182000
C	-0.913027000	-4.187501000	-0.397949000
H	0.101604000	-4.598996000	-0.342714000
H	-1.544839000	-4.878672000	-0.961027000
C	-1.467530000	-3.955350000	1.007256000
H	-2.562071000	-4.002665000	1.012644000
H	-1.084108000	-4.702775000	1.703705000
C	-0.499406000	-2.304524000	2.743353000
H	-0.321131000	-1.223063000	2.745309000
C	-1.549564000	-2.615572000	3.800954000
H	-2.478961000	-2.079667000	3.592327000
H	-1.186662000	-2.317080000	4.787991000
H	-1.769375000	-3.687235000	3.837024000
C	0.835497000	-2.999223000	2.993033000
H	0.728234000	-4.088183000	2.971934000
H	1.230455000	-2.721326000	3.974297000
H	1.562957000	-2.704901000	2.230471000
C	-0.308465000	-2.718879000	-2.395919000
H	-0.208921000	-1.639051000	-2.554432000
C	-1.247481000	-3.272442000	-3.459392000
H	-1.401074000	-4.348018000	-3.326954000
H	-0.815723000	-3.118357000	-4.452194000
H	-2.219269000	-2.776301000	-3.421155000
C	1.085320000	-3.334514000	-2.475266000
H	1.729834000	-2.948321000	-1.681600000
H	1.542585000	-3.078935000	-3.434703000
H	1.045172000	-4.425587000	-2.407386000
N	-1.012660000	-2.617721000	1.402765000
O	2.737241000	0.277905000	1.271480000
O	2.685839000	-0.666511000	-1.193917000
C	3.853477000	0.087425000	0.695668000
C	5.109873000	0.410607000	1.284554000
C	6.258692000	0.053781000	0.632262000
C	6.228179000	-0.608137000	-0.638826000
C	5.049457000	-0.910627000	-1.264132000
C	3.821803000	-0.532536000	-0.645489000
H	7.222811000	0.264492000	1.083874000
H	7.170660000	-0.863485000	-1.112609000
H	5.016655000	-1.395218000	-2.233877000
H	5.122000000	0.895653000	2.254563000

$(P,P)Au^{+}(O,O)X_4$ (X: Cl or H)
at B3PW91 level without dispersion

2a
Sum of electronic & zero-point Energies -4218.046099
Sum of electronic & thermal Free Energies -4218.126111

Au	-0.095481000	0.000013000	0.000015000
P	1.489675000	-1.688455000	0.013620000
P	1.489645000	1.688502000	-0.013752000
N	1.457979000	-2.796442000	1.241029000
N	1.377891000	-2.747707000	-1.234065000
B	3.602933000	-0.059241000	-1.441094000
H	2.831242000	-0.104483000	-2.334961000
B	4.488043000	-1.475985000	-0.824129000
H	4.324944000	-2.511124000	-1.372763000

B	4.491986000	-1.402268000	0.949661000
H	4.331703000	-2.389027000	1.578643000
B	5.920442000	-0.886331000	0.037865000
H	6.912984000	-1.536939000	0.064798000
B	5.920423000	0.886476000	-0.038248000
H	6.912945000	1.537110000	-0.065236000
B	4.488040000	1.476100000	0.823799000
H	4.324920000	2.511228000	1.372450000
B	4.491914000	1.402372000	-0.949991000
H	4.331594000	2.389116000	-1.578987000
B	3.602975000	0.059320000	1.440766000
H	2.831314000	0.104579000	2.334660000
B	5.368038000	-0.062544000	-1.450017000
H	5.948075000	-0.108402000	-2.483872000
B	5.368082000	0.062673000	1.449645000
H	5.948139000	0.108551000	2.483489000
C	3.168334000	-0.820449000	0.036717000
C	3.168313000	0.820521000	-0.037022000
C	1.139859000	-4.137190000	0.703156000
H	0.065048000	-4.331632000	0.777059000
H	1.681531000	-4.893841000	1.274994000
C	1.592169000	-4.127442000	-0.754712000
H	2.647608000	-4.404893000	-0.850109000
H	0.991714000	-4.818059000	-1.348930000
C	1.114728000	-2.473712000	2.651765000
H	1.264314000	-1.393353000	2.753197000
C	2.063522000	-3.182340000	3.613977000
H	1.951932000	-4.269206000	3.552582000
H	1.824743000	-2.886201000	4.639280000
H	3.106466000	-2.927636000	3.415995000
C	-0.349279000	-2.781385000	2.966636000
H	-1.019917000	-2.334019000	2.227764000
H	-0.595228000	-2.378082000	3.953057000
H	-0.533964000	-3.859361000	2.999848000
C	0.997594000	-2.455156000	-2.637547000
H	0.981164000	-1.362377000	-2.716587000
C	2.039157000	-2.995569000	-3.613353000
H	3.031167000	-2.581495000	-3.419207000
H	1.750977000	-2.732411000	-4.634738000
H	2.103525000	-4.086985000	-3.560438000
C	-0.407214000	-2.973080000	-2.940677000
H	-0.449400000	-4.066274000	-2.907943000
H	-0.696784000	-2.666195000	-3.950037000
H	-1.131897000	-2.572310000	-2.228211000
N	1.457633000	2.796596000	-1.241062000
C	1.139536000	4.137282000	-0.703004000
H	0.064693000	4.331646000	-0.776627000
H	1.681006000	4.894019000	-1.274922000
C	1.592214000	4.127432000	0.754745000
H	2.647669000	4.404901000	0.849889000
H	0.991893000	4.817976000	1.349175000
C	0.997961000	2.454912000	2.637521000
H	0.981862000	1.362121000	2.716448000
C	2.039472000	2.995495000	3.613287000
H	3.031579000	2.581713000	3.418991000
H	1.751449000	2.732115000	4.634662000
H	2.103539000	4.086936000	3.560525000
C	-0.406976000	2.972417000	2.940805000
H	-0.449454000	4.065602000	2.908177000
H	-0.696412000	2.665360000	3.950147000
H	-1.131600000	2.571539000	2.228340000
C	1.114074000	2.473950000	-2.651745000
H	1.263497000	1.393581000	-2.753241000
C	2.062764000	3.182485000	-3.614136000
H	1.951297000	4.269361000	-3.552709000
H	1.823774000	2.886378000	-4.639399000
H	3.105716000	2.927674000	-3.416338000
C	-0.349959000	2.781785000	-2.966340000
H	-1.020507000	2.334468000	-2.227355000
H	-0.596142000	2.378553000	-3.952731000
H	-0.534534000	3.859781000	-2.999475000
N	1.378136000	2.747641000	1.234044000
O	-1.624788000	1.348044000	0.011980000
O	-1.624749000	-1.348048000	-0.011791000
C	-2.804219000	0.704727000	0.004812000
C	-4.018018000	1.397975000	0.007160000
C	-5.235749000	0.700467000	0.003556000
C	-5.235724000	-0.700570000	-0.003371000
C	-4.017970000	-1.398037000	-0.006963000
C	-2.804195000	-0.704755000	-0.004609000
Cl	-3.969732000	-3.130208000	-0.016651000
Cl	-6.730442000	-1.575536000	-0.007919000
Cl	-6.730502000	1.575376000	0.008060000
Cl	-3.969846000	3.130149000	0.016807000

2’a

Sum of electronic & zero-point Energies -4218.031512

Sum of electronic & thermal Free Energies -4218.115301

Au	0.053623000	0.011410000	0.007430000
P	-1.514237000	-1.612954000	-0.620144000
P	-1.543035000	1.604649000	0.623225000
N	-1.542895000	-2.237176000	-2.172329000
N	-1.525960000	-3.073122000	0.157397000
B	-3.686069000	-0.550693000	1.262221000
H	-2.944698000	-0.909388000	2.111586000
B	-4.550608000	-1.662690000	0.173647000
H	-4.407061000	-2.824659000	0.344108000
B	-4.501524000	-0.979317000	-1.462367000
H	-4.322839000	-1.683488000	-2.394693000
B	-5.961702000	-0.807972000	-0.473129000
H	-6.949737000	-1.405154000	-0.754084000
B	-5.976812000	0.826448000	0.212879000
H	-6.975921000	1.430469000	0.433658000
B	-4.524099000	1.671260000	-0.346961000
H	-4.363091000	2.832329000	-0.505886000
B	-4.577724000	0.988658000	1.288776000
H	-4.449871000	1.690470000	2.231645000
B	-3.601158000	0.553691000	-1.381508000
H	-2.803102000	0.906974000	-2.181527000
B	-5.456090000	-0.553181000	1.222715000
H	-6.067486000	-0.953749000	2.158466000
B	-5.370995000	0.568143000	-1.449645000
H	-5.921971000	0.972534000	-2.420611000
C	-3.207316000	-0.766233000	-0.369745000
C	-3.221917000	0.765544000	0.276284000
C	-1.375670000	-3.701510000	-2.143876000
H	-0.322508000	-3.972545000	-2.282617000
H	-1.964549000	-4.156625000	-2.944396000
C	-1.870189000	-4.159101000	-0.774280000
H	-2.951916000	-4.336459000	-0.780329000
H	-1.370408000	-5.080731000	-0.470309000
C	-1.054509000	-1.474280000	-3.346131000
H	-1.137032000	-0.418434000	-3.067296000
C	-1.950169000	-1.715862000	-4.558563000
H	-1.901591000	-2.757535000	-4.891043000
H	-1.614788000	-1.090021000	-5.390528000

H	-2.991395000	-1.469524000	-4.339278000
C	0.415450000	-1.764169000	-3.655791000
H	1.043247000	-1.626600000	-2.770336000
H	0.766040000	-1.075748000	-4.429839000
H	0.558151000	-2.781361000	-4.032815000
C	-1.096455000	-3.342624000	1.545726000
H	-0.975441000	-2.361325000	2.017182000
C	-2.164603000	-4.118180000	2.313523000
H	-3.118878000	-3.586303000	2.322655000
H	-1.840994000	-4.262590000	3.348193000
H	-2.326247000	-5.109305000	1.877229000
C	0.258822000	-4.049060000	1.572260000
H	0.204977000	-5.041242000	1.113176000
H	0.582944000	-4.184764000	2.608217000
H	1.017001000	-3.459427000	1.051389000
N	-1.633141000	2.172218000	2.194285000
C	-1.482054000	3.638298000	2.224421000
H	-0.438174000	3.915011000	2.413923000
H	-2.107283000	4.059334000	3.016043000
C	-1.928064000	4.138208000	0.852483000
H	-3.011772000	4.300293000	0.820766000
H	-1.431025000	5.076915000	0.601636000
C	-1.024250000	3.411280000	-1.453266000
H	-0.862080000	2.446245000	-1.945299000
C	-2.055247000	4.197883000	-2.259434000
H	-2.997110000	3.651735000	-2.348649000
H	-1.669010000	4.385094000	-3.265367000
H	-2.261929000	5.169763000	-1.799377000
C	0.320607000	4.134223000	-1.379403000
H	0.227556000	5.113218000	-0.898642000
H	0.703308000	4.301333000	-2.390449000
H	1.054564000	3.539580000	-0.830332000
C	-1.192437000	1.370679000	3.360926000
H	-1.247295000	0.324914000	3.039515000
C	-2.150405000	1.556260000	4.535116000
H	-2.130810000	2.584981000	4.908399000
H	-1.850592000	0.902467000	5.359165000
H	-3.176361000	1.308840000	4.253329000
C	0.256737000	1.663523000	3.754757000
H	0.933049000	1.555580000	2.901275000
H	0.572111000	0.956210000	4.526972000
H	0.369563000	2.670620000	4.167281000
N	-1.530963000	3.091026000	-0.102241000
O	1.869388000	0.772431000	-1.064506000
O	1.870795000	-0.743895000	1.079191000
C	2.994557000	0.465294000	-0.572492000
C	4.241367000	0.930487000	-1.091339000
C	5.426642000	0.471841000	-0.551034000
C	5.427174000	-0.457812000	0.549909000
C	4.242414000	-0.911099000	1.095935000
C	2.995261000	-0.440509000	0.582952000
Cl	4.200696000	-2.008316000	2.422726000
Cl	6.930631000	-0.994712000	1.188895000
Cl	6.929399000	1.001616000	-1.197589000
Cl	4.198330000	2.027656000	-2.418111000

2a-H

Sum of electronic & zero-point Energies -2379.856651

Sum of electronic & thermal Free Energies -2379.930386

Au	-0.960245000	-0.000019000	0.000146000
P	0.618074000	-1.687674000	0.029772000
P	0.618073000	1.687646000	-0.029930000
N	0.589524000	-2.770505000	1.281914000
N	0.531813000	-2.777924000	-1.195032000
B	2.734908000	-0.068216000	-1.439252000
H	1.963238000	-0.119536000	-2.333779000
B	3.621583000	-1.480361000	-0.815344000
H	3.461405000	-2.518253000	-1.359831000
B	3.625169000	-1.395955000	0.957564000
H	3.466115000	-2.378826000	1.593778000
B	5.054292000	-0.886158000	0.042754000
H	6.047593000	-1.536030000	0.073531000
B	5.054280000	0.886118000	-0.044252000
H	6.047576000	1.535983000	-0.075333000
B	3.621830000	1.480326000	0.814282000
H	3.461790000	2.518199000	1.358839000
B	3.624878000	1.395912000	-0.958636000
H	3.465628000	2.378775000	-1.594824000
B	2.735338000	0.068173000	1.438446000
H	1.963937000	0.119544000	2.333211000
B	4.501218000	-0.071284000	-1.449703000
H	5.082024000	-0.123459000	-2.483211000
B	4.501651000	0.071241000	1.448367000
H	5.082759000	0.123409000	2.481706000
C	2.301884000	-0.821848000	0.041613000
C	2.301879000	0.821801000	-0.042294000
C	0.326926000	-4.132748000	0.772283000
H	-0.740595000	-4.365918000	0.843159000
H	0.891154000	-4.859047000	1.362064000
C	0.785605000	-4.138688000	-0.685314000
H	1.849285000	-4.387059000	-0.769975000
H	0.210635000	-4.860693000	-1.267553000
C	0.194178000	-2.412614000	2.670019000
H	0.274646000	-1.320574000	2.727971000
C	1.161619000	-3.023010000	3.679752000
H	1.126977000	-4.116576000	3.650675000
H	0.879024000	-2.712100000	4.689574000
H	2.189649000	-2.704038000	3.495853000
C	-1.257958000	-2.787765000	2.966269000
H	-1.932619000	-2.390301000	2.202912000
H	-1.543799000	-2.371453000	3.936451000
H	-1.389923000	-3.872503000	3.025507000
C	0.119367000	-2.525633000	-2.596049000
H	0.076564000	-1.435282000	-2.700681000
C	1.154297000	-3.064359000	-3.580044000
H	2.140529000	-2.625890000	-3.410993000
H	0.843477000	-2.828313000	-4.601534000
H	1.242795000	-4.152998000	-3.506200000
C	-1.279668000	-3.080586000	-2.859746000
H	-1.297695000	-4.173268000	-2.794205000
H	-1.594528000	-2.809452000	-3.871914000
H	-1.997276000	-2.671850000	-2.144198000
N	0.589144000	2.770499000	-1.282043000
C	0.326780000	4.132749000	-0.772306000
H	-0.740759000	4.365961000	-0.842800000
H	0.890826000	4.859035000	-1.362275000
C	0.785998000	4.138631000	0.685121000
H	1.849716000	4.386969000	0.769369000
H	0.211288000	4.860635000	1.267615000
C	0.120220000	2.525599000	2.596052000
H	0.077443000	1.435248000	2.700696000
C	1.155402000	3.064295000	3.579810000
H	2.141570000	2.625774000	3.410525000
H	0.844805000	2.828261000	4.601371000
H	1.243936000	4.152930000	3.505945000
C	-1.278735000	3.080576000	2.860065000

H	-1.296789000	4.173252000	2.794409000
H	-1.593313000	2.809560000	3.872351000
H	-1.996530000	2.671755000	2.144757000
C	0.193468000	2.412608000	-2.670047000
H	0.273960000	1.320567000	-2.728057000
C	1.160699000	3.023003000	-3.679974000
H	1.125966000	4.116568000	-3.650973000
H	0.877972000	2.711987000	-4.689726000
H	2.188784000	2.704132000	-3.496198000
C	-1.258723000	2.787803000	-2.965981000
H	-1.933226000	2.390384000	-2.202462000
H	-1.544820000	2.371532000	-3.936104000
H	-1.390651000	3.872549000	-3.025156000
N	0.532424000	2.777850000	1.194952000
O	-2.462554000	1.351518000	0.028173000
O	-2.462637000	-1.351444000	-0.027524000
C	-3.661318000	0.702979000	0.010833000
C	-4.867423000	1.403517000	0.017357000
C	-6.072487000	0.697690000	0.008958000
C	-6.072527000	-0.697408000	-0.007342000
C	-4.867501000	-1.403301000	-0.016075000
C	-3.661355000	-0.702830000	-0.009866000
H	-4.849603000	-2.489324000	-0.030493000
H	-7.011334000	-1.243557000	-0.014187000
H	-7.011264000	1.243890000	0.016050000
H	-4.849466000	2.489540000	0.031761000

2’a-H

Sum of electronic & zero-point Energies -2379.850965
Sum of electronic & thermal Free Energies -2379.929830

Au	-0.941543000	-0.000697000	-0.000204000
P	0.616614000	-1.749344000	0.053621000
P	0.614731000	1.749650000	-0.053687000
N	0.719684000	-2.826312000	1.336717000
N	0.662801000	-2.904332000	-1.136652000
B	2.731395000	-0.061373000	-1.424928000
H	1.964889000	-0.108882000	-2.326374000
B	3.631852000	-1.472242000	-0.818856000
H	3.485963000	-2.503198000	-1.381220000
B	3.635425000	-1.394986000	0.951443000
H	3.487035000	-2.375400000	1.596055000
B	5.065747000	-0.882421000	0.039891000
H	6.061605000	-1.531167000	0.068792000
B	5.064791000	0.887528000	-0.040890000
H	6.059958000	1.537323000	-0.070011000
B	3.630428000	1.475824000	0.818142000
H	3.483521000	2.506674000	1.380440000
B	3.633740000	1.398556000	-0.952164000
H	3.484178000	2.378820000	-1.596723000
B	2.731640000	0.063951000	1.424404000
H	1.965318000	0.110570000	2.326049000
B	4.506485000	-0.063726000	-1.447631000
H	5.088316000	-0.112718000	-2.482352000
B	4.506709000	0.068240000	1.446750000
H	5.088699000	0.117836000	2.481353000
C	2.307224000	-0.838666000	0.040452000
C	2.306341000	0.840829000	-0.040911000
C	0.651695000	-4.218458000	0.862249000
H	-0.373782000	-4.601762000	0.933627000
H	1.305091000	-4.854549000	1.466126000
C	1.108309000	-4.196312000	-0.596581000
H	2.197505000	-4.296193000	-0.672024000
H	0.650431000	-5.013390000	-1.157782000
C	0.199822000	-2.488462000	2.680972000
H	0.220126000	-1.393873000	2.739196000
C	1.120750000	-3.037765000	3.767844000
H	1.134450000	-4.132488000	3.762323000
H	0.763720000	-2.718648000	4.751413000
H	2.144287000	-2.678591000	3.637430000
C	-1.249568000	-2.936900000	2.881933000
H	-1.897403000	-2.554479000	2.087335000
H	-1.621393000	-2.551306000	3.835765000
H	-1.337193000	-4.027223000	2.913559000
C	0.123222000	-2.760411000	-2.501938000
H	-0.063674000	-1.688520000	-2.634529000
C	1.142027000	-3.202466000	-3.550284000
H	2.076191000	-2.643158000	-3.458515000
H	0.737486000	-3.037351000	-4.553092000
H	1.368596000	-4.269803000	-3.457632000
C	-1.210403000	-3.494174000	-2.652701000
H	-1.090077000	-4.576101000	-2.535211000
H	-1.622027000	-3.316729000	-3.650889000
H	-1.935771000	-3.141893000	-1.914247000
N	0.716693000	2.827163000	-1.336473000
C	0.646553000	4.219051000	-0.861568000
H	-0.379497000	4.600847000	-0.932848000
H	1.299003000	4.856300000	-1.465243000
C	1.103140000	4.197150000	0.597256000
H	2.192192000	4.298540000	0.672808000
H	0.644045000	5.013420000	1.158643000
C	0.121001000	2.759215000	2.502513000
H	-0.064368000	1.687038000	2.634884000
C	1.139911000	3.202308000	3.550315000
H	2.074804000	2.644361000	3.457724000
H	0.736265000	3.036220000	4.553323000
H	1.364886000	4.270006000	3.457955000
C	-1.213530000	3.491083000	2.654457000
H	-1.094786000	4.573210000	2.537206000
H	-1.624194000	3.312762000	3.652885000
H	-1.938911000	3.137996000	1.916409000
C	0.197305000	2.489101000	-2.680868000
H	0.219681000	1.394597000	-2.739789000
C	1.117131000	3.040786000	-3.767474000
H	1.128604000	4.135531000	-3.761458000
H	0.760753000	2.721396000	-4.751189000
H	2.141391000	2.683619000	-3.637225000
C	-1.252920000	2.934981000	-2.881523000
H	-1.900098000	2.550813000	-2.087237000
H	-1.624007000	2.549362000	-3.835635000
H	-1.342538000	4.025164000	-2.912399000
N	0.659334000	2.904401000	1.136856000
O	-2.798215000	0.145356000	1.313210000
O	-2.798130000	-0.147558000	-1.313112000
C	-3.917184000	0.098981000	0.741555000
C	-5.170959000	0.214486000	1.426988000
C	-6.327596000	0.106465000	0.712820000
C	-6.327538000	-0.109473000	-0.712888000
C	-5.170838000	-0.217227000	-1.426998000
C	-3.917140000	-0.101476000	-0.741485000
H	-5.164847000	0.371378000	2.500426000
H	-7.284294000	0.180380000	1.220668000
H	-7.284192000	-0.183600000	-1.220787000
H	-5.164634000	-0.374114000	-2.500437000

7. References

[1] M. Joost Synthesis and Original Reactivity of Copper and Gold Complexes, Springer Theses, **2015**, p 183.

[2] M. Joost, A. Zeineddine, L. Estévez, S. Mallet−Ladeira, K. Miqueu, A. Amgoune, D. Bourissou *J. Am. Chem. Soc*. **2014**, *136*, 14654.

[3] V. Kumar, S. Ramanathan, D. Sang, X. Chen, D. M. Lemal *J. Org. Chem*. **2012**, *77*, 966.

[4] Bruker, *SADABS*, Bruker AXS Inc., Madison, Wisconsin, USA.

[5] Sheldrick, G. M. SHELXT – Integrated space-group and crystal-structure determination. *Acta Cryst.* **2015**, *A71*, 3-8.

[6] Sheldrick, G. M. Crystal structure refinement with SHELXL. *Acta Cryst.* **2015**, *C71*, 3-8.

[7] P. G. Jones, D. Villacampa, O. Crespo, M. C. Gimeno, A. Laguna Acta Cryst. C53, 1997, 570.

[8] Spek, A. L. Single-crystal structure validation with the program *PLATON.* J. Appl. Crystallogr., 2003, 36, 7-13.

[9] Ravel, B.; Newville, M. *J. Synchrotron Rad.* **2005**, *12*, 537.

[10] Newville, M. *J. Synchrotron Rad.* **2001**, *8*, 96.

[11] Martin-Diaconescu, V.; Bellucci, M.; Musiani, F.; Ciurli, S.; Maroney, M. J. *J. Biol. Inorg. Chem.* **2012**, *17*, 353.

[12] Zambelli, B.; Berardi, A.; Martin-Diaconescu, V.; Mazzei, L.; Musiani, F.; Maroney, M. J.; Ciurli, S. *J. Biol. Inorg. Chem.* **2014**, *19*, 319.

[13] Newville, M. *J. Phys.: Conf. Series* **2013**, 430, 012007.

[14] Gaussian 16, Revision B.01, M. J. Frisch, G. W. Trucks, H. B. Schlegel, G. E. Scuseria, M. A. Robb, J. R. Cheeseman, G. Scalmani, V. Barone, G. A. Petersson, H. Nakatsuji, X. Li, M. Caricato, A. V. Marenich, J. Bloino, B. G. Janesko, R. Gomperts, B. Mennucci, H. P. Hratchian, J. V. Ortiz, A. F. Izmaylov, J. L. Sonnenberg, D. Williams-Young, F. Ding, F. Lipparini, F. Egidi, J. Goings, B. Peng, A. Petrone, T. Henderson, D. Ranasinghe, V. G. Zakrzewski, J. Gao, N. Rega, G. Zheng, W. Liang, M. Hada, M. Ehara, K. Toyota, R. Fukuda, J. Hasegawa, M. Ishida, T. Nakajima, Y. Honda, O. Kitao, H. Nakai, T. Vreven, K. Throssell, J. A. Montgomery, Jr., J. E. Peralta, F. Ogliaro, M. J. Bearpark, J. J. Heyd, E. N. Brothers, K. N. Kudin, V. N. Staroverov, T. A. Keith, R. Kobayashi, J. Normand, K. Raghavachari, A. P. Rendell, J. C. Burant, S. S. Iyengar, J. Tomasi, M. Cossi, J. M. Millam, M. Klene, C. Adamo, R. Cammi, J. W. Ochterski, R. L. Martin, K. Morokuma, O. Farkas, J. B. Foresman, and D. J. Fox, Gaussian, Inc., Wallingford CT, **2016**.

[15] a) A. D. Becke *J. Chem. Phys.* **1993**, *98*, 5648. b) J. P. Perdew, in *Electronic Structure of Solids '91*, Ed. P. Ziesche and H. Eschrig, Akademie Verlag, Berlin, **1991**, 11.

[16] a) S. Grimme, J. Antony, S. Ehrlich, H. Krieg, *J. Chem. Phys.* **2010**, *132*, 154104. b) S. Grimme, S. Ehrlich, L. Goerigk, *J. Comput. Chem*. **2011**, *32*, 1456.

[17] a) S. Miertuš, E. Scrocco, J. Tomasi, *Chem. Phys.* **1981**, *55*, 117. b) J. L. Pascual-Ahuir, E. Silla, I. Tuñón, *J. Comput. Chem.* **1994**, *15*, 1127. c) V. Barone, M. Cossi, *J. Phys. Chem. A* **1998**, *102*, 1995.

[18] D. Andrae, U. Häussermann, M. Dolg, H. Stoll, H. Preuss, *Theor. Chim. Acta* **1990**, *77*, 123.

[19] A. W. Ehlers, M. Biihme, S. Dapprich, A. Gobbi, A. Hijllwarth, V. Jonas, K. F. Kiihler, R. Stegmann, A. Veldkamp, G. Frenking, *Chem. Phys. Lett.* **1993**, *208*, 111.

[20] a) K. Fukui, *Acc. Chem. Res.* **1981**, *14*, 363. b) H. P. Hratchian, H. B. Schlegel, in *Theory and Applications of Computational Chemistry: The First 40 Years*, Ed. C. E. Dykstra, G. Frenking, K. S. Kim, G. Scuseria, Elsevier, Amsterdam, **2005**, 195.

[21] R. Ahlrichs, M. Baer, M. Haeser, H. Horn, C. Koelmel, *Chem. Phys. Lett.* **1989**, *162*, 165.

[22] a) G. Knizia, *J. Chem. Theory Comput.* **2013**, *9*, 4834. b) G. Knizia, J. Klein, *Angew. Chem. Int. Ed.* **2015**, *54*, 551.

[23] a) E. Reed, L. A. Curtiss, F. Weinhold, *Chem. Rev.* **1988**, *88*, 899. b) J. P. Foster, F. Weinhold, *J. Am. Chem. Soc.* 1980, **102**, 7211. c) A. E. Reed, F. Weinhold, *J. Chem. Phys.* **1985**, *83*, 1736.

[24] NBO 5.0 program, E. D. Glendening, J. K. Badenhoop, A. E. Reed, J. E. Carpenter, J. A. Bohmann, C. M. Morales, F. Weinhold, Theoretical Chemistry Institute, University of Wisconsin, Madison, **2001.**

[25] a) F. London, *J. Phys. Radium* **1937**, *8*, 397. b) R. McWeeny, *Phys. Rev.* **1962**, *126*, 1028. c) R. Ditchfield, *Mol. Phys.* **1974**, *27*, 789. d) K. Wolinski, J. F. Hilton, P. Pulay, *J. Am. Chem. Soc.* **1990**, *112*, 8251. e) J. R. Cheeseman, G. W. Trucks, T. A. Keith, M. J. Frisch, *J. Chem. Phys.* **1996**, *104*, 5497.

[26] W. Kutzelnigg, U. Fleischer, M. Schindler, *The IGLO-Method: Ab Initio Calculation and Interpretation of NMR Chemical Shifts and Magnetic Susceptibilities*, Springer-Verlag, Heidelberg, **1990**, vol. 23.
